# Detailed Phytochemical Analysis of High- and Low Artemisinin-Producing Chemotypes of *Artemisia annua*

**DOI:** 10.3389/fpls.2018.00641

**Published:** 2018-05-18

**Authors:** Tomasz Czechowski, Tony R. Larson, Theresa M. Catania, David Harvey, Cenxi Wei, Michel Essome, Geoffrey D. Brown, Ian A. Graham

**Affiliations:** ^1^Department of Biology, Centre for Novel Agricultural Products, University of York, York, United Kingdom; ^2^Department of Chemistry, University of Reading, Reading, United Kingdom

**Keywords:** *Artemisia annua*, chemotype, artemisinin, NMR, sesquiterpenes, glandular trichomes

## Abstract

Chemical derivatives of artemisinin, a sesquiterpene lactone produced by *Artemisia annua*, are the active ingredient in the most effective treatment for malaria. Comprehensive phytochemical analysis of two contrasting chemotypes of *A. annua* resulted in the characterization of over 80 natural products by NMR, more than 20 of which are novel and described here for the first time. Analysis of high- and low-artemisinin producing (HAP and LAP) chemotypes of *A. annua* confirmed the latter to have a low level of *DBR2* (artemisinic aldehyde Δ^11(13)^ reductase) gene expression. Here we show that the LAP chemotype accumulates high levels of artemisinic acid, arteannuin B, *epi*-deoxyarteannuin B and other amorpha-4,11-diene derived sesquiterpenes which are unsaturated at the 11,13-position. By contrast, the HAP chemotype is rich in sesquiterpenes saturated at the 11,13-position (dihydroartemisinic acid, artemisinin and dihydro-*epi*-deoxyarteannunin B), which is consistent with higher expression levels of *DBR2*, and also with the presence of a HAP-chemotype version of CYP71AV1 (amorpha-4,11-diene C-12 oxidase). Our results indicate that the conversion steps from artemisinic acid to arteannuin B, *epi*-deoxyarteannuin B and artemisitene in the LAP chemotype are non-enzymatic and parallel the non-enzymatic conversion of DHAA to artemisinin and dihyro-*epi*-deoxyarteannuin B in the HAP chemotype. Interestingly, artemisinic acid in the LAP chemotype preferentially converts to arteannuin B rather than the endoperoxide bridge containing artemisitene. In contrast, in the HAP chemotype, DHAA preferentially converts to artemisinin. Broader metabolomic and transcriptomic profiling revealed significantly different terpenoid profiles and related terpenoid gene expression in these two morphologically distinct chemotypes.

## Introduction

Chemical derivatives of the sesquiterpene lactone, artemisinin, such as: artesunate, artemether or dihydroartemisinin are one of several active ingredients in artemisinin-combination therapies (ACTs)—the most effective treatment for malaria currently available. Biosynthesis of artemisinin occurs in specialized 10-celled biseriate glandular trichomes present on the leaves, stems and inflorescences of *Artemisia annua* (Duke and Paul, 1993; Duke et al., 1994; Ferreira and Janick, 1995). Concentrations of artemisinin can range from 0.01 to 1.4% of leaf dry weight (Larson et al., 2013). The biosynthetic pathway from artemisinin precursors has been fully elucidated over the past decade (**Figure 3C**). It starts from the cyclization of farnesyl pyrophosphate (FPP) to amorpha-4,11-diene (A-4,11-D) by amorph-4,11-diene synthase (AMS) (Bouwmeester et al., 1999; Mercke et al., 2000) followed by the three-step oxidation of A-4,11-D by amorpha-4,11-diene C-12 oxidase (CYP71AV1), to artemisinic alcohol (AAOH), artemisinic aldehyde (AAA), and artemisinic acid (AA) (Ro et al., 2006; Teoh et al., 2006). ADH1—NAD-dependent alcohol dehydrogenase with specificity toward artemisinic alcohol plays a role in the formation of artemisinic aldehyde in the artemisinin pathway of *A. annua* (Paddon et al., 2013). The *ADH1* gene has been used to improve yields of artemisinic acid production in yeast (Paddon et al., 2013). Artemisinic aldehyde Δ11(13) reductase (DBR2) catalyzes the formation of dihydroartemisinic aldehyde (DHAAA) from AAA (Zhang et al., 2008). DHAAA is subsequently oxidized in the final enzymatic reaction to dihydroartemisinic acid (DHAA) by aldehyde dehydrogenase ALDH1 (Teoh et al., 2009). Genes encoding all of these biosynthetic enzymes have been shown to be highly expressed in apical and sub-apical cells of *A. annua* glandular trichomes (Olsson et al., 2009; Soetaert et al., 2013). Recent studies have revealed that the conversion of DHAA to artemisinin and dihydro-*epi*-deoxyarteannuin B (DHEDB) proceeds *via* a series of non-enzymatic and spontaneous photochemical reactions, involving the highly reactive tertiary allylic hydroperoxide of dihydroartemisinic acid, DHAAOOH (Wallaart et al., 1999; Sy and Brown, 2002; Brown and Sy, 2004). Similarly, it has previously been proposed that AA is photochemically converted to arteannuin B (ArtB) *via* the tertiary allylic hydroperoxide of artemisinic acid (Brown and Sy, 2007).

Based on the content of artemisinin and its precursors, two contrasting chemotypes of *A. annua* have been described: a low-artemisinin production (LAP) chemotype and a high-artemisinin production (HAP) chemotype (Wallaart et al., 2000). Both chemotypes contain artemisinin, but the HAP chemotype has a relatively high content of DHAA and artemisinin, whereas the LAP chemotype has a high content of AA and ArtB (Lommen et al., 2006; Arsenault et al., 2010; Larson et al., 2013). Recent studies have concluded that a major factor in determining the biochemical phenotype of HAPs and LAPs is the differential expression of *DBR2*—with low expression in LAP chemotypes correlating with a number of insertions/deletions in the *DBR2* promoter sequence (Yang et al., 2015). We have recently shown that the overall pathway to artemisinin biosynthesis is under strict developmental control with early steps in the pathway occurring in young leaves and later steps in mature leaves (Czechowski et al., 2016). In the present study, we have used both metabolomics and transcriptomics to investigate the developmental regulation of sesquiterpene biosynthesis in HAP and LAP chemotypes. Using a combination of NMR and UPLC-/GC-MS techniques we have characterized a number of amorphane and cadinane sesquiterpenes in addition to other terpenes isolated from leaf glandular trichomes. We have also extended the transcript analysis in HAPs and LAPs beyond the genes encoding artemisinin-pathway enzymes. Our findings suggest profound differences in general terpenoid metabolism between HAP and LAP chemotypes that extend well beyond altered *DBR2* expression and artemisinin content.

## Materials and methods

### Plant material

Artemis is an F1 hybrid variety of *A. annua* developed by Mediplant (Conthey, Switzerland), produced by crossing C4 and C1 parental material of East Asian origin (Delabays et al., 2001). Artemisinin content has been reported to reach 1.4% of the leaf dry weight when grown in the field, and its metabolite profile is typical for the HAP chemotype (Larson et al., 2013). NCV (“non-commercial variety”), an “open-pollinated” variety of European origin was also provided by Mediplant, and has the lowest reported artemisinin content from any *A. annua* germplasm in addition to a metabolite profile characteristic of the LAP chemotype (Larson et al., 2013). Plants were grown from seeds in glasshouse conditions as previously described (Graham et al., 2010).

### Leaf area measurements

The leaf area of glasshouse-grown plants was measured by scanning for leaves 14–16 (counting from the apical meristem), followed by calculation of the leaf area using LAMINA software (Bylesjö et al., 2008).

### Trichome density measurements

Trichome density was quantified on the abaxial surface of the terminal leaflets of leaves 14–16 (counting from the apical meristem). Trichomes were visualized using a Zeiss fluorescent dissecting microscope (fitted with a 470/40 nm excitation filter/ 525/50 nm emission filter). Images were recorded using AxioVision 4.7 software (Carl Zeiss Ltd. Herts., UK). Trichome number was counted manually across a 3 × 0.5 mm^2^ leaflet sample area and the average (mean) trichome density was then calculated for the whole leaf.

### NMR structural data for natural compounds from artemis and NCV

Leaf and stem material from Artemis (5 Kg) was extracted in CHCl_3_ (20 L). The organic solvent was removed by rotary evaporation and a portion of the residual dark green aromatic plant extract (*ca* 2.5% w/w) was “dry-loaded” on to a silica column for gradient column chromatography (see Table section Gradient Column Chromatography of the Artemis Variety of *A. annua*).

**Table:** Gradient Column Chromatography of the Artemis Variety of *A. annua*

**Solvent**	**Fraction**
10% EtOAc/hexane	A, B*, C and D*
20% EtOAc/hexane	E, F, G, H, I and J
30% EtOAc/hexane	K, L, M, N and O*
50% EtOAc/hexane	P and Q
EtOAc	R, S, T*, U and V
Methanol	W, X and Y

**Figure 1:**
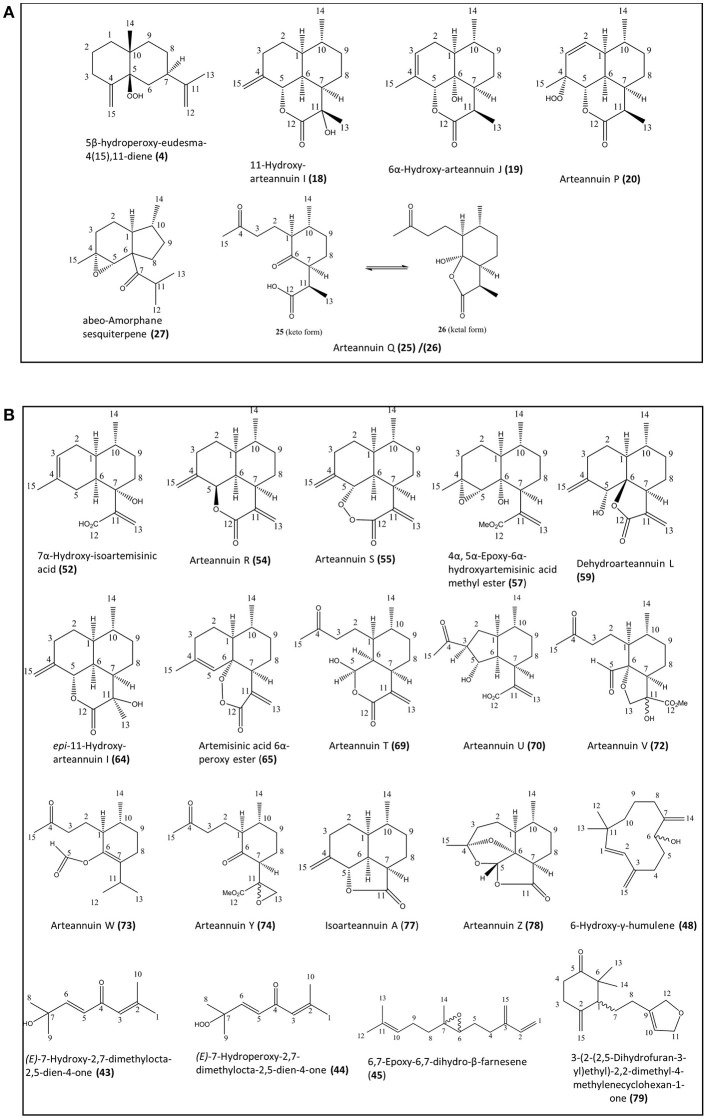
Novel natural compounds characterized from the Artemis **(A)** and NCV **(B)** varieties of *A. annua* by the NMR approach. Numbering of compounds is consistent with Supplementary Lists 1 and 2. Numbering of carbon atoms showed.

Each of the fractions A-Y from gradient column chromatography of Artemis were then further purified by isocratic preparative normal-phase HPLC (^*^fractions B, D, I, O, and T were also subjected to a second round of isocratic column chromatography prior to prep. HPLC); and individual metabolites were then characterized by NMR, as listed in Figure 1A and the Supplemental Table 1 (1D- and 2D-NMR data for all metabolites is also given in the Supplementary List 1). Selected fractions were analyzed by UPLC-APCI-high resolution MS to verify molecular weights and chemical formulae. Confirmed annotations were used to update m/z and retention time reference data, to enable reporting of these compounds from plant extracts by UPLC-MS.

Leaf and stem material from the NCV variety of *A. annua* (780 g) was extracted in CHCl_3_ (4 L). The organic solvent was then removed by rotary evaporation and the residual dark green aromatic plant extract (16.6 g; *ca* 2% w/w) was dry-loaded onto a silica column for gradient column chromatography (see Table section Gradient Column Chromatography of the NCV Variety of *A. annua*).

**Table:** Gradient Column Chromatography of the NCV Variety of *A. annua*

**Solvent**	**Fraction**
2% EtOAc/hexane	A, B and C
10% EtOAc/hexane	D, E, F and G
20% EtOAc/hexane	G, H and I
40% EtOAc/hexane	J, K and L
EtOAc	M and N
Methanol	N

Each of the fractions A-N from gradient column chromatography of NCV were then further purified by isocratic preparative normal-phase HPLC; individual metabolites were then characterized by NMR, as listed in Figure 1B and the Supplemental Table 1 (1D- and 2D-NMR data for all metabolites are also given in the Supplementary List 2). Selected fractions were analyzed by UPLC-APCI-high resolution MS to verify molecular weights and chemical formulae. Confirmed annotations were used to update m/z and retention time reference data, to enable reporting of these compounds from plant extracts by UPLC-MS.

### Metabolite analysis by UPLC-MS and GC-MS

Metabolite analysis by UPLC- and GC-MS were performed as described previously (Czechowski et al., 2016). Fifteen plants from each of five genotype classes were grown from seeds in 4-inch pots under 16 h days for 12 weeks. Metabolite profiles were generated from 50 mg fresh weight (FW) pooled samples of leaves collected at two different developmental stages: 1–5 (counted from the apical meristem), representing the juvenile stage; and leaves 11–13, representing the mature, expanded stage (Figure 3A). Fresh leaf samples were stored at −80°C, pending analysis. In addition, dry leaf material was also obtained from 14-week old plants, cut just above the zone of senescing leaves, and dried for 14 days at 40°C. Leaves were stripped from the plants, and leaf material sieved through 5 mm mesh to remove small stems. Trichome-specific metabolites were extracted as described previously (Czechowski et al., 2016) with minor modifications. Briefly, 50 mg of fresh material was extracted by gentle shaking in 500 μl chloroform for 1 h. Supernatant was taken out and remaining plant material was fully dried in a centrifugal evaporator (GeneVac® Ez-2 plus, Genevac Ltd, Ipswich, UK). Weight of the extracted and dried material was taken and used to quantify abundance of the specific compounds per unit of extracted dry weight. Dry leaf material (0.5 g) was ground to a fine powder using a TissueLyser II ball mill fitted with stainless steel grinding jars (Qiagen, Crawley, UK) operated at 25 Hz for 1 min. Ten mg sub-samples of dry leaf material were extracted in 9:1 (v/v) chloroform:ethanol with gentle shaking for 1 h and then analyzed as per fresh material.

For UPLC-MS analysis of sesquiterpenes, a diluted (1:5 (v/v) extract:ethanol) 2 μL aliquot was injected on an Acquity UPLC system (Waters, Elstree, UK) fitted with a Luna 50 × 2 mm 2.5 μm HST column (Phenomenex, Macclesfield, UK). Metabolites were eluted at 0.6 mL/min and 60°C using a linear gradient from 60 to 100% A:B over 2.5 min, where A = 5% (v/v) aqueous MeOH and B = MeOH, with both A and B containing 0.1% (v/v) formic acid. Pseudomolecular [M+H]^+^ ions were detected using a Thermo Fisher LTQ-Orbitrap (ThermoFisher, Hemel Hempstead, UK) mass spectrometer fitted with an atmospheric pressure chemical ionization source operating in positive ionization mode under the control of Xcalibur 2.1 software. Data was acquired over the m/z range 100–1,000 in FTMS centroid mode with resolution set to 7500 FWHM at m/z 400. Data extraction and analysis was performed using packages and custom scripts in R 3.2.2 (https://www.R-project.org/). XCMS (Smith et al., 2006) incorporating the centWave algorithm (Tautenhahn et al., 2008) was used for untargeted peak extraction. Deisotoping, fragment and adduct removal was performed using CAMERA (Kuhl et al., 2012). Artemisinin was quantified using the standard curve of the response ratio of artemisinin (Sigma, Poole, UK) to internal standard (β-artemether; Hallochem Pharmaceutical, Hong Kong) that was previously added to extracts and standards. Metabolites were identified with reference to authentic standards or NMR-resolved structures and empirical mass formulae calculated using the R package rcdk (Guha, 2007) within 10 ppm error and elemental constraints of: C = 1–100, H = 1–200, O = 0–20, N = 0–1. Peak concentrations were calculated using bracketed response curves, where standard curves were run every ~30 samples. Metabolite concentrations were expressed as a proportion of the residual dry leaf material following extraction.

For analysis of monoterpenes and volatile sesquiterpenes from fresh leaf samples, an aliquot of chloroform extract (prior to dilution with ethanol for UPLC analysis) was taken for GC-MS analysis using an Agilent 6890 GC interfaced to a Leco Pegasus IV TOF MS (Leco, Stockport, UK). A 1 μL aliquot was injected into a CIS4 injector (Gerstel, Mülheim an der Ruhr, Germany) fitted with a 2 mm ID glass liner containing deactivated glass wool at 10°C. The injector was ramped from 10 to 300°C at 12°C/s then held at 300°C for 5 min. The carrier gas was He at a constant flow of 1 mL/min and the injection split ratio was 1:10. Peaks were eluted using a Restek Rxi-5Sil MS column, 30 m × 0.25 mm ID × 0.25 μm film thickness (Thames Restek, Saunderton, UK). The following temperature gradient was used: isothermal 40°C 2 min; ramp at 20°C/min to 320°C then hold for 1 min; total run time ~20 min. The transfer line was maintained at 250°C and the MS used to collect−70 eV EI scans over the m/z range 20–450 at a scan rate of 20 spectra/s. Acquisition was controlled by ChromaTof 4.5 software (Leco). ChromaTof was used to identify peaks and deconvolute spectra from each run, assuming a peak width of 3 s and a minimum *s/n* of 10. Peak areas were reported as deconvoluted total ion traces (DTIC). Further analyses including annotation against authentic standards, between-sample peak alignment, grouping, consensus DTIC reporting, and missing value imputation were performed using custom scripts in R.

R was used for all statistical data analysis using the stats base package, nlme (http://CRAN.R-project.org/package=nlme) and pcaMethods (Stacklies et al., 2007).

### RNA isolation, cDNA synthesis, and quantitative RT-PCR

Leaf tissue from juvenile and mature-stage leaves sampled as described above was ground to a fine powder using Qiagen Retsch MM300 TissueLyser (Qiagen, Hilden, Germany) and total RNA extracted using the RNAeasy kit (Qiagen, Hilden, Germany). RNA was quantified using NanoDrop-1000 (NanoDrop products, Wilmington, USA) and integrity was checked on 2200 Tape Station Instrument (Agilent, Santa Clara, CA, USA). Only samples scoring RIN number ≥7.0 were taken for further analysis. Removal of genomic DNA was performed by treating with TURBO DNA-free™ (Life Technologies Ltd, Paisley, UK) following manufacturer's instructions. 5 ug of total RNA, pooled from 4 individual plants, representing 3 biological replicates, was reversely transcribed using SuperScript II kit (Life Technologies Ltd, Paisley, UK) and Oligo(dT)12-18 Primer (Life Technologies Ltd, Paisley, UK) according to manufacturer's instructions. PCR using primers (AMS_Ex4 for 5′-GGCTGTCTCTGCACCTCCTC-3′, AMS_Ex5 for 5′- CAGCCATCAATAACGGCCTTG-3′) designed spanning intron 4 of the *AMS* gene (GenBank: AF327527). Only samples that resulted in amplification of the 251 bp fragment from cDNA and not the 363 bp fragment from genomic DNA were taken for further qPCR analysis.

Expression levels of amorpha-4,11-diene synthase (AMS), amorpha-4,11-diene C-12 oxidase (CYP71AV1), cytochrome P450 reductase (CPR), artemisinic aldehyde Δ 11 (13) reductase (DBR2) and aldehyde dehydrogenase (ALDH1), relative to ubiquitin (UBI) were determined by qPCR. Reactions were run in 3 technical replicates. Gene-specific primers used were: AMS for 5′- GGGAGATCAGTTTCTCATCTATGAA- 3′; AMS_Rev 5′- CTTTTAGTAGTTGCCGCACTTCTT-3′; 5′ALDH1 for 5′- GATGTGTGTGGCAGGGTCTC-3′; ALDH1_Rev 5- ACGAGTGGCGAGATCAAAAG-3′; CYP71AV1 for 5′- TCAACTGGAAACTCCCCAvcATG-3′; CYP71AV1_Rev 5′- CGGTCATGTCGATCTGGTCA-3′; CPR_For 5′- GCTCGGAACAGCCATCTTATTCTT-3′, CPR_Rev 5′- GAAGCCTTCTGAGTCATCTTGTGT-3′, DBR2 for 5′- GAACGGACGAATATGGTGGG-3′; DBR2_Rev 5′- GCAGTATGAATTTGCAGCGGT-3′, UBI for 5′-TGATTGGCGTCGTCTTCGA-3′ and UBI_Rev 5′-CCCATCCTCCATTTCTAGCTCAT-3′. Reactions conditions and qPCR analysis were performed as above, 1 ul of 1/20 first strand cDNA dilution was used instead of genomic DNA. Background subtraction, average PCR efficiency for each amplicon and N0 values were calculated using LinRegPCR ver. 2012 software (Ruijter et al., 2009). Expression levels for each sample and gene of interest (GOI) were represented as N0 GOI/N0 UBI.

## Results

### NMR spectroscopic analysis uncovers novel metabolites in both HAP and LAP chemotypes

**Figure 2:**
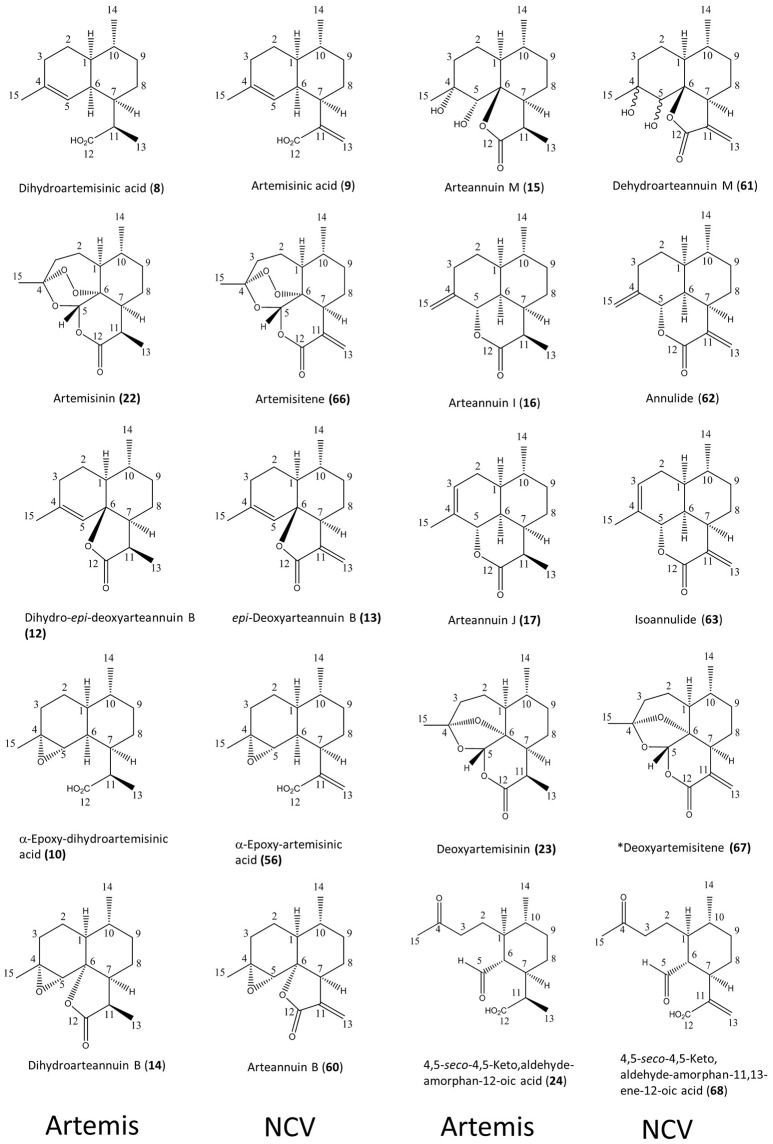
Ten pairs of 11,13-dihdyro/ 11,13-dehydro amorphanolides between Artemis (left-hand side) and NCV (right-hand side) varieties of *A. annua* characterized by the NMR approach. Numbering of compounds is consistent with Supplementary Lists 1, 2. Numbering of carbon atoms showed. Novel compound indicated by asterisk.

The natural products found in *A. annua* have previously been grouped into eight broad categories, including: (i) monoterpenes; (ii) sesquiterpenes; (iii) diterpenes, (iv) sterols and triterpenes; (v) aliphatic hydrocarbons, alcohols, aldehydes and acids; (vi) aromatic alcohols, ketones and acids; (vii) phenylpropanoids; and (viii) flavonoids (Brown, 2010). In the present work we have used the Artemis variety of *A. annua* as a representative of the HAP chemotype and NCV as a representative of the LAP chemotype (Larson et al., 2013). Our initial investigations using NMR analysis of leaf extracts of Artemis resulted in the isolation of 41 metabolites (6 of which were novel) representing all eight classes of natural products (Figure 1A, Supplementary List 1). The structures of all compounds were determined by 1D- and 2D- NMR spectroscopy (detailed NMR data in Supplementary Section). Novel compounds which have not been isolated before as natural products include four new 11,13-dihydroamorphanes: 5β-hydroperoxy-eudesma-4(15),11-diene **(4)**, 11-hydroxy-arteannuin I **(18)**, 6α-hydroxy-arteannuin J **(19)**, arteannuin P **(20)**, the ketal form of arteannuin Q **(26)** and abeo-amorphane sesquiterpene **(27)**. Artemisinin **(22)** was the most abundant metabolite in this analysis (Figure 2, Supplementary List 1, and Supplemental Table 1); but the Artemis extract also contained two other sesquiterpenes: dihydroartemisinic acid (DHAA, **8**), and dihydro-*epi*-deoxyarteannuin B (DHEDB, **12**) in substantial amounts (Figure 2, Supplementary List 1 and Supplemental Table 1). In addition, a further nine known 11,13-dihydroamorphanoic acid derivatives (α-epoxy-dihydroartemisinic acid **(10)**; 4α,5α-epoxy-6α-hydroxyamorphan-12-oic acid **(11)**; dihydroarteannuin B **(14)**; arteannuin M **(15)**; arteannuins H, I and J (**21, 16**, and **17**); deoxyartemisinin **(23)**; and a 4,5-*seco*-4,5-diketo-amorphan-12-oic acid **(24)** (see Figure 1A, Supplementary List 1 and Supplemental Table 1) were also isolated as minor components from the Artemis leaf extracts (Figure 2, Supplementary List 1 and Supplemental Table 1).

Phytochemical investigation of the NCV variety by NMR yielded 57 metabolites, 20 of which were novel (Figure 1B and Supplementary List 2), representing 7 of the 8 categories above. Novel metabolites from the NCV variety are depicted in Figure 1B and include: *(E*)-7-hydroxy-2,7-dimethylocta-2,5-dien-4-one (**43**), (*E*)-7-hydroperoxy-2,7-dimethylocta-2,5-dien-4-one (**44**), 6,7-epoxy-6,7-dihydro-β-farnesene (**45**), 6-hydroxy-γ-humulene (**48**), 7α-hydroxy-artemisinic acid (**52**), arteannuin R (**54**), arteannuin S (**55**), 4α, 5α-epoxy-6α-hydroxyartemisinic acid methyl ester (**57**), dehydroarteannuin L (**59**), *epi*-11-hydroxy-arteannuin I (**64**), artemisinic acid, 6α-peroxy ester (**65**), deoxyartemistene (**67**), arteannuin T (**69**), arteannuin U (**70**), arteannuin V (**72**), arteannuin W (**73**), arteannuin Y (**74**), isoarteannuin A (**77**), arteannuin Z (**78**), and 3-(2-(2,5-dihydrofuran-3-yl)ethyl)-2,2-dimethyl-4-methylenecyclohexan-1-one (**79**).

As might have been expected, the most striking difference between the NCV and *Artemis* varieties was the almost complete absence of artemisinin, dihydroartemisinic acid (DHAA, (**8**)) and dihydro-epi-deoxyarteannuin B (DHEDB, (**12**)) in the former (Supplemental Table 1). The NCV variety did, however, have relatively high levels of three 11-13-unsaturated amorphanes, which were found only as minor components in the *Artemis* variety, namely: artemisinic acid (AA, **9**), arteannuin B (ArtB, **60**) and *epi*-deoxyarteannuin B (EDB, **13**) (Figure 2 and Supplemental Table 1). All the other amorphane sesquiterpenes isolated and characterized from the NCV variety by NMR shared this same trait: i.e., possession of an 11,13-unsaturated methylene group (Figures 1B, 2 and Supplemental Table 1), and there is an almost complete absence of 11,13-dihydro-amorphanes from NCV, that contrasts with the abundance of these compounds in the *Artemis* variety (Supplementary List 2 and Supplemental Table 1). It is interesting to note that there are ten examples where 11,13-dihdyro/ 11,13-dehydro amorphanolides seem to occur as “pairs” between *Artemis* and NCV as depicted in Figure 2. These include: DHAA **(8)**/AA **(9)**; artemisinin **(22)**/artemisitene **(66)**; dihydro-*epi*-deoxyarteannuin B **(12)**/*epi*-deoxyarteannuin B **(13)**; α-epoxy-dihydroartemisinic acid **(10)**/α-epoxy-artemisinic acid **(56)**; dihydroarteannuin B **(14)**/arteannuin B **(60);** arteannuin M **(15)**/dehydroarteannuin M **(61)**; arteannuin I **(16)**/annulide **(62)**; arteannuin J **(17)**/isoannulide **(63)**; deoxyartemsinin **(23)**/deoxyartemsitene **(67)**; and 4,5-*seco*-4,5-diketo-amorphan-12-oic acid **(24)** and its 11,13-dehydro-analog **(68)**. It is also noteworthy that 9 of the 20 novel amorphane and *seco-*amorphane sesquiterpenes isolated and characterized from the NCV variety by NMR, possess an 11, 13-unsaturated methylene group (Figures 1B, 2 and Supplementary List 2).

All the above results are consistent with a higher DBR2 activity in the HAP chemotype compared to the LAP chemotype (Yang et al., 2015). The relative abundances for 8 of these 10 “pairs” are also well matched between the *Artemis* and NCV varieties, suggesting a “shared” further metabolism for DHAA in *Artemis* and AA in NCV. The first exception is arteannuin B (ArtB **60**), which is abundant in NCV, whilst its analog, dihydroarteannuin B (**14**), is relatively low in *Artemis* (Supplemental Table 1). The second is artemisitene, the 11,13-dehydro analoge of artemisinin (Acton and Klayman, 1985; Woerdenbag et al., 1994; Figure 1; Supplemental Table 1) which is a minor compound in NCV, while its “partner” artemisinin is the most abundant metabolite in *Artemis* (Supplemental Table 1). These observations suggest that while there are many parallels in the pathways that further transform DHAA (**8**) and AA (**9**) in the HAP and LAP chemotypes there are also some significant differences.

### Metabolomic and gene expression studies reveal multiple differences between HAP and LAP chemotypes

**Figure 3:**
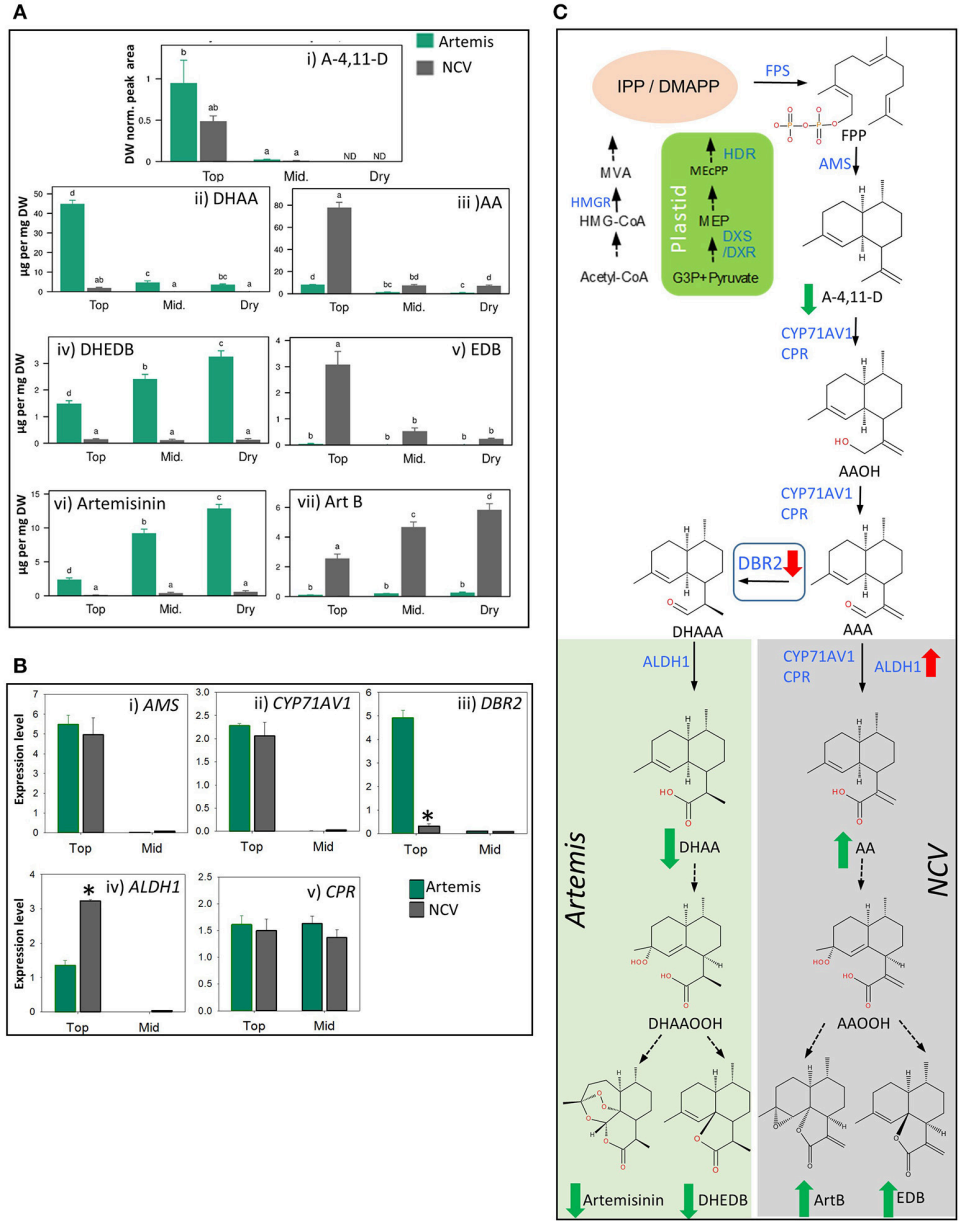
Metabolic and transcriptomic comparison of the artemisinin pathway in the low- vs. high-artemisinin chemotypes of *A. annua*. **(A)** Level of selected sesquiterpenes were quantified by GC-MS **(i)** and UPLC-MS **(ii–vii)** in fresh juvenile leaf 1–5 (Top), fresh mature leaf 11–13 (Mid.) and oven-dried whole plant-stripped leaves (Dry) from 12-weeks old glasshouse-grown Artemis (green bars) and NCV (gray bars) varieties as described in Materials and methods. error bars—SEM (*n* = 15 for Top and Mid. leaf; *n* = 6 for Dry leaf). Letters represent Tukey's range test results after one way ANOVA or REML (see Materials and Methods for details). Groups not sharing letters indicate statistically significant differences. **(B)** Transcript profiling of enzymes involved in the artemisinin biosynthetic pathway, in two types of leaf material as on **(A)** was done as described in Materials and Methods, error bars—SE (*n* = 9). Asterisk indicates *t*-test statically significant difference between Artemis (green bars) and NCV (gray bars) at *p* < 0.05. **(C)** Summary of the metabolite and transcriptional differences between Artemis and NCV for the artemisinin biosynthetic pathway: full arrows—known enzymatic steps, dashed arrows—non-enzymatic conversions, red arrows—transcript changes in juvenile leaves of NCV vs. Artemis, green arrows—metabolite changes of NCV vs. Artemis (all types of leaves). DBR2 position in the pathway highlighted in a square. Metabolite abbreviations: G-3-P, glyceraldehyde-3-phosphate; MEP, 2-C-methylerythritol 4-phosphate; MEcPP, 2-C-methyl-D-erythritol-2,4-cyclopyrophosphate. Cytosolic precursors: HMG-CoA, 3-hydroxy-3-methylglutaryl-CoA; MVA, mevalonate; IPP, isopentenyl pyrophosphate; DMAPP, dimethylallyl pyrophosphate; FPP, farnesyl pyrophosphate; A-4,11-D, amorpha-4,11-diene; AAOH, artemsinic alcohol; AAA, artemsinic aldehyde; AA, artemsinic acid; ArtB, arteannuin B; DHAAA - dihydroartemsinic aldehyde; DHAA, dihydroartemsinic acid; DHAAOOH, dihydroartemsinic acid tertiary hydroperoxide; DHEDB, dihydro-*epi*-deoxyarteanniun B; AAOOH, artemsinic acid tertiary hydroperoxide; EDB, *epi*-deoxyarteannuin B. Enzyme abbreviations: HMGR-, 3-hydroxy-3-methylglutaryl coenzyme A reductase; HDR-, 4-hydroxy-3-methylbut-2-enyl diphosphate reductase; DXR, 1-deoxy-D-xylulose-5-phosphate reductoisomerase; DXS-, 1-deoxy-D-xylulose-5-phosphate synthase; FPS, farnesyl diphosphate synthase. AMS, amorpha-4,11-diene synthase; CYP71AV1, amorpha-4,11-diene C-12 oxidase; CPR, cytochrome P450 reductase; DBR2, artemisinic aldehyde Δ 11 (13) reductase; ALDH1, aldehyde dehydrogenase.

Using a leaf maturation time-series, we recently demonstrated that artemisinin levels increase gradually from juvenile to mature leaves and remain stable during the post-harvest drying process in Artemis HAP chemotype plants (Czechowski et al., 2016). Using a similar time-series (which included fresh leaf 1–5 (juvenile), and 11–13 (mature) (counting from the apical meristem); plus oven-dried whole plant-stripped leaves (dry) from 12-week-old glasshouse-grown plants), we have now performed UPLC- and GC-MS based metabolite profiling of extracts from both HAP (Artemis*)* and LAP (NCV) chemotypes. We found that the pathway entry–point metabolite, amorpha-4,11-diene (A-4,11-D), is only detectable in juvenile leaves, and at approximately 2-fold higher concentration in Artemis as compared to NCV (Figure 3Ai; Supplemental Table 3). A much greater difference was seen for the enzymatically-produced artemisinin precursor, dihydroartemisinic acid (DHAA), which was present at a 24-fold higher concentration in juvenile Artemis leaves compared to NCV (Figure 3Aii), Supplemental Table 2). Artemisinic acid (AA) on the other hand accumulated in NCV leaves at a 10-fold higher concentration than in Artemis (Figure 3Aiii), Supplemental Table 1). Interestingly the levels of AA in the young leaves of NCV variety are approximately twice the levels of DHAA in young leaves of Artemis (Figures 3Aii,iii), Supplemental Table 2). The levels of both DHAA and AA dropped sharply beyond the juvenile leaf stage in Artemis and NCV, respectively (Figures 3Aii,iii), Supplemental Table 2). These changes in metabolite levels occur during leaf maturation are mirrored by changes in steady state mRNA levels of genes encoding the enzymes involved in their biosynthesis including: amorpha-4,11-diene synthase (AMS), amorpha-4,11-diene C-12 oxidase (CYP71AV1), artemisinic aldehyde Δ^11, (13)^ reductase (DBR2) and aldehyde dehydrogenase (ALDH1) which are expressed at levels two to three orders of magnitude higher in juvenile than in mature leaves (Figures 3Bi–iv).

Previous work has suggested that *in vivo* conversions beyond DHAA (**8**) (Czechowski et al., 2016) and *in vitro* conversions beyond AA (**9**) (Brown and Sy, 2007) are non-enzymatic. Consistent with this, we have found that mature leaves of NCV contain high levels of *epi*-deoxyarteannuin B (EDB, **13**) and arteannuin B (ArtB, **60**) (Figures 3Av,vii), Supplemental Table 2), while Artemis accumulates dihydro-*epi*-deoxyarteannuin B (DHEDB, **12**) and artemisinin (**22**) (Figures 3Aiv,vi) Supplemental Table 2) at 20–30-fold higher levels than NCV. Both artemisinin (**22**) and arteannuin B (**60**) continue to accumulate in the post-harvest drying process in Artemis and NCV respectively (Figures 3Avi,vii). Post-harvest accumulation of artemisinin has been reported before (Ferreira and Luthria, 2010) and it might be related to light-dependent conversion of DHAA. However slightly different batch specific environmental effects during drying might explain the difference between the artemisinin accumulation pattern shown in Figure 3Avi) and that which was previously reported for the Artemis variety (Czechowski et al., 2016). Interestingly, the developmental pattern of DHEDB (**12**) accumulation in Artemis leaves is different to its 11,13-dehydro analog, EDB (**13**) in NCV leaves. DHEDB (**12**) follows the same accumulation pattern as for artemisinin (**22**) in Artemis (Figures 3Aiv,vi); whereas EDB (**13**) is found predominantly in juvenile leaves of the NCV variety (Figure 3Av). We have found that production of the artemisinin 11,13-dehydro analog, artemisitene (**66**) in NCV parallels the accumulation of artemisinin (**22**) in Artemis (Supplemental Table 2), albeit at very much reduced levels. The levels of deoxyartemisinin (**23**), another product of non-enzymatic conversion of DHAA through the DHAA allylic hydroperoxide, increase during dry leaf storage, accumulating to 0.1% leaf dry weight (Supplemental Table 2), which is consistent with previous findings (Czechowski et al., 2016). This process is paralleled by accumulation of deoxyartemisitene (**67**) (the 11,13-dehydro analog of deoxyartemisinin) in the NCV variety (Supplemental Table 2).

RT-qPCR analysis confirmed the expression level for *DBR2* to be significantly repressed (8-fold lower) in the juvenile leaves of NCV compared to Artemis, which is consistent with previous findings (Yang et al., 2015). Interestingly, *DBR2* transcript abundance had decreased to the same levels in mature leaves of both chemotypes (Figure 3Biii), highlighting the importance of developmental timing in regulating flux and partitioning of sesquiterpene metabolites. More surprisingly, *ALDH1* expression is increased in juvenile leaves (2.4-fold) and further increased in mature leaves (40-fold) of NCV (Figure 3Biv) compared to Artemis. Thus it would appear that in addition to *DBR2* being down-regulated in the NCV (LAP) chemotype, *ALDH1* is up-regulated at the transcriptional level. This could also account for the increase in flux into artemisinic acid and the arteannuin B branch of sesquiterpene metabolism. The major differences in metabolite levels and gene expression between Artemis and NCV varieties for the artemisinin biosynthetic pathway are summarized in Figure 3C.

**Figure 4:**
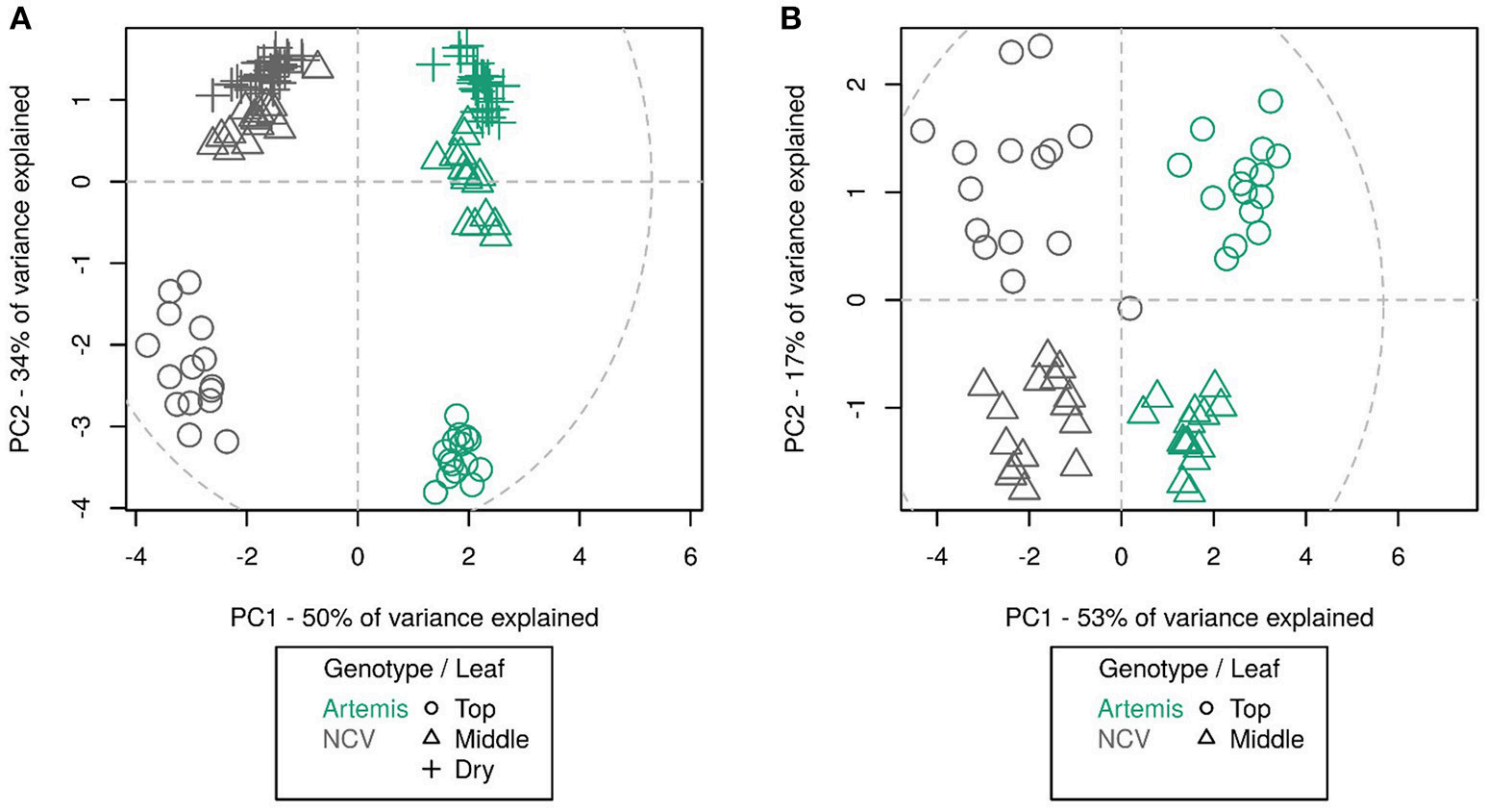
Principal component analysis of UPLC-MS **(A)** and GC-MS **(B)** data from different leaf types from Artemis and NCV varieties. Principal component analysis of 75 UPLC-MS identified peaks **(A)** and 202 GC-MS identified metabolites **(B)**. Leaf types, corresponding with Figure 3 are represented by symbols: circles—leaf 1–5, triangles—leaf 11–13, crosshairs—oven-dried leaf. Two chemotypes represented by colors green—Artemis and gray—NCV. PCA was performed on log-scaled data and mean-centered data; dotted ellipse = Hotelling's 95% confidence interval.

NMR analysis revealed that metabolite differences between Artemis and NCV are not restricted to artemisinin-related sesquiterpenes. Monoterpenes also vary between the two chemotypes, with for example camphor being most abundant in Artemis while artemisia ketone level is much more abundant in NCV (Supplemental Table 1). Unfortunately, NMR-analysis could only provide approximate information about the relative abundance of the metabolites, therefore metabolite content of both chemotypes was also studied by GC- and UPLC-MS (Supplemental Tables 2, 3). We were able to detect 75 unique compounds in three leaf types by UPLC-MS of which annotations were assigned to 30 compounds based on NMR-verified standards as described in the Materials and Methods. The majority of the known compounds were sesquiterpenes and flavonoids. GC-MS detected 202 unique compounds in juvenile and mature leaves, of which 33 had assigned annotations. The majority of known GC-MS-detected compounds were mono- and sesquiterpenes. Using principal component analysis, it can be seen that the overall metabolite profile of NCV appears strikingly different to that of Artemis; as much as the difference between the profiles between juvenile leaves and mature- and/or dry leaves. In fact, UPLC- and GC-MS PCA plots show four distinct clusters (Figures 4A and B). Developmental differences are most apparent in juvenile leaf tissue, which show the highest abundance of most of the terpenes described below (Figure 4, Supplemental Tables 2 and 3). Our findings that the metabolite profiles in Artemis and NCV young leaf tissues are considerably different to mature and dry leaves in both varieties are consistent with our previous findings (Czechowski et al., 2016).

There are a number of compounds specifically produced by NCV, mostly in low quantities (Supplemental Tables 2 and 3) which have known medicinal use including, for example, isofraxidin **(39)**, which is five-fold more abundant in the juvenile leaves of NCV as compared to Artemis (Supplemental Table 2). Isofraxidin is a coumarin with anti-inflammatory (Niu et al., 2012) and anti-tumor activities (Yamazaki and Tokiwa, 2010). Artemisia ketone **(42)**, an irregular monoterpene found in the essential oil from various *A. annua* varieties displaying antifungal activities (Santomauro et al., 2016) is the most abundant volatile in the juvenile and mature leaves of NCV, but virtually absent in Artemis (Supplemental Table 3). The juvenile and mature leaves of Artemis accumulate velleral, a sesquiterpene dialdehyde which has proposed antibacterial activities (Anke and Sterner, 1991), which is virtually absent in the NCV variety (Supplemental Table 3). GC-MS analysis further revealed that several major montoerpenes are also more abundant in juvenile and mature leaves of Artemis, including camphor (3.7-fold higher), camphene (3.4-fold higher), borneol, (16-fold higher), α-pinene (4.6-fold higher) and 1,8-cineole (8-fold higher) (Supplemental Table 3). Some minor monoterpenes detected in the Artemis variety, such as: α-myrcene, α –terpinene, chrysanthenone and α-copaene, are virtually absent in young and mature NCV leaves (Supplemental Table 3). A few striking differences were noted for the level of artemisinin-unrelated abundant sesquiterpenes, such as sabinene and *cis*-sabinene hydrate, which are 7.5- and 38-fold (respectively) more abundant in Artemis young leaves than in NCV (Supplemental Table 3). Germacrene A is a sesquiterpene common across the Asteraceae family for which it has been demonstrated that its downstream metabolism parallels artemisinic acid biosynthetic pathway (Nguyen et al., 2010). Germacrene A levels are 32- and 17-fold higher in NCV young and mature leaves (respectively) making it the most abundant volatile in mature and the second most abundant in young leaves of the NCV variety.

**Figure 5:**
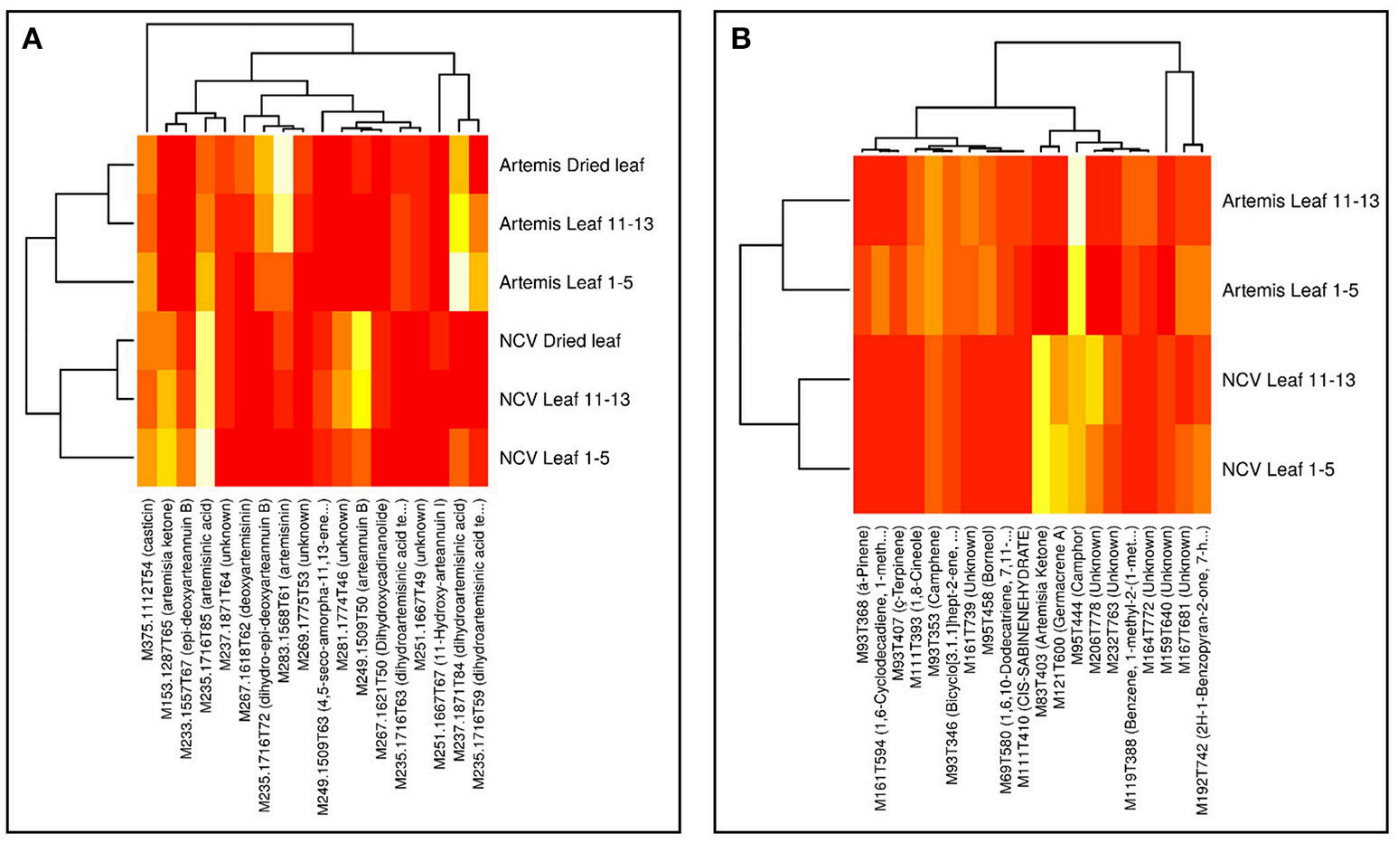
Heatmaps of influential metabolites from UPLC- and GC-MS PCA analyses. Top-n [UPLC-MS = 18 **(A)**; GC-MS = 20 **(B)**] metabolites were chosen for visualization based on loadings plots (Supplementary Figure 1) from the PC1 dimensions in the PCA analyses (Supplementary Figure 1). Mean data were log-scaled and then row-scaled for color intensity plotting (lighter = more abundant). Hierarchical clustering was performed with average linkage, with Euclidean distances for genotypes and 1-absolute values of correlations as distances for metabolites. Metabolite names are abbreviated where necessary for clarity and are given in full in Supplementary Tables 2, 3.

Visualization of the loadings from the multivariate analyses were used to identify the most influential compounds discriminating chemotypes. PC1 loading plots identified 18 compounds from UPLC- and 20 from GC-MS analysis (Supplementary Figure 1), which were used to create the heatmaps presented in Figure 5. The vast majority of the most influential compounds distinguishing between two chemotypes from UPLC-MS analysis were the amorphane sesquiterpenes (Figure 5A). The mono- and sesquiterpenes mentioned above (together with some unknown compounds) were the most influential GC-MS-detectable metabolites distinguishing between two chemotypes (Figure 5B).

### Morphological difference between two chemotypes of *A. annua*

**Figure 6:**
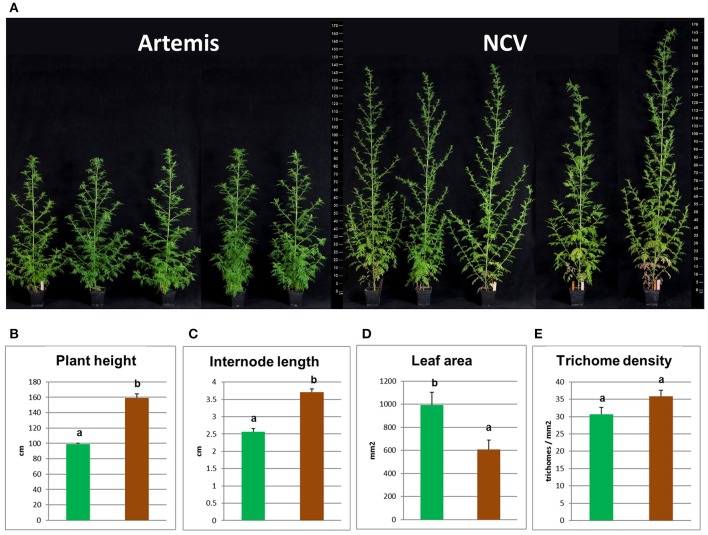
Morphological characterization of low- and high-artemisinin natural chemotypes of *A. annua*. **(A)** Photographs show four representative 12-week old plants from each two chemotypes of *A. annua*, ruler scaled in cm showed on both sides; Plant height **(B)**, internode length **(C)**, leaf area **(D)**, and glandular secretory trichome density **(E)** recorded for 12-week old plants. Green bars represent Artemis (HAP-chemotype) and brown bars represent NCV (LAP-chemotype). Error bars—SEM (*n* = 15), letters represent one-way ANOVA Tukey's range test results; Groups not sharing letters indicate statistically significant differences.

In addition to having very distinct phytochemical compositions the F1 *Artemis* HAP chemotype and the open pollinated NCV LAP chemotype varieties also have very distinct morphological features (Figure 6). Most strikingly, NCV is much taller with longer internodes but produces smaller leaves than *Artemis*. The density of glandular secretory trichomes, the site of artemisinin synthesis, is similar for both varieties (Figure 6E), which is consistent with the main difference in artemisinin production being due to an alteration in metabolism rather than trichome density. *A. annua* varieties typically require short day length for flowering (Wetzstein et al., 2014), but we observed that NCV, unlike *Artemis*, can also flower under long days. However, the two chemotypes do cross-pollinate and produce viable progeny.

## Discussion

This manuscript presents the first detailed phytochemical comparison of high- (HAP) and low-artemisinin producing (LAP) chemotypes chemotypes of *A. annua*.

Twenty six of the 85 metabolites that have been characterized by NMR from the HAP and LAP varieties of *A. annua* in this study are novel as natural products (all are mono- and sesquiterpenes). And of these, 19 are amorphane sesquiterpenes, which is the most diverse and the most abundant sub-class (Supplemental Table 1, Supplementary Lists 1 and 2). The majority of these amorphane sesquiterpenes are highly oxygenated with structures that would be consistent with further oxidative metabolism of DHAA (11,13-saturated, **8**) in the HAP variety and AA (11,13-unsaturated, **9**) in the LAP variety (Figures 1 and 2, Supplemental Table 1, Supplementary Lists 1 and 2).

UPLC- and GC-MS analysis of leaf developmental series also revealed amorphanes either saturated or unsaturated at the 11,13-position in the HAP and LAP chemotypes, respectively (Figure 3, Supplemental Table 2). This observation is consistent with the expression of the *DBR*2 gene, which encodes the enzyme responsible for reducing the 11,13-double bond of artemisinic aldehyde (the precursor for 11,13-dihydroamorphane/cadinane sesquiterpenes), being strongly down-regulated in juvenile leaves of NCV (Figure 3Biii). These findings are in complete agreement with the recent report on reduced levels of *DBR2* in LAP compared with HAP chemotypes (Yang et al., 2015). In addition to altered expression of *DBR2*, we also found that expression of *aldehyde dehydrogenase* (*ALDH1*), which converts artemsinic and dihydroartemsinic aldehydes to their respective acids (Teoh et al., 2009), is significantly elevated in juvenile and mature leaves of NCV compared to Artemis. This may lead to an increased flux from A-4,11-D to AA (8) in NCV when compared with flux from A-4,11-D to DHAA (9) in Artemis which is reflected by a significantly higher concentration of AA found in juvenile leaves of NCV when compared to the concentration of DHAA in young Artemis leaves (Figures 3Aii,iii). The elevated flux from A-4,11-D to AA (8) might also explain lower levels of A-4,11-D found in juvenile leaves of NCV when compared with Artemis (Figure 3Ai) as the expression of *amorpha-4,11-diene synthase (AMS)* is at very similar level in both varieties (Figure 3Bi). We have also observed that the NCV (LAP) variety expresses a sequence variant of amorpha-4,11-diene C-12 oxidase (*CYP71AV1*) with a 7 amino acid N-extension (Supplementary Figure 2). This LAP-chemotype associated sequence variant upon transient expression in *Nicotiana benthamiana*, in combination with the other artemisinin pathway genes resulted in a qualitatively different product profile (“chemotype”); that is a shift in the ratio between the unsaturated and saturated (dihydro) branch of the pathway (Ting et al., 2013). That result strongly suggests the two distinct isoforms of CYP71AV1 are associated with HAP- and LAP-branches of the artemisinin pathway in *Artemisia annua* (Figure 3C). A number of previous reports have described the existence of LAP- and HAP-chemotypes of *A. annua* arising from distinct geographical locations (Lommen et al., 2006; Arsenault et al., 2010; Larson et al., 2013). It would be interesting to establish if sequence variant forms of *CYP71AV1* and differential expression of *DBR2* are generally found between these other LAP- and HAP-chemotypes.

Recent attempts to constitutively overexpress *DBR2* in transgenic *A. annua* resulted in doubling of the artemisinin concentration, which was also accompanied by a significant increase in DHAA and AA production (Yuan et al., 2015). Improvements in artemisinin concentration obtained in these experiments by Yuan et al. were significantly better than those achieved by constitutive co-expression of *CYP71AV1* and *CPR* (Shen et al., 2012), where the LAP-sequence variant of *CYP71AV1* was overexpressed in transgenic *A. annua*. Our results suggest the glandular trichome-targeted overexpression of *DBR2* specifically in the HAP-type of *CYP71AV1* might be the more efficient route to improving artemisinin production in transgenic *A. annua*.

Although arteannuin B (ArtB) was almost entirely absent from young leaf tissue of the NCV variety, as leaves matured it accumulated to become the most abundant natural product (Figure 3Avii). This observation seemed to parallel both the accumulation of artemisinin in the mature tissues of Artemis that has been noted above (Figure 3vi), as well as the recently described accumulation of arteannuin X in the mature leaves of the *cyp71av1-1* mutant of *A. annua* (Czechowski et al., 2016). The accumulation of both artemisinin and arteannuin X are considered to be the result of non-enzymatic processes, in which the 4,5-double bond of a precursor sesquiterpene undergoes spontaneous autoxidation with molecular oxygen to produce a tertiary allylic hydroperoxide. The metabolic fate of this hydroperoxide is critically dependent on the identity of the precursor—and in particular on the functionality contained elsewhere in the molecule. Thus, in the case of Artemis, the precursor is DHAA which presents a 12-carboxylic acid group (as well as saturation at the 11,13-position); whilst for the *cyp71av1-1* mutant it is amorpha-4,11-diene (A-4,11-D), which presents a 11,13-double bond (Czechowski et al., 2016). Both *in vivo* and *in vitro* experiments indicate that this difference in functionality is the basis of why DHAA-OOH (the tertiary allylic hydroperoxide from DHAA) is converted to artemisinin, whereas A-4,11-D-OOH is converted to arteannuin X (Czechowski et al., 2016).

We therefore hypothesized that the conversion of artemisinic acid (AA) to artemisitene (ArtB) in NCV may also be a non-enzymatic process, paralleling the conversion of DHAA into artemisinin in Artemis (Supplementary Figures 3A and B) and of amorpha-4,11-diene to arteannuin X in the *cyp71av1-1* mutant (Czechowski et al., 2016). The tertiary allylic hydroperoxide from artemisinic acid (AA-OOH) differs from the two foregoing examples in that it incorporates both a 12-carboxylic acid group and unsaturation at the 11,13-position. In support of this hypothesis, when a sample of AA-OOH (produced by photosensitized oxygenation of AA; and purified by HPLC) was left unattended for several weeks, it was indeed found to have been converted predominantly to ArtB (albeit at a rate that was significantly slower than for the conversion of DHAA-OOH to artemisinin). This unexpected transformation is mostly simply explained by attack of the 12-carboxylic acid group at the allylic position of the hydroperoxide, as is shown in Supplementary Figure 3A. Further studies will be required to explain why it should be that this (apparently) rather subtle modification to the 12-CO_2_H group (i.e., the introduction of 11,13-unsaturation in AA-OOH) has resulted in such a radically different pathway, as compared with DHAA-OOH.

The second most abundant product of AA-OOH conversion is *epi*-deoxyarteannuin B (EDB), which accumulates predominantly in young leaves of NCV. The EDB accumulation pattern is therefore different to DHEDB (the 11,13-saturated anaolog), where the latter's concentration rises from top to mature and dry leaves in Artemis, broadly following the accumulation pattern of artemisinin. We have proposed that the spontaneous conversions of AA into EDB and DHAA into DHEDB progress via very similar molecular mechanisms (Supplementary Figures 3C and D). Interestingly we have observed very little EDB arising from the spontaneous conversions of AA-OOH described above, which was predominantly converted to ArtB. It is known that a hydrophobic (lipophilic) environment promotes conversions of DHAA-OOH into artemisinin whereas an aqueous, acidic medium promotes DHAA-OOH conversions to DHEDB (Brown and Sy, 2004). This may also explain the very minor conversion of AA-OOH into EDB which was carried out in a hydrophobic environment (deuterated chloroform), and which promoted AA-OOH conversions to ArtB. This highlights the parallels between artemisinin and arteannuin B biogenesis shown in Supplementary Figures 3A and B. It also suggests that *in vivo* conversions of AA-OOH to EDB requires an aqueous intra-cellular environment, which might be expected to be present in young leaf trichomes, but less so in mature leaf trichomes where the sub-apical hydrophobic cavities are predominant (Ferreira and Janick, 1995), or upon cell dehydration (in dried leaf material).

Differences between the LAP and HAP chemotypes extended well beyond artemisinin-related sequiterpenes to other classes of terpenes (Figures 4 and 5, Supplemental Tables 1–3). This divergence at the level of metabolism is not that surprising given that these chemotypes also exhibit significant differences in their morphology (Figure 6). Artemis is an F1 hybrid derived from HAP parents of East Asian origin (Delabays et al., 2001) while NCV is an open-pollinated variety of European origin (personal communication with Dr. Michael Schwerdtfeger, curator of Botanical Garden at the University of Göttingen, Germany). This is consistent with the general trend for the *A. annua* varieties of European and North American origin which mostly represent the LAP chemotype and the majority of East-Asian origin varieties which represent the HAP chemotype (Wallaart et al., 2000), Details of the genetic divergence of these varieties remains a topic for further investigation that could reveal further insight into the sesquiterpene flux into different end products.

## Conclusion

This first comparative phytochemical analysis of high- (HAP) and LAP chemotypes of *A. annua* has resulted in the characterization of over 85 natural products by NMR, 26 of which have not previously been described in *A. annua*. We have also shown that the vast majority of *amorphane* sesquiterpenes are unsaturated at the 11,13-position in the LAP-chemotype as opposed to the majority of them being saturated at the 11,13-position in the HAP-chemotype. This is explained by existence of two sequence variants of *CYP71AV1* in the two investigated chemotypes and differential expression of the key branching enzyme in the artemisinin pathway, namely artemisinic aldehyde Δ 11 (13) reductase *(DBR2)*. By highlighting the main points of difference between HAP and LAP chemotypes our findings will help inform strategies for the future improvement of artemisinin production in either *A. annua* or heterologous hosts.

## Author contributions

TC planned and performed the experiments, analyzed the data, and wrote the manuscript. TL planned the UPLC-MS and GC-MS experiments, analyzed data and reviewed the manuscript. TMC planned and performed morphological plant analysis. DH performed UPLC-MS and GC-MS experiments. CW planned and performed extraction, purifications and NMR experiments and analyzed data. ME performed extraction, purifications and NMR experiments. GDB planned and performed NMR experiments, analyzed data, wrote and reviewed the manuscript. IAG planned and supervised the experiments and wrote the manuscript.

### Conflict of interest statement

The authors declare that the research was conducted in the absence of any commercial or financial relationships that could be construed as a potential conflict of interest.
