# Supplementary material for: Detailed Phytochemical Analysis of High- and Low Artemisinin-Producing Chemotypes of Artemisia annua
Source: Front Plant Sci. 2018 May 18;9:641. doi: 10.3389/fpls.2018.00641 (PMC5968107; doi:10.3389/fpls.2018.00641)
Supplement: Supplementary file 1 [file Data_Sheet_1.DOCX]

Supplementary Material

**Detailed phytochemical analysis of high- and low artemisinin-producing chemotypes of *Artemisia annua***

Tomasz Czechowski, Tony R. Larson, Theresa M. Catania, David Harvey, Cenxi Wei, Michelle Essome, Geoffrey D. Brown* and Ian A. Graham^*^

*** Correspondence:** Ian A. Graham: [ian.graham@york.ac.uk](mailto:ian.graham@york.ac.uk) and Geoffrey D. Brown: g.d.brown@reading.ac.uk


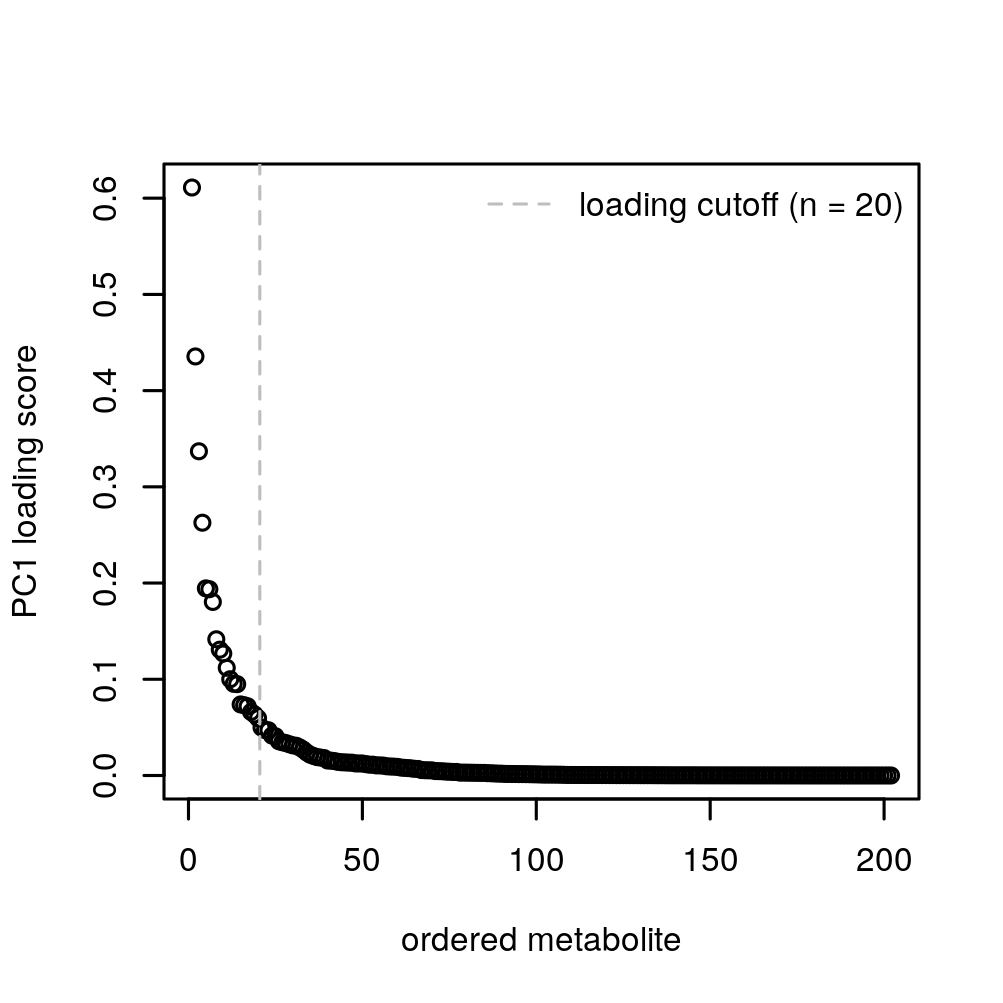

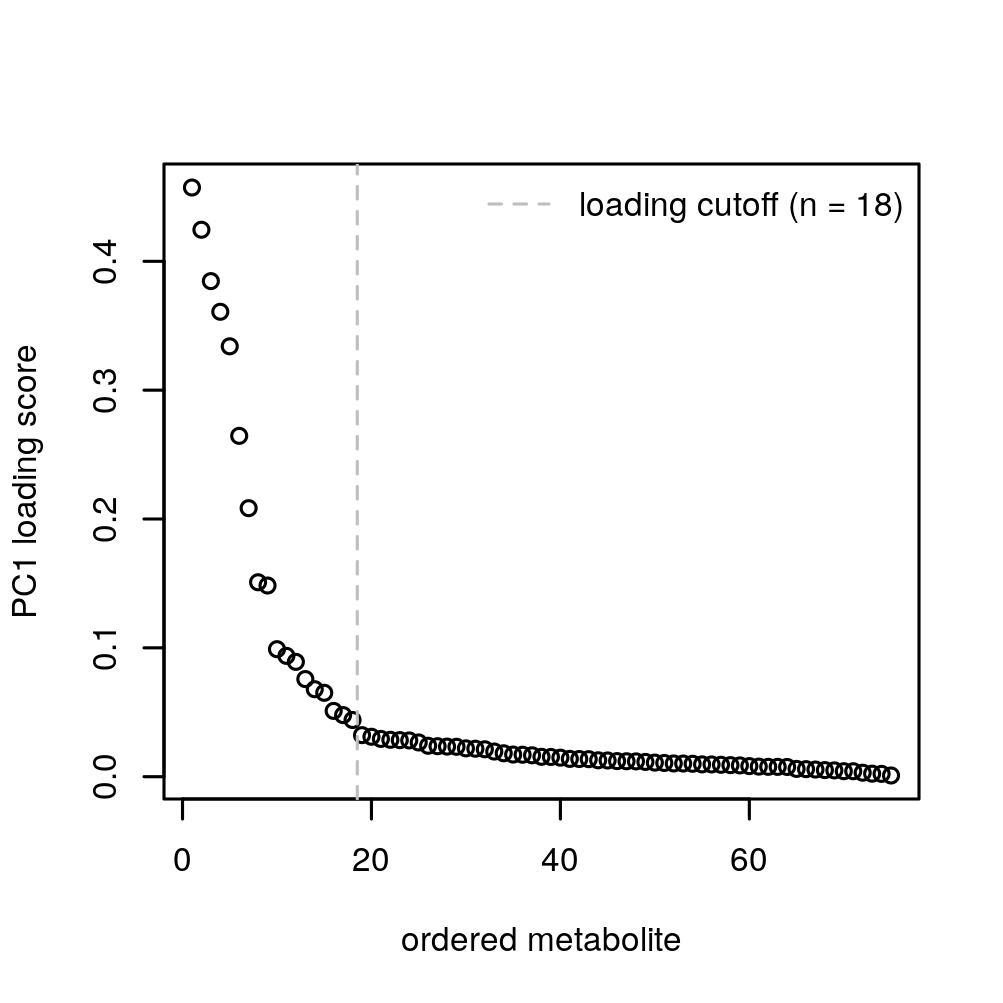


**Supplementary Figure 1. Ordered PC1 loadings plots from UPLC- and GC-MS PCA analyses.** Genotype separations in Figure 4 were best observed in the PC1 dimension. The most influential loadings were therefore chosen based on visual inspection of UPLC-MS (A) or GC-MS (B) PC1 loadings, to determine the inflexion point and selecting top n loadings to the left of this point for heatmap visualisation in Fig 5.

**
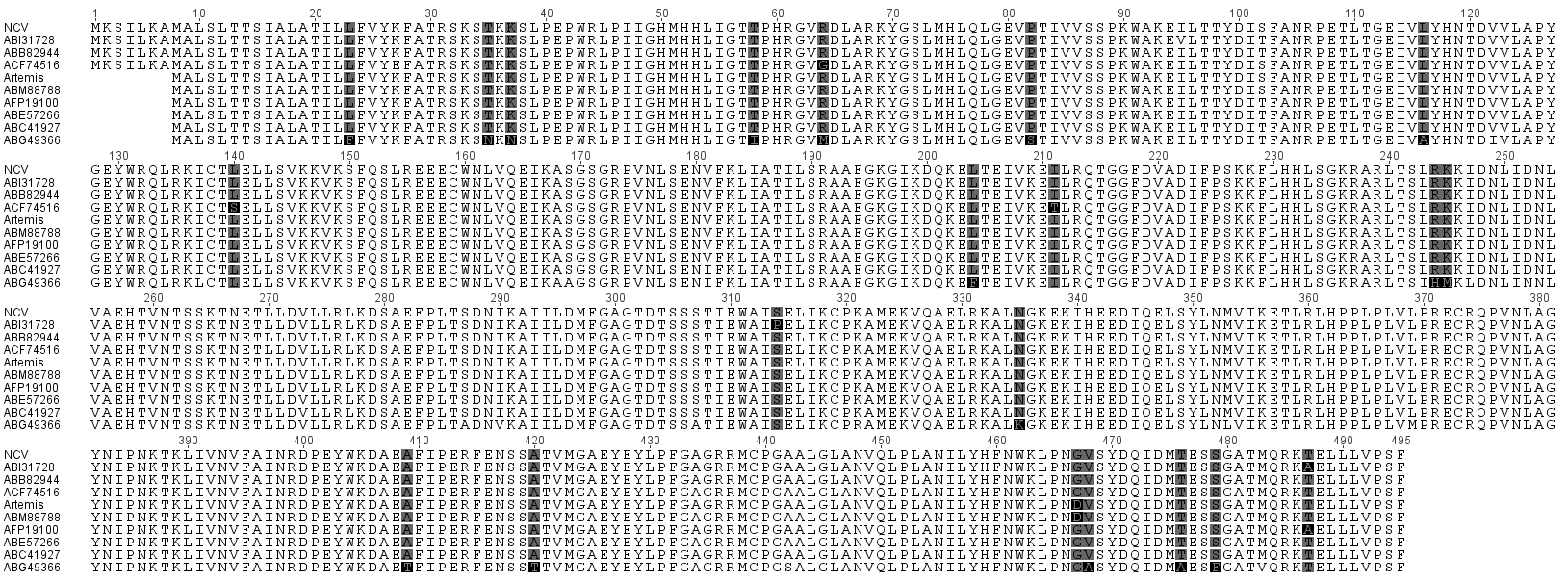
**

**Supplementary figure 2. Protein sequence divergence of CYP71AV1 for Artemis, NCV and previously published HAP and LAP varieties of *A. annua.***

Predicted protein sequence derived from consensus CDS assembled from RNAseq data for Artemis or NCV as described in materials and methods were aligned with previously published variants of CYP71AV1 deposited in GenBank. Three sequences represent LAP chemotypes: ABB82944 (Ro et al., 2006), ACF74516 (Kim et al., 1992), ABI31728 (Ting et al. 2013); and five represent HAP chemotypes: AFP19100 (Bertea et al., 2006), ABC41927 (Teoh et al., 2006), ABM88788,ABG49366 and ABE57266 (Ting et al. 2013) of *A. annua.*

**Supplementary figure 3.** Theoretical scheme showing parallels between: biogenesis of arteannuin B from AA (A) and autoxidations of DHAA to artemisinin (B); biogenesis of *epi-*doexyarteannuin B from AA (C); and dihydro-*epi*-deoxyarteannuin B from DHAA (D).

**Supplemental table 1. Comparison of natural products identified by NMR from Artemis and NCV and their relative abundance in the two varieties**

| **Natural Product** | Artemis | NCV |
| --- | --- | --- |
| 1. *Monoterpenes* |  |  |
| Camphor (**1**) | +++ |  |
| Artemisia ketone (**42**) |  | +++ |
| (*E*)-7-hydroxy-2,7-dimethylocta-2,5-dien-4-one (**43**) |  | + |
| *(E*)-7-hydroperoxy-2,7-dimethylocta-2,5-dien-4-one (**44**) |  | + |
|  |  |  |
| *ii) Sesquiterpenes* |  |  |
| *Linear* |  |  |
| *trans*-β-Farnesene (**2**) | + | +++ |
| 6,7-Epoxy*-*6,7-dihydro-β-Farnesene (**45**) |  | + |
|  |  |  |
| *Monocyclic* |  |  |
| α-Humulene (**46**) |  | + |
| γ-Humulene (**47**) |  | + |
| 6-Hydroxy-γ-humulene (6-hydroxy-1,2(*trans*),3(15),7(14)-humulatriene) (**48**) |  | + |
| β-Caryophyllene (**49**) |  | + |
| Caryophyllene oxide (**50**) |  | + |
|  |  |  |
| *Eudesmanes* |  |  |
| β-Selinene (**51**) |  | +++ |
| 5α-Hydroxy-eudesma-11-ene (**3**) | + |  |
| 5β-Hydroperoxy-eudesma-4(15),11-diene (**4**) | + |  |
| 5α-Hydroperoxy-eudesma-4(15),11-diene (**5**) | + |  |
| 4,5-*seco*-4,5-Diketo-eudesm-11-ene (**6**) | + |  |
|  |  |  |
| *Amorphanes/Cadinanes* |  |  |
| 7α-Hydroxy-amorph-4-ene (**7**) | + |  |
| Dihydroartemisinic acid (DHAA, **8**) | +++ |  |
| Artemisinic acid (AA, **9**) | + | +++ |
| 7α-Hydroxy-isoartemisinic acid (**52**) |  | + |
| Artemisinic acid methyl ester (**53**) |  | ++ |
| Arteannuin R (**54**) |  | + |
| Arteannuin S (**55**) |  | + |
| α-Epoxy-dihydroartemisinic acid (**10**) | + |  |
| α-Epoxy-artemisinic acid (**56**) |  | + |
| 4α,5α-Epoxy-6α-hydroxy-amorphan-12-oic acid (**11**) | + |  |
| 4α,5α-Epoxy-6α-hydroxy-artemisinic acid methyl ester (**57**) |  | + |
| Deoxyarteannuin B (**58**) |  | + |
| Dihydro-*epi*-deoxyarteannuin B (DHEDB, **12**) | +++ | + |
| *epi*-Deoxyarteannuin B (EDB) (**13**) | + | ++ |
| Dehydroarteannuin L (**59**) |  | + |
| Dihydroarteannuin B (**14**) | + |  |
| Arteannuin B (ArtB, **60**) |  | +++ |
| Arteannuin M (**15**) | + |  |
| Dehydroarteannuin M (**61**) |  | + |
| Arteannuin I (**16**) | ++ |  |
| Annulide (**62**) |  | ++ |
| Arteannuin J (**17**) | ++ |  |
| Isoannulide (**63**) |  | ++ |
| 11-Hydroxy-arteannuin I (**18**) | + |  |
| *epi*-11-Hydroxy arteannuin I (**64**) |  | + |
| 6α-Hydroxy-arteannuin J (**19**) | + |  |
| Arteannuin P (**20**) | + |  |
| Arteannuin H (**21**) | ++ | + |
| Artemisinic acid, 6α-peroxy ester (**65**) |  | + |
|  |  |  |
| *seco-Amorphanes* |  |  |
| Artemisinin (**22**) | +++ | + |
| Artemisitene (**66**) |  | + |
| Deoxyartemisinin (**23**) | + |  |
| Deoxyartemisitene (**67**) |  | + |
| 4,5*-seco*-4,5-Diketo-amorphan-12-oic acid (**24**) | ++ |  |
| 4,5-*seco*-4,5-Diketo-amorphan-11,13-ene-12-oic acid (**68**) |  | +++ |
| Arteannuin T (**69**) |  | + |
| Arteannuin U (**70)** |  | + |
| 4,5-*seco*-4-keto-5-carboxy-amorphan-11,13-ene-12-oic acid (**71**) |  | + |
| Arteannuin V (**72**) |  | + |
| Arteannuin W (**73**) |  | + |
|  |  |  |
| *nor-Amorphanes* |  |  |
| Arteannuin Q (**25**/**26**) | + |  |
| Arteannuin Y (**74**) |  | + |
| Norannuic acid (**75**) |  | + |
| Norannuic acid, formyl ester (**76**) |  | ++ |
| Isoarteannuin A (**77**) |  | + |
| Arteannuin Z (**78**) |  | + |
|  |  |  |
| *abeo-Amorphanes* |  |  |
| 8,7→6 *abeo* Amorphane sesquiterpene (**27**) | + |  |
|  |  |  |
| *Miscellaneous Sesquiterpenes* |  |  |
| Spathuleneol (**28**) | + |  |
| 3-(2-(2,5-Dihydrofuran-3-yl)ethyl)-2,2-dimethyl-4-methylenecyclohexan-1-one (**79**) |  | + |
|  |  |  |
| *iii) Diterpenes* |  |  |
| Phytol (**29**) | + |  |
|  |  |  |
| *iv) Triterpenes/sterols* |  |  |
| Stigmasterol (**30**) | ++ | ++ |
| α-Amyrin (**80**) |  | ++ |
| β-Amyrin (**81**) |  | ++ |
| 25,26-Dinorolean-5-en-3-ol, 9,13-dimethyl (**82**) |  | + |
|  |  |  |
| *v) Aliphatic hydrocarbons, alcohols, aldehydes and acids* |  |  |
| Trilinolein (**83**) |  | + |
| Trilinolenin (**84**) |  | + |
| Polyacetylene diol (**31**) | + |  |
| Pontica epoxide (**32**) | ++ | ++ |
| Annua diepoxide (**33**) | + |  |
|  |  |  |
| *vi) Aromatic alcohols, ketones and acids* |  |  |
| 2,4-Dihydroxy-6-methoxy acetophenone (**34**) | ++ | ++ |
| 5-Nonadecyl resorcinol-3-*O*-methyl ether (**35**) | ++ | ++ |
|  |  |  |
| *vii) Phenylpropanoids* |  |  |
| Eugenol (**36**) | + |  |
| Coumarin (**37**) | +++ | +++ |
| Scopoletin (**38**) | +++ | +++ |
| Isofraxidin (**39**) | + | + |
|  |  |  |
| *viii) Flavonoids* |  |  |
| Chrysosplenetin (**40**) | +++ | +++ |
| Casticin (**41**) | ++ | ++ |
| 5-Hydroxy-3,6,7-trimethoxy-2-(4’-methoxyphenyl)-4*H*-chromen-4-one (**85**) |  | + |

Table legend: +++ - highly abundant; ++ - medium abundance; + - low abundance

**Supplemental table 2 Levels of 75 UPLC-MS detected metabolites for three different leaf types extracted from HAP (Artemis) and LAP (NCV) chemotypes of *A. annua***

Submitted as a separate Excel spreadsheet

**Supplemental table 3 Levels of 202 GC-MS detected metabolites for two different leaf types extracted from HAP (Artemis) and LAP (NCV) chemotypes of *A. annua***

Submitted as a separate Excel spreadsheet

**Supplementary List 1.** Natural products isolated and characterized from the Artemis variety of *A. annua* by the preparative HPLC/ 1D- and 2D-NMR approach.

1. *Monoterpenes*

Camphor (**1**) (known) in CDCl_3_ solution

NMR data for camphor (**1**) in CDCl_3_ solution

| **Position** | **δ_H_ (ppm)** | **δ_C_ (ppm)** | **HMBC** | **^1^H-^1^H COSY** |
| --- | --- | --- | --- | --- |
| 1 | - | 57.7 | 2.08 (H-4), 1.68 (H-6), 1.40 (H-6), 0.96 (H-8), 0.91 (H-10), 0.84 (H-9) | - |
| 2 | - | 218.8 | 2.35 (H-3), 2.08 (H-4), 1.84 (H-3), 0.91 (H-10) | - |
| 3 | 2.35 [1H], ddd, *J* = 18, 4, 4 Hz  1.84 [1H], d, *J* = 18 Hz | 43.3 | - | 2.08 (H-4) |
| 4 | 2.08 [1H], dd, *J*= 4, 4 Hz | 43.1 | 0.96 (H-8), 0.84 (H-9) | 2.35 (H-3), 1.95 (H-5) |
| 5 | 2.05 [1H], m  1.95 [1H], ddddd, *J* = 12, 12, 4, 4, 4 Hz | 27.1 | 1.84 (H-3) | 2.08 (H-4), 1.68 (H-6),1.40 (H-6) |
| 6 | 1.68 [1H], ddd, *J* = 12, 12, 4 Hz  1.40 [1H], m | 29.9 | 2.05 (H-5), 1.95 (H-5), 0.91 (H-10) | 1.95 (H-4) |
| 7 | - | 46.8 | 1.84 (H-3), 1.40 (H-6), 0.96 (H-8), 0.91 (H-10), 0.84 (H-9) | - |
| 8 | 0.96 [3H] s | 19.1 | - | - |
| 9 | 0.84 [3H] s | 19.8 | - | - |
| 10 | 0.91 [3H] s | 9.3 | - | - |

*Camphor* (**1**). Purification from fraction D by isocratic column chromatography (6% EtOAc/hexane), yielding fraction D5 - 8 (204 mg). Colourless oil. IR ν (cm^-1^): 2959 (m), 2873 (w), 1741 (s), 1448 (w). Direct injection-HRMS (*m/z*): 153.1270, Δ = -2.6 ppm for M+H^+^ (calc. 153.1274 for C_10_H_17_O); 175.1090, Δ = -2.1 ppm for M+Na^+^ (calc. 175.1093 for C_10_H_16_ONa); 327.2292, Δ= -0.7 ppm for M_2_ + Na^+^ (calc. 327.2295 for C_20_H_32_O_2_Na).

1. *Sesquiterpenes*

*Linear sesquiterpenes*

*trans*-β-Farnesene (**2**) (known)

NMR data for *trans*-β-farnesene (**2**) in CDCl_3_ solution

| **Position** | **δ_H_ (ppm)** | **δ_C_ (ppm)** | **HMBC** | **^1^H-^1^H COSY** |
| --- | --- | --- | --- | --- |
| 1 | 5.06 [1H], d, *J* = 11 Hz | 113.0 | 6.38 (H-2) | 6.38 (H-2) |
|  | 5.25 [1H], d, *J* = 18 Hz |  |  |  |
| 2 | 6.38 [1H], dd, *J* = 18, 11 Hz | 139.0 | 5.25 (H-1), 2.23 (H-4), 5.00 (H-13) | 5.25 (H-1), 5.06 (H-1) |
| 3 | - | 146.1 | 6.38 (H-2), 5.25 (H-1), 5.06 (H-1), 5.02 (H-13), 2.23 (H-4), 2.19 (H-5) | - |
| 4 | 2.23 [2H], m | 31.4 | 2.19 (H-5), 5.00 (H-13), 5.02 (H-13),  6.38 (H-2) | 2.19 (H-5) |
| 5 | 2.19 [2H], m | 26.6 | 2.23 (H-4) | 5.16 (H-6), 2.23 (H-4) |
| 6 | 5.16 [1H], t, *J* = 7 Hz | 124.0 | 2.19 (H-5), 2.23 (H-4), 1.99 (H-8), 1.60 (H-14) | 2.19 (H-5) |
| 7 | - | 135.4 | 2.19 (H-5), 1.99 (H-8), 1.60 (H-14) | - |
| 8 | 1.99 [2H], m | 39.7 | 2.07 (H-9), 1.60 (H-14) | 2.07 (H-9) |
| 9 | 2.07 [2H], m | 26.7 | 1.99 (H-8) | 5.10 (H-10),1.99 (H-8) |
| 10 | 5.10 [1H], t, *J* = 7 Hz | 124.4 | 1.68 (H-12), 1.61 (H-15) | 2.07 (H-9) |
| 11 | - | 131.3 | 1.68 (H-12), 1.61 (H-15) | - |
| 12 | 1.68 [3H], s | 25.7 | 1.61 (H-15) | - |
| 13 | 5.02 [1H], br s | 115.7 | 6.38 (H-2), 2.23 (H-4) | - |
|  | 5.00 [1H], s |  |  |  |
| 14 | 1.60 [3H], br s | 16.0 | 5.16 (H-6), 1.99 (H-8) | - |
| 15 | 1.61 [3H], s | 17.7 | 5.10 (H-10), 1.68 (H-12), | - |

*trans-β-Farnesene* (**2**). Purification from the NCV extract of *A. annua* by gradient column chromatography (10% EtOAc/hexane), yielding fraction B; followed by isocratic column chromatography (hexane), yielding fraction B6 - 12. Colourless oil.

*Eudesmane sesquiterpenes*

5α-Hydroxy-eudesma-11-ene (**3**) (previously reported as 4αH-eudesm-5α-ol^1^ – NMR assignments at positions 1- and 8- have been corrected in the Table below)

NMR data for 5α-hydroxy-eudesma-11-ene (**3**) in CDCl_3_ solution

| **Position** | **δ_H_ (ppm)** | **δ_C_ (ppm)** | **HMBC** | **^1^H-^1^H COSY** |
| --- | --- | --- | --- | --- |
| 1 | 1.59 [1H] m  1.05 [1H] m | 35.0 | 1.09 (H-14) | - |
| 2 | 1.42 [1H] br d *J* = 13 Hz  1.76 [1H] m | 17.1 | - | 1.35 (H-3) |
| 3 | 1.35 [1H] m  2.06 [1H] ddd*, J* = 12, 12, 5, 5 Hz | 28.1 | 1.05 (H-15) | 1.76 (H-2) |
| 4 | 1.63 [1H] m | 41.2 | 1.05 (H-15) | 1.05 (H-15) |
| 5 | - | 75.9 | 1.09 (H-14), 1.05 (H-15) | - |
| 6 | 1.22 [1H] m  1.86 [1H] dd *J* = 13, 12 Hz | 37.6 | - | 2.53 (H-7) |
| 7 | 2.53 [1H] ddd, *J* = 12, 12, 4, 4 Hz | 40.0 | 4.73 (H-12), 4.71 (H-12) | 1.86 (H-6), 1.56 (H-8), 1.53 (H-8), 1.22 (H-6) |
| 8 | 1.56 [1H] m  1.53 [1H] m | 26.2 | - | 2.53 (H-7) |
| 9 | 1.77 [1H] m  1.00 [1H] m | 38.1 | 1.09 (H-14) | - |
| 10 | - | 36.8 | 1.09 (H-14) | - |
| 11 | - | 150.8 | 1.76 (H-13), 1.86 (H-6) | - |
| 12 | 4.73 [1H] s  4.71 [1H] s | 108.2 | 1.76 (H-13) | - |
| 13 | 1.76 [3H] s | 21.2 | 4.73 (H-12), 4.71 (H-12) | - |
| 14 | 1.09 [3H] s | 21.8 | - | - |
| 15 | 1.05 [3H] d *J* = 7 Hz | 16.8 | - | 1.63 (H-4) |

*5α-hydroxy-eudesma-11-ene* (**3**) was purified from fraction D by isocratic column chromatography (6% EtOAc/hexane), yielding fraction D4; followed by HPLC (6% EtOAc/hexane), yielding D4 HPLC fraction 12 (*R_t_* 14.2 mins). Colourless oil.

1. A. H. Elmi; M. H. Farah; E. Fattorusso; S. Magno; L. Mayol “Volatile mono- and sesquiterpenoids from *Kleinia pendula*.” *Phytochemistry* **26**(11) 3069-71 (1987).

5β-Hydroperoxy-eudesma-4(15),11-diene (**4**) (novel)

NMR data for 5β-hydroperoxy-eudesma-4(15),11-diene (**4**) in CDCl_3_ solution

| **Position** | **δ_H_ (ppm)** | **δ_C_ (ppm)** | **HMBC** | **^1^H-^1^H COSY** |
| --- | --- | --- | --- | --- |
| 1 | 1.18 [1H], m | 36.7 | 1.56 (H-2), 1.06 (H-14) | **-** |
|  | 1.83 [1H], m |  |  |  |
| 2 | 1.56 [1H], m | 21.9 | - | 2.38 (H-3), 2.22 (H-3) |
|  | 1.62 [1H], m |  |  |  |
| 3 | 2.22 [1H], m | 33.1 | 5.29 (H-15), 5.07 (H-15) | 1.62 (H-2), 1.56 (H-2) |
|  | 2.38 [1H], ddd, *J* = 13, 13, 5 Hz |  |  |  |
| 4 | - | 144.7 | 5.07 (H-15), 2.38 (H-3), 1.73 (H-6) | 5.29 (H-15) |
| 5 | 6.77 (5-OOH) [1H], s | 87.3 | 5.29 (H-15), 5.07 (H-15), 1.73 (H-6), 1.06 (H-14) | - |
| 6 | 1.73 [1H], dd, *J* = 14, 13 Hz | 31.3 | - | 2.26 (H-7) |
|  | 2.41 [1H], dd, *J* = 13, 4 Hz |  |  |  |
| 7 | 2.26 [1H], m | 42.4 | 4.75 (H-13), 1.76 (H-12), 1.73 (H-6) | 2.41 (H-6), 1.73 (H-6) |
| 8 | 1.59 [2H], m | 26.6 | - | 1.84 (H-9) |
| 9 | 1.05 [1H], m | 34.6 | 1.06 (H-14) | 1.59 (H-8) |
|  | 1.84 [1H], |  |  |  |
| 10 | - | 39.1 | 1.18 (H-1), 1.06 (H-14) | - |
| 11 | - | 149.5 | 4.75 (H-13) | - |
| 12 | 1.76 [3H], s | 20.6 | 4.75 (H-13), 4.72 (H-13) | 4.75 (H-13), 4.72 (H-13) |
| 13 | 4.72 [1H], s | 108.6 | 1.76 (H-12) | 1.76 (H-12) |
|  | 4.75 [1H], s |  |  |  |
| 14 | 1.06 [3H], s | 22.5 | 1.83 (H-1) | - |
| 15 | 5.07 [1H], s | 115.9 | - | 2.38 (H-3) |
|  | 5.29 [1H], s |  |  |  |

*5β-hydroperoxy-eudesma-4(15),11-diene (****4****)* was purified from a gradient column fraction eluting with 10% EtOAC/hexane, followed by HPLC (10% EtOAc/hexane/1% AcOH), yielding HPLC fraction 11 (*R_t_* 10.9 mins). Colourless oil.

5α-Hydroperoxy-eudesma-4(15), 11-diene (**5**) (known^1^)

NMR data for 5α-hydroperoxy-eudesma-4(15), 11-diene (**5**) in CDCl_3_ solution

| **Position** | **δ_H_ (ppm)** | **δ_C_ (ppm)** | **HMBC** | **^1^H-^1^H COSY** |
| --- | --- | --- | --- | --- |
| 1 | 1.18 [1H], dd, *J* = 12, 12 Hz | 34.4 | 1.63 (H-2), 0.93 (H-14) | - |
|  | 1.78 [1H], s |  |  |  |
| 2 | 1.63 [2H], m | 22.4 | - | 2.49 (H-3), 2.19 (H-3) |
| 3 | 2.19 [1H], br | 32.1 | 2.49 (H-3) | 5.07 (H-15), 4.79 (H-15), 1.63 (H-2) |
|  | 2.49 [1H], s |  |  |  |
| 4 | - | 148.2 | - | - |
| 5 | 6.80 [1H], s (5-OOH) | 87.3 | 5.07 (H-15), 0.93 (H-14) | - |
| 6 | 1.50 [1H], dd, *J* = 14, 13 Hz | 29.0 | - | 2.45 (H-7) |
|  | 2.11 [1H], br |  |  |  |
| 7 | 2.45 [1H], m | 39.6 | 4.75 (H-13), 1.78 (H-12), 1.50 (H-6) | 1.50 (H-6) |
| 8 | 1.51 [1H], m | 26.1 | - | - |
|  | 1.61 [1H], t, *J* = 12 Hz |  |  | - |
| 9 | 1.03 [1H], br | 34.6 | 0.93 (H-14) | - |
|  | 1.81 [1H], m |  |  | - |
| 10 | - | 38.8 | 1.18 (H-1), 0.93 (H-14) | - |
| 11 | - | 150.4 | 4.75 (H-13), 1.78 (H-12) | - |
| 12 | 1.78 [3H], s | 21.1 | - | 4.77 (H-13), 4.75 (H-13), |
| 13 | 4.75 [1H], s  4.77 [1H], s | 108.4 | 1.78 (H-12) | 4.77 (H-13), 4.75 (H-13), 1.18 (H-1) |
| 14 | 0.93 [3H], s | 21.2 | - | - |
| 15 | 4.79 [1H], s | 111.8 | - | 2.49 (H-3) |
|  | 5.07 [1H], s |  |  |  |

*5α-hydroperoxy-eudesma-4(15),11-diene (****5****)* was purified from a gradient column fraction eluting with 10% EtOAC/hexane, followed by HPLC (10% EtOAc/hexane/1% AcOH), yielding HPLC fraction 10 (*R_t_* 10.5 mins). Colourless oil.

1.G.D. Brown; G.-Y. Liang; L.-K. Sy “Terpenoids from the seeds of *Artemisia annua*” *Phytochemistry* **64** 303-323 (2003).

4,5-*seco*-4,5-Diketo-eudesm-11-ene (**6**) (previously reported as 2α-(5-oxopentyl)-2β-methyl-5β-isopropenylcyclohexanone^1^ – NMR assignments at 3-, 6- and 7- corrected in the Table below)

NMR data for the 4,5-*seco*-4,5-Diketo-eudesm-11-ene (**6**) in CDCl_3_ solution

| **Position** | **δ_H_ (ppm)** | **δ_C_ (ppm)** | **HMBC** | **^1^H-^1^H COSY** |
| --- | --- | --- | --- | --- |
| 1 | 1.46 [2H], m | 37.2 | 2.45 (H-3), 1.55 (H-2), 1.14 (H-14) | 1.55 (H-2) |
| 2 | 1.55 [1H], m  1.52 [1H], m | 18.2 | 2.45 (H-4) | 2.45 (H-3), 1.46 (H-1) |
| 3 | 2.45 [2H], m | 44.3 | 2.14 (H-15), 1.52 (H-3) | 1.55 (H-2), 1.52 (H-2) |
| 4 | - | 209.0 | 2.45 (H-4), 2.14 (H-15), 1.55 (H-3), 1.52 (H-3) | - |
| 5 | - | 215.3 | 2.51 (H-7), 1.14 (H-14) | - |
| 6 | 2.35 [1H], d, *J* = 15 Hz  2.51 [1H], dd, *J* = 15, 13 Hz | 43.4 | 1.83 (H-9) | - |
| 7 | 2.33 [1H], m | 45.8 | 4.78 (H-12), 4.72 (H-12), 2.51 (H-7), 1.76 (H-10) | - |
| 8 | 1.75 [1H], m  1.83 [1H], m | 26.1 | 2.51 (H-7), 2.35 (H-7) | - |
| 9 | 1.55 [1H], m  1.76 [1H], m | 36.3 | 1.14 (H-14) | - |
| 10 | - | 47.4 | 1.46 (H-2), 1.14 (H-14) | - |
| 11 | - | 147.5 | 1.74 (H-13), 2.51 (H-7), 2.35 (H-7) | - |
| 12 | 4.72 [1H], br s  4.78 [1H], br s | 110.0 | 1.74 (H-13) | 1.74 (H-13) |
| 13 | 1.74 [3H], s | 20.8 | 4.78 (H-12), 4.72 (H-12) | 4.78 (H-12), 4.72 (H-12) |
| 14 | 1.14 [3H], s | 23.2 | - | - |
| 15 | 2.14 [3H], s | 29.9 | 2.45 (H-4) | - |

4,5-seco*-4,5-Diketo-eudesm11-ene* (**6**) was purified from fraction O by isocratic column chromatography (15% EtOAc/hexane), yielding fraction O6 - 8; followed by HPLC (15% EtOAc/hexane/1% AcOH), yielding O6 - 8 HPLC fraction 11 (*R_t_* 15 mins). Colorless oil. IR ν (cm^-1^): 2960 (sh), 2929 (m), 2857 (w), 1739 (m), 1705 (s), 1458 (m). LC-HRMS (*m/z*): 237.1849, Δ = -0.2 ppm for M+H^+^ (calc. 237.1849 for C_15_H_25_O_2_); 259.1668, Δ = -0.2 ppm for M+Na^+^ (calc. 259.1669 for C_15_H_24_O_2_Na); R*_t_* 9.60 mins.

1. S. Ohira; T. Hasegawa; K.-I. Hayashi; T. Hoshino; D. Takaoka; H. Nozaki. “Sesquiterpenoids from *Cyperus rotundus*”. *Phytochemistry* **47**(8) 1577–1581 (1998).

*Cadinane/Amorphane sesquiterpenes*

7α-hydroxy-amorph-4-ene (**7**) (previously reported as cadin-4-en-7-ol^1^ – NMR assignments at positions 12- and 14- have been corrected in the Table below)

NMR data for 7α-hydroxy-amorph-4-ene (**7**) in CDCl_3_ solution

| **Position** | **δ_H_ (ppm)** | **δ_C_ (ppm)** | **HMBC** | **^1^H-^1^H COSY** |
| --- | --- | --- | --- | --- |
| 1 | 1.66 [1H], m | 36.7 | 0.89 (H-14) | - |
| 2 | 1.47 [1H], m | 25.3 | - | - |
|  | 1.96 [1H], m |  |  |  |
| 3 | 1.80 [1H], m | 26.3 | 1.63 (H-15) | - |
|  | 1.88 [1H], m |  |  |  |
| 4 | - | 135.7 | 1.63 (H-15) | - |
| 5 | 5.18 [1H], s | 120.3 | 2.33 (H-6), 1.63 (H-15) | - |
| 6 | 2.33 [1H], br | 44.1 | - | - |
| 7 | - | 74.9 | 0.93 (H-12), 0.90 (H-13) | - |
| 8 | 1.13 [1H], s | 31.8 | - | 1.43 (H-9), 1.26 (H-9) |
|  | 1.66 [1H], d, *J* = 13 Hz |  |  |  |
| 9 | 1.26 [1H], s | 30.2 | 0.89 (H-14) | 1.66 (H-8), 1.13 (H-8) |
|  | 1.43 [1H], m |  |  |  |
| 10 | 1.42 [1H], m | 27.2 | 0.89 (H-14) | 0.89 (H-14) |
| 11 | 1.86 [1H], m | 32.9 | 0.93 (H-12), 0.90 (H-13), | 0.93 (H-12), 0.90 (H-13) |
| 12 | 0.93 [3H], d, *J* = 7 Hz | 16.1 | - | 1.86 (H-11) |
| 13 | 0.90 [3H], d, *J* = 7 Hz | 15.7 | - | 1.86 (H-11) |
| 14 | 0.89 [3H], d, *J* = 6 Hz | 19.5 | - | 1.42 (H-10) |
| 15 | 1.63 [3H], s | 24.0 | 5.18 (H-5) | - |

*7α-hydroxy-amorph-4-ene (****7****)* was purified from a gradient column fraction eluting with 10% EtOAC/hexane, followed by HPLC (10% EtOAc/hexane/1% AcOH), yielding HPLC fraction 12 (*R_t_* 12.5 mins).

1. J. F. Sanz; V. Garcia-Lliso; J. A. Marco; J. Vales-Xirau. “A cadinane derivative from *Artemisia crithmifolia*”. *Phytochemistry* **30**(12) 4167-4168 (1991).

Dihydroartemisinic acid (**8**) (known^1^)

NMR data for dihydroartemisinic acid (**8**) in CDCl_3_ solution

| **Position** | **δ_H_ (ppm)** | **δ_C_ (ppm)** | **HMBC** | **^1^H-^1^H COSY** |
| --- | --- | --- | --- | --- |
| 1 | 1.26 [1H], m | 41.8 | 5.12 (H-5), 0.87 (H-14) | - |
| 2 | 1.55[1H], m  1.95 [1H], dddd, *J* = 14, 3 ,3 ,3 Hz | 25.8 | - | - |
| 3 | 1.81[1H], dd, *J* = 15, 7 Hz  1.92 [1H], br d*, J* = 15 Hz | 26.7 | 5.12 (H-5) | - |
| 4 | - | 136.2 | 1.92 (H-3), 1.81 (H-3), 1.63 (H-15) | - |
| 5 | 5.12 [1H], br s | 119.4 | 1.92 (H-3), 1.81 (H-3), 1.63 (H-15) | 1.63 (H-7) |
| 6 | 2.51 [1H], br s | 36.4 | 5.12 (H-5) | 1.63 (H-7) |
| 7 | 1.63 [1H], m | 43.6 | 5.12 (H-5), 2.49 (H-11), 1.19 (H-13) | 2.51 (H-6) |
| 8 | 1.11 [1H], dddd, *J* =12, 12, 12, 3 Hz  1.40 [1H], m | 27.4 | - | 1.63 (H-7), 0.96 (H-9) |
| 9 | 0.96 [1H], dddd, *J* = 12, 12, 12, 3 Hz  1.61 [1H], m | 35.3 | 0.87 (H-14) | 1.40 (H-8), 1.11 (H-8) |
| 10 | 1.44 [1H], m | 27.7 | 0.87 (H-14) | 0.87 (H-14) |
| 11 | 2.49 [1H], dq, *J* = 12, 7 Hz | 42.3 | 1.19 (H-13) | 1.19 (H-13) |
| 12 | - | 184.3 | 2.49 (H-11) | - |
| 13 | 1.19 [3H], d, *J* = 7 Hz | 15.1 | 2.49 (H-11) | 2.49 (H-11) |
| 14 | 0.87 [3H], d, *J* = 7 Hz | 19.7 | - | 1.44 (H-10) |
| 15 | 1.63 [3H], s | 23.8 | 5.12 (H-5) | - |

*Dihydroartemisinic acid* (**8**) was purified from fraction K by HPLC (5% EtOAc/hexane/1% AcOH), yielding K HPLC fraction 8 (*R_t_* 11.8 mins). Colorless oil.

1. L.-K. Sy and G. D. Brown, “A novel endoperoxide and related sesquiterpenes from *Artemisia annua* which are possibly derived from allylic hydroperoxides”, *Tetrahedron* **54** 4345-4356 (1998).

Artemisinic acid (**9**) (known^1^)

NMR data for artemisinic acid (**9**) in CDCl_3_ solution

| **Position** | **δ_H_ (ppm)** | **δ_C_ (ppm)** | **HMBC** | **^1^H-^1^H COSY** |
| --- | --- | --- | --- | --- |
| 1 | 1.42 [1H], m | 41.4 | 4.98 (H-5) | - |
| 2 | 1.55 [1H], m  1.93 [1H], m | 25.6 | - | - |
| 3 | 1.76 [1H], m  1.87 [1H], m | 26.4 | 4.98 (H-5), 1.60 (H-15) | - |
| 4 | - | 134.9 | 1.93 (H-2), 1.76 (H-3), 1.60 (H-15) | - |
| 5 | 4.98 [1H], s | 120.2 | 1.60 (H-15) | - |
| 6 | 2.60 [1H], br | 37.9 | 4.98 (H-5) | 2.69 (H-7) |
| 7 | 2.69 [1H], d, *J* = 12 Hz | 42.1 | 6.44 (H-13, 5.55 (H-13) | 2.60 (H-6), 1.39 (H-8) |
| 8 | 1.36 [1H], m  1.39 [1H], m | 26.0 | - | 2.69 (H-7) |
| 9 | 1.06 [1H], m  1.71 [1H], dddd, *J* =12, 3, 3, 3 Hz | 35.2 | 1.00 (H-14) | - |
| 10 | 1.42 [1H], m | 27.6 | 1.00 (H-14) | 1.00 (H-14) |
| 11 | - | 142.7 | 6.45 (H-13) | - |
| 12 | - | 172.3 | 6.45 (H-13), 5.55 (H-13) | - |
| 13 | 5.55 [1H], s  6.44 [1H], s | 126.5 | - | - |
| 14 | 1.00 [3H], d, *J* = 6 Hz | 19.8 | - | 1.42 (H-10) |
| 15 | 1.60 [3H], s | 23.8 | 4.98 (H-5) | - |

*Artemisinic acid* (**9**). Purification from fraction K by HPLC (5% EtOAc/hexane/1% AcOH), yielding K HPLC fraction 7 (*R_t_* 11.0 mins). (Artemisinic acid (**9**) was also found in Fraction J, HPLC 9). Colorless Oil. IR ν (cm^-1^): 3600-3000 (br), 2925 (s), 2869 (m), 2854 (m), 1758 (s), 1670 (w), 1455 (m). LC-HRMS (*m/z*): 235.1693, Δ = 0.0 ppm for M+H^+^ (calc. 235.1693 for C_15_H_23_O_2_); R*_t_* 1.12 mins. HRAPCIMS (*m/z*): 235.1693, Δ = 0.0 ppm for [M+H]^+^ (calc. 235.1693 for C_15_H_23_O_2_).

1. G.D. Brown and L.-K. Sy "*In vivo* transformations of artemisinic acid in *Artemisia annua* plants" *Tetrahedron* **63** 9548-9566 (2007).

α-Epoxy-dihydroartemisinic acid (**10**) (known^1^)

NMR data for α-epoxy-dihydroartemisinic acid (**10**) in CDCl_3_ solution

| **Position** | **δ_H_ (ppm)** | **δ_C_ (ppm)** | **HMBC** | **^1^H-^1^H COSY** |
| --- | --- | --- | --- | --- |
| 1 | 1.06 [1H], dddd, *J* = 12, 12, 12, 3 Hz | 40.2 | 2.64 (H-5), 0.86 (H-14) | - |
| 2 | 1.30 [1H], m  1.64 [1H], m | 22.3 | - | - |
| 3 | 1.66 [1H], m  1.82 [1H], m | 24.8 | 1.31 (H-15) | - |
| 4 | - | 57.7 | 2.64 (H-5), 1.82 (H-3), 1.31 (H-15) | - |
| 5 | 2.64 [1H], s | 58.7 | 2.03 (H-6), 1.31 (H-15) | 2.03 (H-6) |
| 6 | 2.03 [1H], br | 38.2 | 2.64 (H-5) | 2.64 (H-5), 1.73 (H-7) |
| 7 | 1.73 [1H], dddd *J* = 12, 12, 6, 6 Hz | 42.8 | 2.64 (H-5) | 2.03 (H-6), 1.10 (H-8) |
| 8 | 1.10 [1H], dddd, *J* = 12, 12, 12, 3 Hz  1.63 [1H], m | 28.7 | - | 1.73 (H-7) |
| 9 | 1.00 [1H], dddd, *J* = 12, 12, 12, 3 Hz  1.68 [1H], m | 34.8 | 0.86 (H-14) | - |
| 10 | 1.26 [1H], m | 29.6 | 0.86 (H-14) | 0.86 (H-14) |
| 11 | 2.60 [1H], dq, *J* = 12, 7 Hz | 42.1 | 1.29 (H-13) | 1.73 (H-7), 1.29 (H-13) |
| 12 | - | 181.8 | 2.60 (H-11), 1.29 (H-13) | - |
| 13 | 1.29 [3H], d, *J* = 7 Hz | 15.4 | - | 2.60 (H-11) |
| 14 | 0.86 [3H], d, *J* = 6 Hz | 19.1 | - | 1.26 (H-10) |
| 15 | 1.31 [3H], s | 23.6 | - | - |

*α-Epoxy-dihydroartemisinic acid* (**10**) was purified from fraction K by HPLC (5% EtOAc/hexane/1% AcOH), yielding HPLC fraction 30 (*R_t_* 39.8 mins). Colorless oil. IR ν (cm^-1^): 3600-2900 (v br), 2922 (s), 2852 (m), 1713 (m), 1673 (m), 1566 (m), 1458 (m). LC-HRMS (*m/z*): 253.1798, Δ = 0.0 ppm for M+H^+^ (calc. 253.1798 for C_15_H_25_O_3_); 235.1692, Δ = -0.1 ppm for M+H^+^-H_2_O (calc. 235.1693 for C_15_H_23_O_2_); 275.1617, Δ = -0.1 ppm for M+Na^+^ (calc. 275.1618 for C_15_H_24_O_3_Na); R*_t_* 7.49 mins.

1. G.D. Brown; G.-Y. Liang; L.-K. Sy “Terpenoids from the seeds of *Artemisia annua*” *Phytochemistry* **64** 303-323 (2003).

4α,5α-Epoxy-6α-hydroxy-amorphan-12-oic acid (**11**) (known^1^)

NMR data for 4α,5α-epoxy-6α-hydroxy-amorphan-12-oic acid (**11**) in CDCl_3_ solution

| **Position** | **δ_H_ (ppm)** | **δ_C_ (ppm)** | **HMBC** | **^1^H-^1^H COSY** |
| --- | --- | --- | --- | --- |
| 1 | 1.00 [1H], br d, *J* = 12 Hz | 48.7 | 3.22 (H-5), 0.88 (H-14) | 1.42 (H-2), 1.23 (H-10) |
| 2 | 1.42 [1H], m  1.61 [1H], br, dd, *J* = 13, 13 Hz | 15.2 | 1.85 (H-3) | - |
| 3 | 1.65 [1H], m  1.85 [1H], dd, *J* = 14, 5 Hz | 24.5 | 1.36 (H-15) | - |
| 4 | - | 60.9 | 3.22 (H-5), 1.36 (H-15) | - |
| 5 | 3.22 [1H], s | 61.0 | 1.36 (H-15) | - |
| 6 | - | 70.7 | 3.22 (H-5), 3.06 (H-11) | - |
| 7 | 1.79 [1H], m | 50.1 | 3.22 (H-5), 3.06 (H-11), 1.33 (H-13) | 3.06 (H-11) |
| 8 | 1.27 [1H], m  1.80 [1H], br, d *J* = 12 Hz | 26.3 | 3.06 (H-11) | - |
| 9 | 1.08 [1H], ddd, *J* = 13, 12, 12 Hz  1.69 [1H], dd, *J* = 13, 4 Hz | 34.6 | 0.88 (H-14) | - |
| 10 | 1.23 [1H], m | 30.7 | 0.88 (H-14) | 0.88 (H-14) |
| 11 | 3.06 [1H], m | 39.6 | 1.79 (H-7), 1.33 (H-13) | 1.79 (H-7), 1.33 (H-13) |
| 12 | - | 179.6 | 3.06 (H-11), 1.80 (H-8), 1.79 (H-7), 1.33 (H-13) | - |
| 13 | 1.33 [3H], d, *J* = 7 Hz | 18.9 | 3.06 (H-11) | 3.06 (H-11) |
| 14 | 0.88 [3H], d *J* = 7 Hz | 19.1 | - | 1.23 (H-10) |
| 15 | 1.36 [3H], s | 23.5 | 3.22 (H-5) | - |

*4α,5α-Epoxy-6α-hydroxy-amorphan-12-oic acid* (**11**). Purification from fraction T by isocratic column chromatography (50% EtOAc/hexane), yielding fraction T3; followed by HPLC (15% EtOAc/hexane/1% AcOH), yielding T3 HPLC fraction 16 (*R_t_* 28 mins). Colourless oil. IR ν (cm^-1^): 3445 (br), 3013 (w), 2953 (m), 2925 (m), 2879 (m), 2853 (m), 1707 (s), 1454 (s). LC-HRMS (*m/z*): 269.1747, Δ = 0.0 ppm for M+H^+^ (calc. 269.1747 for C_15_H_25_O_4_); 251.1641, Δ = -0.2 ppm for M+H^+^-H_2_O (calc. 251.1642 for C_15_H_23_O_3_); 291.1566, Δ = -0.1 ppm for M+Na^+^ (calc. 291.1567 for C_15_H_24_O_4_Na); R*_t_* 5.52 mins.

1. G. D. Brown; G.-Y. Liang; L.-K. Sy “Terpenoids from the seeds of *Artemisia annua*” *Phytochemistry* **64** 303-323 (2003).

Dihydro-*epi*-deoxyarteannuin B (**12**) (known^1-3^)

NMR data for dihydro-*epi*-deoxyarteannuin B (**12**) in CDCl_3_ solution

| **Position** | **δ_H_ (ppm)** | **δ_C_ (ppm)** | **HMBC** | **^1^H-^1^H COSY** |
| --- | --- | --- | --- | --- |
| 1 | 1.20 [1H], s | 46.5 | 1.89 (H-2), 1.68 (H-2), 0.94 (H-14) | - |
| 2 | 1.68 [1H], m | 21.0 | 2.10 (H-3) | 2.10 (H-3) |
|  | 1.89 [1H], m |  |  |  |
| 3 | 2.06 [1H], m | 30.8 | 1.70 (H-15) | 1.68 (H-2) |
|  | 2.10 [1H], m |  |  |  |
| 4 | - | 142.4 | 5.64 (H-5), 2.10 (H-3),1.70 (H-15) | - |
| 5 | 5.64 [1H], s | 121.7 | 2.10 (H-3), 1.70 (H-15) | - |
| 6 | - | 83.4 | 5.64 (H-5), 2.11 (H-7), 1.20 (H-1) | - |
| 7 | 2.11 [1H], m | 42.8 | 3.15 (H-11), 1.18 (H-8), 1.15 (H-13) | - |
| 8 | 1.18 [1H], m | 23.8 | 2.11 (H-7), 1.05 (H-9) | 1.05 (H-9) |
|  | 1.74 [1H], m |  |  |  |
| 9 | 1.05 [1H], m | 32.4 | 1.18 (H-8), 0.94 (H-14) | 1.18 (H-8) |
|  | 1.66 [1H], m |  |  |  |
| 10 | 1.57 [1H], m | 29.6 | 1.20 (H-1), 0.94 (H-14) | 0.94 (H-14) |
| 11 | 3.15 [1H], dq, *J* = 7, 7 Hz | 39.7 | 2.11 (H-7), 1.15 (H-13) | 1.15 (H-13) |
| 12 | - | 179.6 | 3.15 (H-11), 1.15 (H-13) | - |
| 13 | 1.15 [3H], d, *J* = 7 Hz | 9.4 | - | 3.15 (H-11) |
| 14 | 0.94 [3H], d, *J* = 7 Hz | 19.6 | - | 1.57 (H-10) |
| 15 | 1.70 [3H], s | 23.4 | - | - |

*Dihydro-*epi*-deoxyarteannuin B* (**12**) was purified from fraction I; followed by isocratic column chromatography (5% EtOAc/hexane), yielding fraction I1 - 2 (101 mg). Colourless oil. IR ν (cm^-1^): 2986 (sh), 2925 (s), 2873 (m), 2853 (m), 1765 (s), 1669 (w), 1455 (m). LC-HRMS (*m/z*): 235.1693, Δ = 0.0 ppm for M+H^+^ (calc. 235.1693 for C_15_H_23_O_2_); R*_t_* 11.53 mins. ESIMS (*m/z*): 235.1693, ∆ = 0.3 ppm for [M + H^+^] (calc. for C_15_H_23_O_2_); R_t_ 7.67 mins. HRAPCIMS (*m/z*): 235.1694, Δ = -0.5 ppm for [M+H]^+^ (calc. 235.1693 for C_15_H_23_O_2_).

1. G.D. Brown. "Two new compounds from *Artemisia annua*". *J. Nat. Prod.* **55** 1756-1770 (1992).

2. L.-K. Sy; G. D. Brown, “A novel endoperoxide and related sesquiterpenes from *Artemisia annua* which are possibly derived from allylic hydroperoxides”, *Tetrahedron* **54** 4345-4356 (1998).

3. L.-K. Sy; G.D. Brown "Deoxyarteannuin B, dihydro-deoxyarteannuin B and *trans*-5-hydroxy-2-isopropenyl-5-methylhex-3-en-1-ol from *Artemisia annua*". *Phytochemistry* **58** 1159-1166 (2001).

*epi*-Deoxyarteannuin B (**13**) (known^1,2^)

NMR data for *epi*-deoxyarteannuin B (**13**) in CDCl_3_ solution

| **Position** | **δ_H_ (ppm)** | **δ_C_ (ppm)** | **HMBC** | **^1^H-^1^H COSY** |
| --- | --- | --- | --- | --- |
| 1 | 1.25 [1H], m | 44.2 | 0.97 (H-14) | 1.68 (H-2) |
| 2 | 1.68 [1H], m | 21.6 | - | 2.10 (H-3), 2.03 (H-3), 1.25 (H-1) |
|  | 1.86 [1H], m |  |  |  |
| 3 | 2.03 [1H], m | 31.0 | 1.67 (H-15) | 1.86 (H-2), 1.68 (H-2) |
|  | 2.10 [1H], m |  |  |  |
| 4 | - | 141.3 | 1.67 (H-15) | - |
| 5 | 5.28 [1H], br s | 123.8 | 1.67 (H-15) | - |
| 6 | - | 83.4 | - | - |
| 7 | 2.71 [1H], m | 44.9 | 6.17 (H-13), 5.56 (H-13) | 6.17 (H-13), 5.56 (H-13), 1.91 (H-8), 1.37 (H-8) |
| 8 | 1.37 [1H], m | 28.5 | - | 2.71 (H-7), 1.66 (H-9) |
|  | 1.91 [1H], m |  |  |  |
| 9 | 1.11 [1H], m | 29.9 | 0.97 (H-14) | 1.91 (H-8) |
|  | 1.66 [1H], m |  |  |  |
| 10 | 1.56 [1H], m | 28.4 | 0.97 (H-14) | 0.97 (H-14) |
| 11 | - | 142.9 | 6.17 (H-13) | - |
| 12 | - | 170.5 | 6.17 (H-13), 5.56 (H-13) | - |
| 13 | 5.56 [1H], s | 120.7 | - | 2.71 (H-7) |
|  | 6.17 [1H], s |  |  |  |
| 14 | 0.97 [3H], d, *J* = 7 Hz | 20.0 | - | 1.56 (H-10) |
| 15 | 1.67 [3H], s | 23.2 | - | 5.28 (H-5) |

epi*-Deoxyarteannuin B* (**13**). Purification from fraction I by isocratic column chromatography (5% EtOAc/hexane), yielding fraction I3 - 5; followed by HPLC (5% EtOAc/hexane), yielding I3 – 5, HPLC fraction 5 (*R_t_* 15.8 mins). Colourless oil. Direct injection-HRMS (*m/z*): 233.1533, Δ = -1.3 ppm for M+H^+^ (calc. 233.1536 for C_15_H_21_O_2_); 255.1353, Δ = -1.2 ppm for M+Na^+^ (calc. 255.1356 for C_15_H_20_O_2_Na). ESI-MS (*m/z*): 233.1536, ∆ = 0.3 ppm for [M + H^+^] (calc. for C_15_H_21_O_2_); R_t_ 7.63 mins. HRAPCIMS (*m/z*): 233.1537, Δ = -0.4 ppm for [M+H]^+^ (calc. 233.1536 for C_15_H_21_O_2_).

1. G. D. Brown. "Two new compounds from *Artemisia annua*". *J. Nat. Prod.* **55** 1756-1770 (1992).

2. L.-K. Sy; G. D. Brown "Deoxyarteannuin B, dihydro-deoxyarteannuin B and *trans*-5-hydroxy-2-isopropenyl-5-methylhex-3-en-1-ol from *Artemisia annua*". *Phytochemistry* **58** 1159-1166 (2001).

Dihydroarteannuin B (**14**) (known^1^)

NMR data for dihydroarteannuin B (**14**) in CDCl_3_ solution

| **Position** | **δ_H_ (ppm)** | **δ_C_ (ppm)** | **HMBC** | **^1^H-^1^H COSY** |
| --- | --- | --- | --- | --- |
| 1 | 1.34 [1H], m | 45.7 | 3.03 (H-5), 0.96 (H-14) | - |
| 2 | 1.49 [1H], m  1.76 [1H], ddd, *J* = 12, 12, 4 Hz | 16.2 | - | - |
| 3 | 1.69 [1H], ddd, *J* =15, 14, 8 Hz  1.94 [1H], dd, *J* = 14, 6 Hz | 24.1 | 1.36 (H-15) | - |
| 4 | - | 58.3 | 3.03 (H-5) | - |
| 5 | 3.03 [1H], s | 59.5 | 1.36 (H-15) | - |
| 6 | - | 83.1 | 3.03 (H-5), 2.78 (H-11) | - |
| 7 | 2.25 [1H], m | 50.2 | 3.03 (H-5), 2.78 (H-11), 1.38 (H-13) | 2.78 (H-11) |
| 8 | 1.52 [1H], m  1.82 [1H], br d, *J* =16 Hz | 21.7 | - | - |
| 9 | 1.23 [1H], m  1.89 [1H], br d, *J* =13 Hz | 34.8 | 0.96 (H-14) | - |
| 10 | 1.49 [1H], m | 30.5 | 0.96 (H-14) | 0.96 (H-14) |
| 11 | 2.78 [1H], dq, *J* = 8, 7 Hz | 38.6 | 1.38 (H-13) | 2.25 (H-7), 1.38 (H-13) |
| 12 | - | 179.8 | 1.38 (H-13) | - |
| 13 | 1.38 [3H], d, *J* = 7 Hz | 12.6 | - | 2.78 (H-11) |
| 14 | 0.96 [3H], d, *J* = 8 H_Z_ | 18.4 | - | 1.49 (H-10) |
| 15 | 1.36 [3H], s | 23.1 | - | - |

*Dihydroarteannuin B* (**14**). Purification from fraction T by isocratic column chromatography (50% EtOAc/hexane), yielding fraction T3; followed by HPLC (15% EtOAc/hexane/1% AcOH), yielding T3 HPLC fraction 19 (*R_t_* 42 mins). Colourless oil. IR ν (cm^-1^): 2918 (s), 2850 (m), 1733 (m), 1462 (w). LC-HRMS (*m/z*): 251.1645, Δ = 1.1 ppm for M+H^+^ (calc. 251.1642 for C_15_H_23_O_3_); R*_t_* 7.26 mins.

1. L.-K. Sy; G. D. Brown, “A novel endoperoxide and related sesquiterpenes from *Artemisia annua* which are possibly derived from allylic hydroperoxides”, *Tetrahedron* **54** 4345-4356 (1998).

Arteannuin M (**15**) (known^1,2^)

NMR data for arteannuin M (**15**) in CDCl_3_ solution

| **Position** | **δ_H_ (ppm)** | **δ_C_ (ppm)** |
| --- | --- | --- |
| 1 | 1.52 [1H], s | 41.7 |
| 2 | 1.32 [1H], m | 22.1 |
|  | 1.78 [1H], m |  |
| 3 | 1.72 [1H] | 34.3 |
|  | 1.62 [1H], m |  |
| 4 | - | 72.6 |
| 5 | 3.45 [1H], s | 74.2 |
| 6 | - | 86.2 |
| 7 | 2.65 [1H], m | 39.1 |
| 8 | 1.73 [1H], m | 23.9 |
|  | 1.13 [1H], m |  |
| 9 | 1.07 [1H], m | 32.3 |
|  | 1.61 [1H], m |  |
| 10 | 1.36 [1H], m | 29.9 |
| 11 | 3.09 [1H], dq, *J* = 7, 7 Hz | 38.8 |
| 12 | - | 179.1 |
| 13 | 1.13 [3H], d, *J* = 7 Hz | 9.3 |
| 14 | 0.93 [3H], d, *J* = 6 Hz | 20.1 |
| 15 | 1.39 [3H], s | 26.6 |

*Arteannuin M (****15****)*. HREIMS (*m/z*): 250.1565 (M^+^ - H_2_O) C_15_H_22_O_4_ requires 250.1569 (12); 222 (18); 195 (100)

1. L.-K. Sy; G. D. Brown, “A novel endoperoxide and related sesquiterpenes from *Artemisia annua* which are possibly derived from allylic hydroperoxides”, *Tetrahedron* **54** 4345-4356 (1998).
2. L.-K. Sy, K.-K.Cheung, N.-Y. Zhu; G. D. Brown “Structure elucidation of arteannuin O, a novel cadinane diol from *Artemisia annua* and the synthesis of arteannuins K, L, M and O*” Tetrahedron* **57** 8481-8493 (2001)

Arteannuin I (**16**) (known^1^)

NMR data for arteannuin I (**16**) in CDCl_3_ solution

| **Posi-tion** | **δ_H_ (ppm)** | **δ_C_ (ppm)** | **HMBC** | **^1^H-^1^H COSY** |
| --- | --- | --- | --- | --- |
| 1 | 1.56 [1H], d, *J* = 12, 5, 5 Hz | 43.7 | 0.91 (H-14), 1.47 (H-2), 1.98 (H-6) | - |
| 2 | 1.47 [1H], dddd, *J* = 13, 12, 5, 5 Hz | 28.6 | 1.56 (H-1), 2.25 (H-3) | 2.15 (H-3) |
|  | 2.00 [1H], ddddd, *J* = 13, 2, 2, 2, 2 Hz |  |  |  |
| 3 | 2.15 [1H], ddd, *J* = 13, 13, 4 Hz | 29.5 | 1.47 (H-2), 4.82 (H-15) | 1.47 (H-2) |
|  | 2.25 [1H], ddd, *J* = 13, 5, 2 Hz |  |  |  |
| 4 | - | 146.2 | 5.06 (H-15), 4.82 (H-15), 4.97 (H-5), 2.25 (H-3), 2.15 (H-3) | - |
| 5 | 4.97 [1H], d, *J* = 9 Hz | 76.8 | 5.06 (H-15), 4.82 (H-15) 1.98 (H-6) | 1.98 (H-6) |
| 6 | 1.98 [1H], ddd, *J* = 12, 4, 4 Hz | 45.5 | 1.91 (H-7) | 4.97 (H-5) |
| 7 | 1.91 [1H], m | 39.9 | 4.97 (H-5), 2.70 (H-11), 1.80 (H-8), 1.24 (H-13) | - |
| 8 | 1.75 [1H], m | 22.7 | 2.70 (H-11), 1.91 (H-7), 1.04 (H-9) | 1.04 (H-9) |
|  | 1.80 [1H], dddd, *J* = 13, 3, 3, 3 Hz |  |  |  |
| 9 | 1.04 [1H], dddd, *J* = 13, 13, 13, 4 Hz | 35.0 | 1.80 (H-8), 1.76 (H-10), 0.91 (H-14) | 1.80 (H-8) |
|  | 1.89 [1H], m |  |  |  |
| 10 | 1.76 [1H], m | 28.1 | 1.76 (H-10), 0.91 (H-14) | 0.91 (H-14) |
| 11 | 2.70 [1H], dq, *J* = 7, 7 Hz | 40.5 | 2.70 (H-11), 1.24 (H-13) | 1.24 (H-13) |
| 12 | - | 174.6 | 2.70 (H-11), 1.24 (H-13) | - |
| 13 | 1.24 [3H], d, *J* = 7 Hz | 13.3 | 2.70 (H-11) | 2.70 (H-11) |
| 14 | 0.91 [3H], d, *J* = 6 Hz | 20.1 | - | 1.76 (H-10) |
| 15 | 4.82 [1H], br s | 105.1 | 4.97 (H-5), 2.25 (H-3), 2.15 (H-3) | - |
|  | 5.06 [1H], br s |  |  |  |

*Arteannuin I* (**16**). Purification of fraction I by isocratic column chromatography (5% EtOAc/hexane), yielding fraction I3 - 5; followed by HPLC (5% EtOAc/hexane), yielding I3-5 HPLC fraction 7 (*R_t_* 18.4 mins; also containing a little of dihydro-*epi-*deoxyarteannuin B (**12**)). (Arteannuin I (**16**) was also obtained from T3 HPLC 8 (15% EtOAc/hexane/1% AcOH; *R_t_* 14 mins)). Colourless oil. IR ν (cm^-1^): 2983 (w), 2943 (m), 2934 (m), 2918 (m), 2873 (m), 2856 (m), 1737 (s), 1660 (w), 1442 (m). LC-HRMS (*m/z*): 235.1690, Δ = -1.0 ppm for M+H^+^ (calc. 235.1693 for C_15_H_23_O_2_); 469.3307, Δ = -1.1 ppm for M_2_+ H^+^ (calc. 469.3312 for C_30_H_45_O_4_); R*_t_* 11.60 mins.

1. L.-K. Sy and G. D. Brown, “A novel endoperoxide and related sesquiterpenes from *Artemisia annua* which are possibly derived from allylic hydroperoxides”, *Tetrahedron* **54** 4345-4356 (1998).

Arteannuin J (**17**) (known^1^)

NMR data for arteannuin J (**17**) in CDCl_3_ solution

| **Position** | **δ_H_ (ppm)** | **δ_C_ (ppm)** | **HMBC** | **^1^H-^1^H COSY** |
| --- | --- | --- | --- | --- |
| 1 | 1.53 [1H], m | 41.7 | 2.25 (H-2), 1.42 (H-10), 0.88 (H-14) | 2.25 (H-2) |
| 2 | 2.16 [1H], m | 27.2 | 5.39 (H-3) | 5.39 (H-3), 1.53 (H-1) |
|  | 2.25 [1H], m |  |  |  |
| 3 | 5.39 [1H], br s | 122.9 | 5.39 (H-3), 2.25 (H-2), 1.79 (H-15), 1.53 (H-1) | 2.25 (H-2), 2.16 (H-2) |
| 4 | - | 131.8 | 5.39 (H-3), 4.96 (H-5), 1.79 (H-15) | - |
| 5 | 4.96 [1H], d, *J* = 9 Hz | 75.4 | 1.79 (H-15) | 2.15 (H-6) |
| 6 | 2.15 [1H], dd, *J* = 9, 9 Hz | 40.4 | 4.96 (H-5), 1.95 (H-7) | 4.96 (H-5) |
| 7 | 1.95 [1H], m | 38.8 | 2.76 (H-11), 1.40 (H-8), 1.24 (H-13) | 2.76 (H-11), 1.37 (H-8) |
| 8 | 1.37 [1H], dddd, *J* = 12, 12, 12, 3 Hz | 23.1 | 2.76 (H-11), 1.95 (H-7) 1.40 (H-8) | 1.95 (H-7), 1.89 (H-9), 1.04 (H-9) |
|  | 1.75 [1H], m |  |  |  |
| 9 | 1.04 [1H], dddd, *J* = 15, 13, 5, 3 Hz | 34.9 | 1.89 (H-9), 1.42 (H-10), 1.40 (H-8), 0.88 (H-14) | 1.75 (H-8), 1.57 (H-10) |
|  | 1.89 [1H], m |  |  |  |
| 10 | 1.42 [1H], m | 29.8 | 1.04 (H-9), 0.88 (H-14) | 1.04 (H-9), 0.88 (H-14) |
| 11 | 2.76 [1H], dq, *J* = 7, 7  Hz | 40.7 | 1.95 (H-7), 1.24 (H-13) | 1.95 (H-7), 1.24 (H-13) |
| 12 | - | 175.7 | 2.76 (H-11), 1.24 (H-13) | - |
| 13 | 1.24 [3H], d*, J* = 7 Hz | 13.3 | 2.76 (H-11) | 2.76 (H-11) |
| 14 | 0.88 [3H], d, *J* = 7 Hz | 20.2 | - | 1.57 (H-10) |
| 15 | 1.79 [3H], s | 18.3 | - | - |

*Arteannuin J* (**17**) was purified from fraction I by isocratic column chromatography (5% EtOAc/hexane), yielding fraction I3 - 5; followed by HPLC (5% EtOAc/hexane), yielding I3 - 5 HPLC fraction 8 (*R_t_* 19.0 mins; also containing a little arteannuin I (**16**). Colourless oil. IR ν (cm^-1^): 2923 (m), 2871 (m), 2855 (m), 1735 (m), 1445 (m). LC-HRMS (*m/z*): 235.1691, Δ = -0.5 ppm for M+H^+^ (calc. 235.1693 for C_15_H_23_O_2_); 469.33101, Δ = -0.6 ppm for M_2_ +H^+^ (calc. 469.3312 for C_30_H_45_O_4_); R*_t_* 11.89 mins.

1. L.-K. Sy; G. D. Brown, “A novel endoperoxide and related sesquiterpenes from *Artemisia annua* which are possibly derived from allylic hydroperoxides”, *Tetrahedron* **54** 4345-4356 (1998).

11-Hydroxy-arteannuin I (**18**) (novel)

NMR data for 11-hydroxy-arteannuin I (**18**) in CDCl_3_ solution

| **Position** | **δ_H_ (ppm)** | **δ_C_ (ppm)** | **HMBC** | **^1^H-^1^H COSY** |
| --- | --- | --- | --- | --- |
| 1 | 1.65 [1H], ddd, *J* = 10, 5, 5 Hz | 41.5 | 0.92 (H-14) | - |
| 2 | 1.47 [1H], dddd, *J* = 12, 10 ,3, 3 Hz 2.00 [1H], m | 29.0 | - | - |
| 3 | 2.20 [2H], m | 29.6 | 4.90 (H-15), 4.73 (H-15) | - |
| 4 | - | 148.1 | 4.90 (H-15), 4.47 (H-5) | - |
| 5 | 4.47 [1H], d, *J* = 12 Hz | 79.4 | 4.90 (H-15), 4.73 (H-15) | 2.03 (H-6) |
| 6 | 2.03 [1H], m | 49.1 | 4.47 (H-5) | 4.47 (H-5), 2.23 (H-7) |
| 7 | 2.23 [1H], m | 46.5 | 1.54 (H-13) | 2.03 (H-6) |
| 8 | 1.37 [1H], dddd, *J*= 13, 12, 12, 3 Hz  1.83 [1H], dddd, *J*= 13, 6, 3, 3 Hz | 24.0 | - | 1.00 (H-9) |
| 9 | 1.74 [1H], dddd, *J* = 14, 3, 3, 3 Hz  1.00 [1H], dddd, *J* =14, 12, 12, 3 Hz | 34.4 | 0.92 (H-14) | 1.37 (H-8) |
| 10 | 1.56 [1H], m | 28.7 | 0.92 (H-14) | 0.92 (H-14) |
| 11 | - | 87.7 | 1.54 (H-13) | - |
| 12 | - | 176.5 | 1.54 (H-13) | - |
| 13 | 1.54 [3H], s | 26.4 | - | - |
| 14 | 0.92 [3H], d, *J* = 7 Hz | 20.0 | - | 1.56 (H-10) |
| 15 | 4.73 [1H], s  4.90 [1H], s | 101.9 | 4.47 (H-5) | - |

*11-Hydroxy-arteannuin I* (**18**). Purification from fraction T by isocratic column chromatography (50% EtOAc/hexane), yielding fraction T3; followed by HPLC (15% EtOAc/hexane/1% AcOH), yielding T3 HPLC fraction 9 (*R_t_* 15 mins). Colourless oil. IR ν (cm^-1^): 3401 (br), 2918 (s), 2850 (m), 1718 (m), 1666 (w), 1462 (m). LC-HRMS (*m/z*): 251.1643, Δ = 0.6 ppm for M+H^+^ (calc. 251.1642 for C_15_H_23_O_3_); R*_t_* 9.39 mins.

6α-Hydroxy-arteannuin J (**19**) (novel)

NMR data for 6α-hydroxy-arteannuin J (**19**) in CDCl_3_ solution

| **Positions** | **δ_H_ (ppm)** | **δ_C_ (ppm)** | **HMBC** | **^1^H-^1^H COSY** |
| --- | --- | --- | --- | --- |
| 1 | 1.48 [1H], dd, *J* =12, 6 Hz | 48.1 | 0.94 (H-14) | 2.18 (H-2) |
| 2 | 2.18[H], ddddd, *J* = 18, 2, 2, 2, 2 Hz  2.44 [1H], dddd, *J* = 18, 9, 3, 3 Hz | 24.2 | - | 5.53 (H-3), 1.48 (H-1) |
| 3 | 5.53 [1H], br s | 123.4 | 1.82 (H-15),1.48 (H-1) | 2.18 (H-2) |
| 4 | - | 129.7 | 1.82 (H-15) | - |
| 5 | 5.03 [1H], br s | 76.0 | 1.94 (H-7) | - |
| 6 | - | 70.1 | 1.94 (H-7) | - |
| 7 | 1.94 [1H], ddd, *J* = 17, 5, 5 Hz | 43.8 | 1.22 (H-13) | - |
| 8 | 1.63 [1H], m  1.91 [1H], dddd, *J* = 14, 5, 4, 4 Hz | 24.8 | - | - |
| 9 | 1.09 [1H], m  1.85 [1H], dddd, *J* = 14, 4, 4, 4 Hz | 34.4 | 0.94 (H-14) | - |
| 10 | 1.34 [1H], m | 32.9 | 0.94 (H-14) | 0.94 (H-14) |
| 11 | 3.35 [1H], dq *J* = 7, 7 Hz | 35.7 | 1.22 (H-13) | 1.22 (H-13) |
| 12 | - | 175.1 | 1.22 (H-13) | - |
| 13 | 1.22 [3H], d, *J* =7 Hz | 12.9 | - | 3.35 (H-11) |
| 14 | 0.94 [3H], d, *J* = 7 Hz | 20.6 | - | 1.34 (H-10) |
| 15 | 1.82 [3H], s | 18.7 | - | - |

*6α-Hydroxy-arteannuin J* (**19**) was purified from fraction T by isocratic column chromatography (50% EtOAc/hexane), yielding fraction T3; followed by HPLC (15% EtOAc/hexane/1% AcOH), yielding T3 HPLC fraction 20 (*R_t_*  44 mins). Colourless oil.

Arteannuin P (**20**) (novel)

NMR data for arteannuin P (**20**) in CDCl_3_ solution

| **Position** | **δ_H_ (ppm)** | **δ_C_ (ppm)** | **HMBC** | **^1^H-^1^H COSY** |
| --- | --- | --- | --- | --- |
| 1 | 1.91 [1H], ddd, *J* = 11, 6, 5 Hz | 44.9 | 6.07 (H-2), 5.60 (H-3), 5.23 (H-5), 2.25 (H-6), 0.99 (H-14) | 6.07 (H-2) |
| 2 | 6.07 [1H], dd *J* = 12, 6 H_Z_ | 133.2 | 1.91 (H-1) | 5.60 (H-3), 1.91 (H-1) |
| 3 | 5.60 [1H], d, *J* = 12 Hz | 130.3 | 1.91 (H-1), 1.29 (H-15) | 6.07 (H-2) |
| 4 | 7.75 [1H], br s (4-OOH) | 85.5 | 5.23 (H-5), 2.25 (H-6), 1.29 (H-15), 6.07 (H-2), | - |
| 5 | 5.23 [1H], d *J* = 13 Hz | 75.0 | 1.29, 5.60, 2.25, 1.91 | 2.25 (H-6) |
| 6 | 2.25 [1H], ddd, *J* = 13, 4, 4 Hz | 37.8 | 6.07 (H-2), 5.23 (H-5), 1.94 (H-7) | 5.23 (H-5) |
| 7 | 1.94 [1H], dddd, *J* = 13, 4, 4, 4 Hz | 39.6 | 2.67 (H-11), 1.26 (H-13) | - |
| 8 | 1.33 [1H], m  1.80 [1H], dddd, *J* = 12, 3, 3, 3 Hz | 22.4 | 2.67 (H-11), 2.25 (H-6) | - |
| 9 | 1.06 [1H], m  1.87 [1H], dddd *J* = 13, 3, 3, 3 Hz | 34.7 | 0.99 (H-14) | - |
| 10 | 1.57 [1H], m | 34.4 | 2.25 (H-6), 1.91 (H-1), 0.99 (H-14) | 0.99 (H-14) |
| 11 | 2.67 [1H], dq *J* = 6, 6 Hz | 40.1 | 2.25 (H-6), 1.26 (H-13) | 1.26 (H-13) |
| 12 | - | 173.6 | 2.67 (H-11) ,1.26 (H-13) | - |
| 13 | 1.26 [3H], d *J*= 6 Hz | 13.4 | 2.67 (H-11) | 2.67 (H-11) |
| 14 | 0.99 [3H], d *J* = 7 Hz | 20.0 | 1.91 (H-1) | 1.57 (H-10) |
| 15 | 1.29 [3H], s | 18.1 | 5.23 (H-5) | - |
| -OOH | 7.73 br s | - | - | - |

*Arteannuin P* (**20**) wad purified from fraction J by HPLC (10% EtOAc/hexane), yielding HPLC fraction 23 (R_t_ 23 mins).

Arteannuin H (**21**) (known^1,2^)

NMR data for arteannuin H (**21**) in CDCl_3_ solution

| **Position** | **δ_H_ (ppm)** | **δ_C_ (ppm)** | **HMBC** | **^1^H-^1^H COSY** |
| --- | --- | --- | --- | --- |
| 1 | 1.54 [1H], br d, *J* = 12 Hz | 45.2 | 0.88 (H-14), 1.38 (H-2) | 2.08 (H-6), 1.65 (H-10), 1.38 (H-2) |
| 2 | 1.38 [1H], dddd, *J* = 14, 14, 4, 4 Hz | 28.6 | - | 2.28 (H-3), 2.19 (H-3), 1.54 (H-1) |
|  | 1.95 [1H], dddd, *J* = 14, 4, 4, 2 Hz |  |  |  |
| 3 | 2.19 [1H], ddd, *J* = 14, 14, 4 Hz | 30.7 | 4.91 (H-15), 4.84 (H-15), 1.38 (H-2) | 1.95 (H-2), 1.38 (H-2) |
|  | 2.28 [1H], dddd, *J* = 14, 14, 4, 4 Hz |  |  |  |
| 4 | - | 145.3 | 5.03 (H-5), 4.91 (H-15), 2.28 (H-3), 2.19 (H-3), 2.08 (H-6), 1.95 (H-2), 1.38 (H-2) | - |
| 5 | 5.03 [1H], d, *J* = 12 Hz | 84.8 | 4.91 (H-15), 4.84 (H-15), 2.28 (H-3), 2.19 (H-3), 2.08 (H-6), 1.74 (H-7), 1.54 (H-1) | 2.08 (H-6) |
| 6 | 2.08 [1H], ddd, *J* = 12, 4, 4 Hz | 50.9 | 3.53 (H-11) | 5.03 (H-5), 1.74 (H-7), 1.54 (H-1) |
| 7 | 1.74 [1H], dddd, *J* = 13, 4, 4, 2 Hz | 43.2 | 3.52 (H-11), 2.08 (H-6), 1.22 (H-13) | 2.08 (H-6) |
| 8 | 1.27 [1H], m | 22.7 | 1.06 (H-9), 1.88 (H-9), 3.52  (H-11) | 1.88 (H-9), 1.77 (H-8), 1.06 (H-9) |
|  | 1.77 [1H], dddd, *J* = 13, 3, 3, 3 Hz |  |  |  |
| 9 | 1.06 [1H], dddd, *J* = 13, 13, 13, 4 Hz | 35.5 | 0.88 (H-14) | 1.77 (H-8), 1.65 (H-10), 1.27 (H-8) |
|  | 1.88 [1H], m |  |  |  |
| 10 | 1.65 [1H], m | 28.6 | 0.88 (H-14) | 1.54 (H-1), 1.06 (H-9), 0.88 (H-14) |
| 11 | 3.52 [1H], dq, *J* = 2, 7 Hz | 40.3 | 1.22 (H-13) | 1.22 (H-13) |
| 12 | - | 180.2 | 3.52 (H-11), 1.74 (H-7), 1.22 (H-13) | - |
| 13 | 1.22 [3H], d, *J* = 7 Hz | 15.6 | 3.52 (H-11) 1.74 (H-7) | 3.52 (H-11) |
| 14 | 0.88 [3H], d, *J* = 6 Hz | 19.7 | - | 1.65 (H-10) |
| 15 | 4.84 [1H], ddd, *J* = 1, 1, 1 Hz  4.91 [1H], ddd, *J* = 1, 1, 1 Hz | 106.6 | 5.03 (H-5), 2.28 (H-3), 2.19 (H-3) | - |

*Arteannuin H* (**21**) was purified from fraction O by isocratic column chromatography (15% EtOAc/hexane), yielding fraction O1 - 2 (50 mg). (Arteannuin H (**21**) also found in O3 - 5 HPLC 4 (8% EtOAc/hexane/1% AcOH); and fraction J - HPLC 7). Colourless oil. IR ν (cm^-1^): 2918 (s), 2871 (m), 2850 (m), 1707 (s), 1456 (m). LC-HRMS (*m/z*): 235.1693, Δ = 0.0 ppm for M+H^+^ (calc. 235.1693 for C_15_H_23_O_2_); R*_t_* 7.94 mins.

1. L.-K. Sy; G. D. Brown, “A novel endoperoxide and related sesquiterpenes from *Artemisia annua* which are possibly derived from allylic hydroperoxides”. *Tetrahedron* **54** 4345-4356 (1998).
2. L.-K. Sy, K.-S. Ngo; G.D. Brown "Biomimetic synthesis of arteannuin H and the 3,2-rearrangement of allylic hydropeoxides". *Tetrahedron* **55** 15127-15140 (1999).

*seco-Amorphanes*

Artemisinin (**22**) (known)

NMR data for artemisinin (**22**) in CDCl_3_ solution

| **Position** | **δ_H_ (ppm)** | **δ_C_ (ppm)** | **HMBC** | **^1^H-^1^H COSY** |
| --- | --- | --- | --- | --- |
| 1 | 1.38 [1H], m | 50.0 | 2.05 (H-3), 1.99 (H-2), 0.99 (H-14) | - |
| 2 | 1.48 [1H], m | 24.8 | - | 2.05 (H-3) |
|  | 1.99 [1H], ddddd, *J* = 14, 3, 3, 3, 3 Hz |  |  |  |
| 3 | 2.05 [1H], m | 35.9 | 1.44 (H-15) | 1.48 (H-2) |
|  | 2.42 [1H], dd, *J* = 14, 13 Hz |  |  |  |
| 4 | - | 105.5 | 5.86 (H-5), 2.42 (H-3),1.44 (H-15) | - |
| 5 | 5.86 [1H], s | 93.7 | 1.44 (H-15), 1.38 (H-1) | - |
| 6 | - | 79.5 | 5.86 (H-5), 1.38 (H-1) | - |
| 7 | 1.76 [1H], d, *J* = 13 Hz | 44.9 | 1.21 (H-13) | 3.39 (H-11) |
| 8 | 1.07 [1H], m | 23.4 | - | - |
|  | 1.87 [1H], m |  |  |  |
| 9 | 1.05 [1H], m | 33.6 | 0.99 (H-14) | - |
|  | 1.78 [1H], m |  |  |  |
| 10 | 1.40 [1H], m | 37.5 | 0.99 (H-14) | 0.99 (H-14) |
| 11 | 3.39 [1H], dq, *J* = 5, 7 Hz | 32.9 | 1.21 (H-13) | 1.76 (H-7), 1.21 (H-13) |
| 12 | - | 172.2 | 1.21 (H-13), 5.86 (H-5) | - |
| 13 | 1.21 [3H], d, *J* = 7 Hz | 12.6 | - | 3.39 (H-11) |
| 14 | 0.99 [3H], d, *J* = 6 Hz | 19.8 | - | 1.40 (H-10) |
| 15 | 1.44 [1H], s | 25.2 | - | - |

*Artemisinin* (**22**) was purified from fraction O by isocratic column chromatography (15% EtOAc/hexane), yielding fraction O6 - 8 from which crystals of artemisinin were obtained on standing (54 mg). (Artemisinin (**22**) **was** also obtained from O3 - 5 HPLC 15; and O6 - 8 HPLC 10). Colourless crystals. LC-HRMS (*m/z*): 283.1534, Δ = -2.3 ppm for M+H^+^ (calc. 283.1540 for C_15_H_23_O_5_); R*_t_* 6.87 mins.

Deoxyartemisinin (**23**) (known^1,2^)

NMR data for deoxyartemisinin (**23**) in CDCl_3_ solution

| **Position** | **δ_H_(ppm)** | **δ_c_ (ppm)** | **HMBC** | **^1^H-^1^H COSY** |
| --- | --- | --- | --- | --- |
| 1 | 1.28 [1H], m | 44.7 | 5.70 (H-5), 1.92 (H-2), 0.94 (H-14) | - |
| 2 | 1.27 [1H], m  1.92 [1H], m | 22.0 | - | - |
| 3 | 1.62 [1H], m  1.78 [1H], m | 34.0 | - | - |
| 4 | - | 109.2 | 5.70 (H-5), 1.92 (H-2), 1.78 (H-3), 1.53 (H-15) | - |
| 5 | 5.70 [1H], s | 99.6 | 2.00 (H-7) | - |
| 6 | - | 82.5 | 5.70 (H-5), 2.00 (H-7), 1.92 (H-2) | - |
| 7 | 2.00 [1H], ddd, *J* = 13, 5, 4 Hz | 42.5 | 3.19 (H-11), 1.20 (H-13) | 3.19 (H-11), 1.92 (H-8), 1.00 (H-8) |
| 8 | 1.00 [1H], dddd, *J* = 13, 11, 11, 3 Hz  1.92 [1H], m | 23.6 | 3.19 (H-11) | 2.00 (H-7) |
| 9 | 1.09 [1H], dddd, *J* = 11, 11, 11, 3 Hz  1.80 [1H], m | 33.5 | 0.94 (H-14) | - |
| 10 | 1.28 [1H], m | 35.4 | 0.94 (H-14) | 0.94 (H-14) |
| 11 | 3.19 [1H], dq, *J* = 5, 7 Hz | 32.8 |  | 2.00 (H-7), 1.20 (H-13) |
| 12 | - | 172.0 | 5.70 (H-5), 3.19 (H-11), 2.00 (H-7), 1.20 (H-13) | - |
| 13 | 1.20 [3H], d*, J* = 7 Hz | 12.6 | 3.19 (H-11) | 3.19 (H-11) |
| 14 | 0.94 [3H], d, *J* = 6 Hz | 18.6 | - | 1.28 (H-10) |
| 15 | 1.53 [3H], s | 24.0 | - | - |

*Deoxyartemisinin* (**23**) was purified from fraction O by isocratic column chromatography (15% EtOAc/hexane), yielding fraction O3 - 5; followed by HPLC (8% EtOAc/hexane/1% AcOH), yielding O3 – 5, HPLC fraction 14. (Deoxyartemisinin (**27**) was also found in T3 HPLC 12; and O6 – 8, HPLC 6). Colourless oil. IR ν (cm^-1^): 2921 (s), 2851 (m), 1745 (m), 1669 (m), 1563 (m), 1458 (m). LC-HRMS (*m/z*): 267.1591, Δ = -0.1 ppm for MH^+^ (calc. 267.1591 for C_15_H_23_O_4_); R*_t_* 9.25 mins.

1. L.-K. Sy; G. D. Brown, "Synthesis of 6,7-dehydroartemisinic acid". *J. Chem. Soc.*, *Perkin Trans I* 2421-2429 (2001).

2. G.D. Brown; L.-K. Sy and "*In vivo* transformations of dihydroartemisinic acid in *Artemisia annua* plants". *Tetrahedron* **60** 1139-1159 (2004).

4,5-*seco*-4,5-Keto, aldehyde-amorphan-12-oic acid (**24**) (known^1,2^)

NMR data for 4,5-*seco*-4,5-keto, aldehyde-amorphan-12-oic acid (**24**) in CDCl_3_ solution

| Position | δ_H_(ppm) | δ_c_ (ppm) |
| --- | --- | --- |
| 1 | 1.46 [1H], m | 42.3 |
| 2 | 1.22 [1H], m  1.56 [1H], m | 26.7 |
| 3 | 2.32 [1H], m  2.45 [1H], m | 38.1 |
| 4 | - | 208.3 |
| 5 | 9.58 [1H], d, *J* = 5 Hz | 205.9 |
| 6 | 2.36 | 56.1 |
| 7 | 1.93 | 41.0 |
| 8 | 1.47 [1H], m  1.87 [1H], m | 23.5 |
| 9 | 1.11, [1H], m  1.81 [1H], m | 34.7 |
| 10 | 1.27 [1H] m | 33.4 |
| 11 | 2.48, [1H], m | 41.2 |
| 12 | - | 177.4 |
| 13 | 1.18 [3H] d*, J* = 7 Hz | 14.4 |
| 14 | 0.94 [3H] d, *J* = 6 Hz | 19.6 |
| 15 | 2.14 [3H] s | 30.0 |

1. G.D. Brown; G.-Y. Liang; L.-K. Sy “Terpenoids from the seeds of *Artemisia annua*”. *Phytochemistry* **64** 303-323 (2003).
2. G.D. Brown; L.-K. Sy and "*In vivo* transformations of dihydroartemisinic acid in *Artemisia annua* plants". *Tetrahedron* **60** 1139-1159 (2004).

*nor-Amorphanes*

Arteannuin Q (**25**) (known^1^) **/(26**) (novel as a natural product, although this ketal has previously been reported from a series of chemical reactions with artemisinin^2^ (as a mixture with the epimeric ketal, rather than as a mixture with keto-form, as found for this natural product))

NMR data for the thermodynamic equilibrating mixture of arteannuin Q (**25/26**) in CDCl_3_ solution

| Compound (**25**) | Compound (**26**) |
| --- | --- |

| Position | δ_H_ (ppm) | δ_C_ (ppm) | HMBC | ^1^H-^1^H COSY | δ_H_ (ppm) | δ_C_ (ppm) | HMBC | ^1^H-^1^H COSY |
| --- | --- | --- | --- | --- | --- | --- | --- | --- |
| 1 | 2.10 [1H] m | 56.7 | 1.09 (H-14) | - | 1.12 [1H] m | 50.8 | 0.97 (H-14) | - |
| 2 | 1.85 [1H] m  1.78 [1H] m | 20.1 | - | 2.39 (H-3), 2.55 H-3) | 1.52 [1H] m  2.22 [1H] m | 21.1 | - | - |
| 3 | 2.39 [1H] m  2.55 [1H] m | 41.2 | 2.14 (H-15) | 1.85 (H-2), 1.78 (H-2) | 2.60 [1H] m  2.83 [1H] m | 43.2 | 2.22 (H-15) | - |
| 4 | - | 209.5 | 2.14 (H-15) | - | - | 212.6 | 2.22 (H-15) | - |
| 6 | - | 212.8 | - | - | - | 106.6 | - | - |
| 7 | 2.65 [1H] m | 53.3 | 1.21 (H-13) | - | 2.24 [1H] dd *J* = 7, 7 Hz | 46.1 | 3.38 (H-11), 1.13 (H-13) | 3.38 (H-11) |
| 8 | 1.56 [1H] m  2.09 [1H] m | 30.5 | - | - | 0.97 [1H] m  1.74 [1H] m | 25.0 | 3.38 (H-11) | - |
| 9 | 1.55 [1H] m  1.91 [1H] m | 34.4 | 1.09 (H-14) | - | 1.04 [1H] m  1.64 [1H] m | 32.5 | 0.97 (H-14) | - |
| 10 | 1.61 [1H] m | 40.3 | 1.09 (H-14) | 1.09 (H-14) | 1.34 [1H] m | 35.7 | 0.97 (H-14) | 0.97 (H-14) |
| 11 | 2.82 [1H] dq, *J* = 7, 7 Hz | 39.1 | 1.21 (H-13) | 1.21 (H-13) | 3.38 [1H] dq, *J* = 7, 7 Hz | 39.3 | 1.13 (H-13) | 2.24 (H-7), 1.13 (H-13) |
| 12 | - | 180.1 | 1.21 (H-13) | - | - | 179.7 | 1.13 (H-13) | - |
| 13 | 1.21 [3H] d, *J* = 7 Hz | 14.5 | - | 2.82 (H-11) | 1.13 [3H] d, *J* =7 Hz | 8.9 | 3.38 (H-11) | 3.38 (H-11) |
| 14 | 1.09 [3H] d, *J* = 7 Hz | 20.6 | - | 1.61 (H-10) | 0.97 [3H] d, *J* = 7 Hz | 19.8 | - | 1.34 (H-10) |
| 15 | 2.14 [3H] s | 30.0 | - | - | 2.22 [3H] s | 30.0 | - | - |

*The equilibrating mixture of (****25****) and (****26****)* was purified from fraction T by isocratic column chromatography (50% EtOAc/hexane), yielding fraction T4 (53 mg). Colourless oil. IR ν (cm^-1^): 3500-2500 (v br), 3021 (w), 2929 (m), 2876 (w), 2853 (w), 1772 (m), 1704 (s), 1456(s). LC-HRMS (*m/z*): 255.1594, Δ = 1.1 ppm for M+H^+^ (calc. 255.1591 for C_14_H_23_O_4_); 237.1487, Δ= 0.9 ppm for M+H^+^-H_2_O (calc. 237.1485 for C_14_H_21_O_3_); 277.1413, Δ = 1.0 ppm for M+Na^+^ (calc. 277.1410 for C_14_H_22_O_4_Na); 531.2932, Δ = 0.6 ppm for M_2_ + Na^+^ (calc. 531.2928 for C_28_H_44_O_8_Na); R*_t_* 4.50 mins.

Having determined the structures of both components (**25**) and (**26**) by 2D-NMR, it became clear why these two components were chromatographically inseparable and also why they were always isolated in a constant ratio. Compounds (**25**) and (**26**) are, in fact, equilibrating forms of the same species, for which intramolecular nucleophilic attack of the C-12 carboxylic acid group at the ketone at the C-6 position in (**25**), results in an *O*-lactone-substituted hemi-ketal group in (**26**).

Further analysis of the mixture of (**25**)/(**26**) by 2D-NOESY NMR confirmed the foregoing analysis. Unusually, it was found that the NOESY spectrum of the mixture contained both negative two-dimensional peaks and positive two-dimensional peaks. Only the negative peaks for the major isomer (**25**) were clearly resolved, and these were consistent with the relative stereochemistry which is shown for (**25**). Positive peaks are not normally observed in the NOESY spectra of small molecules, and their occurrence is normally associated with rapid exchange on the NMR timescale. For this reason, such peaks are therefore sometimes referred to as “EXSY” peaks. The positive peaks for (**26)**/(**25**) therefore represent “connected” proton chemical shifts for each resolved position of the equilibrating pair (**26**)/(**25**). These “EXSY” assignments which are shown below were in full agreement with assignments obtained by more conventional 2D-NMR techniques, which are reported in the table.

Positive “EXSY” peaks observed in the NOESY spectrum of (**26**) included: 3.38→2.82 (H-11); 2.83→2.39 (H-3); 2.22 → 2.65 (H-7); 2.22 → 1.78 (H-2); 1.64 → 1.91 (H-9); 1.74 → 2.09 (H-8); 1.52 → 1.81 (H-2); 1.34 → 1.61 (H-10); 1.13 → 1.22 (H-13); 1.12 → 2.10 (H-1); 0.97 → 1.08 (H-14); and 0.97 →1.56 (H-8). All of these are the result of rapid exchange on the NMR timescale between (**26**) and (**25**), and they confirm many of the NMR assignments which are reported in the table.

1. G.D. Brown; G.-Y. Liang; L.-K. Sy “Terpenoids from the seeds of *Artemisia annua*”. *Phytochemistry* **64** 303-323 (2003).
2. F. Bousejra-El Garah; M. Pitie; L. Vendier; B. Meunier; A. Robert. “Alkylating ability of artemisinin after Cu(I)-induced activation”. *Journal of Biological Inorganic Chemistry* **14**(4), 601-610. (2009).

*Abeo-Amorphanes*

*abeo*-Amorphane sesquiterpene (**27**) (novel^1^)

NMR data for the 8,7→6 *abeo*-amorphane sesquiterpene (**27**) in CDCl_3_ solution

| **Position** | **δ_H_ (ppm)** | **δ_C_ (ppm)** | **HMBC** | **^1^H-^1^H COSY** |
| --- | --- | --- | --- | --- |
| 1 | 1.91 [1H], ddd, *J* = 10, 3, 3 Hz | 42.5 | 0.94 (H-14) | - |
| 2 | 1.26 [1H], m  1.07 [1H], m | 37.2 br | - | - |
| 3 | 1.39 [1H], m  1.58 [1H], m | 17.6 | 1.36 (H-3) | - |
| 4 | - | 58.1 | 1.36 (H-3) | - |
| 5 | 2.97 [1H], s | 63.6 | 1.36 (H-3) | - |
| 6 | - | 57.6 |  | - |
| 7 | - | 215.6 | 3.13 (H-11), 1.15 (H-13), 1.14 (H-12) | - |
| 8 | 2.07 [1H], ddd *J* = 13, 8, 2 Hz  1.56 [1H], m | 34.0 | - | 1.78 (H-9), 1.13 (H-9) |
| 9 | 1.78 [1H], m  1.13 [1H], m | 33.7 | 0.94 (H-14) | 2.07 (H-8), 1.56 (H-8) |
| 10 | 1.63 [1H], m | 36.3 | 0.94 (H-14) | 0.94 (H-14) |
| 11 | 3.13 [1H], sept *J* = 7 Hz | 36.8 | 1.15 (H-13), 1.14 (H-12) | 1.14 (H-12), 1.15 (H-13) |
| 12 | 1.14 [3H], d, *J* = 7 Hz | 20.6 | 1.15 (H-13) | 3.13 (H-11) |
| 13 | 1.15 [3H], d, *J* = 7 Hz | 19.7 | 1.14 (H-12) | 3.13 (H-11) |
| 14 | 0.94 [3H], d, *J* = 7 Hz | 17.7 | - | 1.63 (H-10) |
| 15 | 1.36 [3H], s | 23.2 | - | - |

8,7→6 abeo*-Amorphane sesquiterpene* (**27**) was purified from fraction D by isocratic column chromatography (6% EtOAc/hexane), yielding fraction D4; followed by HPLC (6% EtOAc/hexane), yielding D4, HPLC fraction 10 (*R_t_* 13.2 mins). Colourless oil. LC-HRMS (*m/z*): 237.1849, Δ = -0.1 ppm for M+H^+^ (calc. 237.1849 for C_15_H_25_O_2_).

1. The 3α-hydroxy derivative of novel metabolite 8,7→6 *abeo-*amorphane (**27**) had been reported previously as a natural product from *A. annua*: G. D. Brown; G.-Y. Liang; L.-K. Sy “Terpenoids from the seeds of *Artemisia annua*”. *Phytochemistry* **64** 303-323 (2003).

*Aromadendrane sesquiterpenes*

Spathulenol (**28**) (known)

NMR data for spathulenol (**28**) in CDCl_3_ solution

| **Position** | **δ_H_ (ppm)** | **δ_C_ (ppm)** | **HMBC** | **H-^1^H COSY** |
| --- | --- | --- | --- | --- |
| 1 | 2.21 [1H], m | 53.4 | 4.69 (H-14),4.67 (H-14) | - |
| 2 | 1.64 [1H], dddd, *J* = 12, 6 ,6, 2 Hz  1.94 [1H], m | 26.8 | - | - |
| 3 | 1.58 [1H], m  1.78 [1H], ddd *J* = 13, 6, 6 Hz | 41.7 | 1.29 (H-15) | - |
| 4 | - | 80.8 | 1.29 (H-15) | - |
| 5 | 1.31 [1H], m | 54.3 | 1.29 (H-15) | 0.47 (H-6) |
| 6 | 0.47 [1H], dd, *J* = 12, 10 Hz | 29.5 | 1.05 (H-12),1.06 (H-13) | 1.31 (H-5), 0.72 (H-7) |
| 7 | 0.72 [1H], ddd, *J* = 15, 10, 6 Hz | 27.5 | 1.05 (H-12),1.06 (H-13) | 1.99 (H-8), 1.02 (H-8), 0.47 (H-6) |
| 8 | 1.02 [1H], ddd, *J* = 13, 12, 12 Hz  1.99 [1H], ddd, *J* = 13, 6, 6 Hz | 24.8 | - | 0.72 (H-7) |
| 9 | 2.04 [1H], dd, *J* = 12, 12 Hz  2.43 [1H], dd, *J* = 14, 7 Hz | 38.9 | 4.69 (H-14), 4.67 (H-14) | - |
| 10 | - | 153.4 | - | - |
| 11 | - | 20.3 | 1.05 (H-12), 1.06 (H-13) | - |
| 12 | 1.05 [3H], s | 16.4 | 1.06 (H-13) | - |
| 13 | 1.06 [3H], s | 28.7 | 1.05 (H-12 | - |
| 14 | 4.69 [1H], s  4.67 [1H], s | 106.5 | - | - |
| 15 | 1.29 [3H], s | 26.1 | - | - |

*Spathulenol* (**28**) was purified from fraction I by isocratic column chromatography (5% EtOAc/hexane), yielding fraction I3 - 5; followed by HPLC (5% EtOAc/hexane), yielding I3 - 5 HPLC fraction 14 (*R_t_* 29 mins). (Spathulenol (**28**) was also found in Fraction J - HPLC 16; and fraction K - HPLC 20). Colourless oil. IR ν (cm^-1^): 3428 (br), 2922 (s), 2852 (m), 1715 (m), 1635 (m), 1455 (m).

1. *Diterpenes*

Phytol (**29**) (known^1^)

NMR data for phytol (**29**) in CDCl_3_ solution

| **Position** | **δ_H_ (ppm)** | **δ_C_ (ppm)** | **HMBC** | **^1^H-^1^H COSY** |
| --- | --- | --- | --- | --- |
| 1 | 4.16 [2H], d, *J* = 7 Hz | 59.4 | - | 5.41 (H-2) |
| 2 | 5.41 [1H], t, *J* = 7 Hz | 123.0 | 4.16 (H-1), 1.99 (H-4), 1.67 (H-17) | 4.16 (H-1) |
| 3 | - | 140.4 | 4.16 (H-1), 1.99 (H-4), 1.67 (H-17) | - |
| 4 | 1.99 [2H], t, *J* = 7 Hz | 40.0 | 5.41 (H-2), 1.67 (H-17) | 1.44 (H-5), 1.37 (H-5), |
| 5 | 1.37 [1H], m | 25.1 | - | 1.99 (H-4), 1.28 (H-6), 1.09 (H-6) |
|  | 1.44 [1H], m |  |  |  |
| 6 | 1.09 [1H], m | 36.7 | 0.86 (H-18) | 1.44 (H-5), 1.37 (H-7) |
|  | 1.28 [1H], m |  |  |  |
| 7 | 1.37 [1H], m | 32.7 | 0.86 (H-18) | 1.28 (H-6), 1.09 (H-6), 0.86 (H-18) |
| 8* | 1.06 [1H], m | 37.4 | 0.86 (H-18) | - |
|  | 1.25 [1H], m |  |  |  |
| 9 | 1.18 [1H], m | 24.5 | - | - |
|  | 1.32 [1H], m |  |  |  |
| 10* | 1.06 [1H], m | 37.4 | 0.85 (H-19) | - |
|  | 1.25 [1H], m |  |  |  |
| 11 | 1.37 [1H], m | 32.8 | 0.85 (H-19) | - |
| 12* | 1.06 [1H], m | 37.3 | 0.85 (H-19) | - |
|  | 1.25 [1H], m |  |  |  |
| 13 | 1.20 [1H], m | 24.8 | - | - |
|  | 1.26 [1H], m |  |  |  |
| 14 | 1.14 [2H], m | 39.4 | 0.87 (H-16), 0.87 (H-20) | - |
| 15 | 1.52 [1H], m | 28.0 | 0.87 (H-16), 0.87 (H-20) | 0.87 (H-16), 0.87 (H-20) |
| 16 | 0.87 [3H], d, *J* = 7 Hz | 22.6 | 0.87 (H-20) | 1.52 (H-15) |
| 17 | 1.67 [3H], s | 16.2 | 5.41 (H-2) | - |
| 18 | 0.86 [3H], d, *J* = 7 Hz | 19.7 | - | 1.37 (H-7) |
| 19 | 0.85 [3H], d, *J* = 7 Hz | 19.8 | - | 1.37 (H-11) |
| 20 | 0.87 [3H], d, *J* = 7 Hz | 22.7 | 0.87 (H-16) | 1.52 (H-15) |

- Interchangeable assignments

1. G. D. Brown. "Phytene-1,2-diol from *Artemisia annua*". *Phytochemistry* **36** 1553-1554 (1994).

1. *Triterpenes*

Stigmasterol (**30**) (known)

NMR data for stigmasterol (**30**) in CDCl_3_ solution

| **Position** | **δ_H_ (ppm)** | **δ_C_ (ppm)** | **HMBC** | **^1^H-^1^H COSY** |
| --- | --- | --- | --- | --- |
| 1 | 1.85 [1H], m | 37.3 | 1.01 (H-24) | - |
|  | 1.08 [1H], m |  |  |  |
| 2 | 1.84 [1H], m | 31.7 | - | 3.52 (H-3)- |
|  | 1.50 [1H], m |  |  |  |
| 3 | 3.52 [1H], dddd, *J* = 10, 10, 5, 5 Hz | 71.8 | - | 2.29 (H-4), 2.24 (H-4), 1.84 (H-2), 1.50 (H-2) |
| 4 | 2.29 [1H], m | 42.3 | 5.35 (H-6) | 3.52 (H-3) |
|  | 2.24 [1H], m |  |  |  |
| 5 | - | 140.8 | 1.01 (H-24) | - |
| 6 | 5.35 [1H], br | 121.8 | - | 1.98 (H-7) |
| 7 | 1.98 [1H], m | 31.7 | - | 5.35 (H-6) |
|  | 1.50 [1H], m |  |  |  |
| 8 | 1.91 [1H], m | 31.8 | 5.35 (H-6) | - |
| 9 | 0.93 [1H], m | 50.1 | 1.01 (H-24) | - |
| 10 | - | 36.5 | 5.35 (H-6), 1.01 (H-24) | - |
| 11 | 1.44 [1H], m | 21.5 |  | - |
|  | 1.38 [1H], m |  |  |  |
| 12 | 2.01 [1H], m | 34.7 | 0.69 (H-25) | - |
|  | 1.18 [1H], m |  |  |  |
| 13 | - | 42.3 | 0.69 (H-25) | - |
| 14 | 1.15 [1H], m | 56.0 | 0.69 (H-25) | - |
| 15 | 1.67 [1H], m | 28.0 | - | - |
|  | 1.63 [1H], m |  |  |  |
| 16 | 1.43 [1H], m | 24.6 | - | - |
|  | 1.05 [1H], m |  |  |  |
| 17 | 1.00 [1H], m | 56.9 | 5.15 (H-19), 1.02 (H-26), 0.69 (H-25) | - |
| 18 | 2.04 [1H], m | 40.5 | 5.15 (H-19), 5.01 (H-20), 1.02 (H-26) | 1.02 (H-26) |
| 19 | 5.15 [1H], dd, *J* = 15, 8 Hz | 138.5 | 1.02 (H-26) | 2.04 (H-18) |
| 20 | 5.01 [1H], dd, *J* = 15, 8 Hz | 129.2 | 5.15 (H-19) | 1.54 (H-21) |
| 21 | 1.54 [1H], m | 51.2 | 5.15 (H-19), 5.01 (H-20), 0.90 (H-29), 0.84 (H-23) | - |
| 22 | 1.45 [1H], m | 31.9 | 5.01 (H-20), 0.90 (H-29), 0.84 (H-23) | 0.90 (H-29), 0.84 (H-23) |
| 23 | 0.84 [3H], d, *J* = 7 Hz | 21.1 | - | 1.45 (H-22) |
| 24 | 1.01 [3H], s | 19.4 | - | - |
| 25 | 0.69 [3H], s | 12.1 | - | - |
| 26 | 1.02 [3H], d, *J* = 7 Hz | 21.2 | - | 2.04 (H-18) |
| 27 | 1.42 [1H], m | 25.4 | - | 0.80 (H-28) |
|  | 1.32 [1H], m |  |  |  |
| 28 | 0.80 [3H], t, *J* = 7 Hz | 12.3 | - | 1.42 (H-27), 1.32 (H-27) |
| 29 | 0.90 [3H], d, *J* = 7 Hz | 19.0 | - | 1.45 (H-22) |

1. *Aliphatic hydrocarbons, alcohols, aldehydes and acids*

Polyacetylene diol (**31**) (known^1,2^)

NMR data for polyacetylene diol (**31**) in CDCl_3_ solution

| **Position** | **δ_H_ (ppm)** | **δ_c_ (ppm)** | **HMBC** | **^1^H-^1^H COSY** |
| --- | --- | --- | --- | --- |
| 1 | 5.53 [1H], d, *J* = 16 Hz  5.41 [1H], d, *J* = 11 Hz | 121.6 | 5.68 (H-2), 3.63 (H-3) | 5.68 (H-2) |
| 2 | 5.68 [1H], ddd, *J* = 16, 11, 6 Hz | 131.4 | 5.53 (H-1), 3.63 (H-3) | 5.53 (H-1), 5.41 (H-1), 3.63 (H-3) |
| 3 | 3.63 [1H], dd, *J* = 8, 6 Hz | 59.6 | 5.68 (H-2), 5.53 (H-1) | 5.68 (H-2), 3.61 (H-4) |
| 4 | 3.61 [1H], dd, *J* = 8, 6 Hz | 57.8 | 6.19 (H-5), 5.91 (H-6) | 6.19 (H-5), 3.63 (H-3) |
| 5 | 6.19 [1H], dd, *J* = 17, 6 Hz | 141.6 | 5.91 (H-6), 3.61 (H-4) | 5.91 (H-6), 3.61 (H-4) |
| 6 | 5.91 [1H], d, *J* = 17 Hz | 113.3 | 3.61 (H-4) | 6.19 (H-5) |
| 7 | - | 78.7* | - | - |
| 8 | - | 68.1* | - | - |
| 9 | - | 64.8* | - | - |
| 10 | - | 58.7* | - | - |
| 11 | - | 62.0* | 2.00 (H-13) | - |
| 12 | - | 72.8* | 2.00 (H-13) | - |
| 13 | 2.00 [3H], s | 4.7 | - | - |

*Interchangeable assignments

*Polyacetylene diol* (**31**) was purified from fraction D by isocratic column chromatography (6% EtOAc/hexane), yielding fraction D4; followed by HPLC (6% EtOAc/hexane), yielding D4 HPLC fraction 17 (*R_t_* 19.7 mins). Colourless oil.

1. F. Bohlmann; C. Zdero. “Polyacetylenic compounds. 174. New spiroketal enol ether polynes from species of the tribe *Anthemidae*” *Chemische Berichte* **103**(6), 1879-1885 (1970).
2. E. Ivarsen; X. C. Frette; K. B. Christensen; L. P. Christensen; R. M. Engberg; K. Grevsen; A. Kjaer. “Bioassay-guided chromatographic isolation and identification of antibacterial compounds from *Artemisia annua* L. that inhibit *Clostridium perfringens* growth”. *Journal of AOAC International* **97**(5), 1282-1290 (2014). This paper reports NMR data for (+)-*threo*-(5*E*)-trideca-1,5-diene-7,9,11-triyne-3,4-diol, which was recorded from solution in d_4_-MeOH. It is not therefore possible to make a direct comparison with data in the above Table, which was recorded in CDCl_3_ solution. However, based on the sizeable upfield shifts at positions 3-and 4- in the Table relative to the literature compound, natural product (**31**) might tentatively be assigned as the *erythro-* isomer.

Pontica epoxide (**32**) (known)

NMR data for pontica epoxide (**32**) in CDCl_3_ solution

| **Position** | **δ_H_ (ppm)** | **δ_c_ (ppm)** | **HMBC** | **^1^H-^1^H COSY** |
| --- | --- | --- | --- | --- |
| 1 | 5.33 [1H], d, *J* = 10 Hz | 120.2 | 5.59 (H-2), 3.26 (H-3) | 5.59 (H-2) |
|  | 5.51 [1H], d, *J* = 17 Hz |  |  |  |
| 2 | 5.59 [1H], ddd, *J* = 17, 10, 7 Hz | 134.2 | 5.51 (H-1), 3.26 (H-3) | 5.51 (H-1), 5.33 (H-1), 3.26 (H-3) |
| 3 | 3.26 [1H], dd, *J* = 7, 2 Hz | 61.3 | 5.59 (H-2), 5.51 (H-1) | 5.59 (H-2) |
| 4 | 3.29 [1H], dd, *J* = 7, 2 Hz | 59.0 | 6.12 (H-5), 5.86 (H-6) | 6.12 (H-5) |
| 5 | 6.12 [1H], dd, *J* = 16, 7 Hz | 143.9 | 5.86 (H-6), 3.29 (H-4) | 5.86 (H-6), 3.29 (H-4) |
| 6 | 5.86 [1H], d, *J* = 16 Hz | 112.5 | 3.29 (H-4) | 6.12 (H-5) |
| 7 | - | 76.6* | 6.12 (H-5) | - |
| 8 | - | 58.7* | 5.86 (H-6) | - |
| 9 | - | 72.8* | - | - |
| 10 | - | 78.7* | - | - |
| 11 | - | 68.0* | 1.96 (H-13) | - |
| 12 | - | 64.8* | 1.96 (H-13) | - |
| 13 | 1.96 [3H], s | 4.7 | - | - |

*Interchangeable Assignments

*Pontica epoxide (****32)*** was purified from fraction D by isocratic column chromatography (6% EtOAc/hexane), yielding fraction D4; followed by HPLC (6% EtOAc/hexane), yielding D4, HPLC fraction 11 (*R_t_* 13.8 mins). (Pontica epoxide (**32**) was also found in Fraction E, HPLC 7; Fraction G, HPLC 7; and Fraction H, HPLC 5). Colourless oil.

Annua diepoxide (**33**) (known^1^)

NMR data for annua diepoxide (**33**) in CDCl_3_ solution

| **Position** | **δ_H_ (ppm)** | **δ_c_ (ppm)** | **HMBC** | **^1^H-^1^H COSY** |
| --- | --- | --- | --- | --- |
| 1 | 5.53 [1H], dd, *J* = 13, 3 Hz | 120.9 | 3.36 (H-3) | 5.54 (H-2) |
|  | 5.34 [1H], dd, *J* = 10.3 Hz |  |  |  |
| 2 | 5.54 [1H], ddd, *J* = 13, 10, 6 Hz | 133.8 | 5.53 (H-1), 5.34 (H-1), 3.36 (H-3) | 5.34 (H-1), 3.36 (H-3) |
| 3 | 3.36 [1H], dd, *J* = 6, 2 Hz | 56.1 | 5.54 (H-2), 5.34 (H-1) | 5.54 (H-2), 3.32 (H-4) |
| 4 | 3.32 [1H], dd, *J* = 3, 2 Hz | 57.6 | 3.44 (H-6) | 3.36 (H-3), 2.99 (H-5) |
| 5 | 2.99 [1H], dd *J* = 3, 2 Hz | 56.3 | 5.54 (H-2) | 3.44 (H-6), 3.32 (H-4), |
| 6 | 3.44 [1H], d, *J* = 2 Hz | 43.1 | - | 2.99 (H-5) |
| 7 | - | 77.3* | 3.44 (H-6) | - |
| 8 | - | 58.7* | 3.44 (H-6) | - |
| 9 | - | 71.9* | - | - |
| 10 | - | 78.7* | - | - |
| 11 | - | 68.0* | 1.98 (H-13) | - |
| 12 | - | 64.8* | 1.98 (H-13) | - |
| 13 | 1.98 [3H], s | 4.7 | - | - |

* Interchangeable assignments

*Annua diepoxide* (**33**) was purified from fraction G by HPLC (6% EtOAc/hexane), yielding G HPLC fraction 12 (*R_t_* 20.8 mins). (Annua diepoxide (**33**) was also found in fraction E, HPLC 10; and fraction H, HPLC 8). Colourless oil.

1. D. Manns; R. Hartmann “Annuadiepoxide, a new polyacetylene from the aerial parts of *Artemisia annua*”. *Journal of Natural Products*  **55**(1), 29-32 (1992).

*vi) Aromatic alcohols, ketones and acids*

2,4-Dihydroxy-6-methoxy acetophenone (**34**) (known^1^)

NMR data for 2,4-dihydroxy-6-methoxy acetophenone (**34**) in CDCl_3_ solution

| **Position** | **δ_H_ (ppm)** | **δ_c_ (ppm)** | **HMBC** | **^1^H-^1^H COSY** |
| --- | --- | --- | --- | --- |
| 1 | - | 106.1 | 5.98 (H-3), 5.91 (H-5), 2.61 (H-8) | - |
| 2 | 167.3 [1H], m | 167.3 | 5.98 (H-3) | - |
| 3 | 5.98 [1H], d, *J* = 2 Hz | 96.4 | 5.91 (H-5) | 5.91 (H-5) |
| 4 | - | 163.7 | 3.87 (H-9) | - |
| 5 | 5.91 [1H], d, *J* = 2 Hz | 90.5 | 5.98 (H-3) | 5.98 (H-3) |
| 6 | - | 162.5 | 5.98 (H-3), 5.91 (H-5) | - |
| 7 | - | 203.3 | 2.61 (H-8) | - |
| 8 | 2.61 [3H], s | 33.0 | - | - |
| 6-OMe | 3.87 [3H], s | 55.6 | - | - |

*2,4-Dihydroxy-6-methoxy acetophenone* (**34**) was puriified from fraction T by isocratic column chromatography (50% EtOAc/hexane), yielding fraction T3; followed by HPLC (15% EtOAc/hexane/1% AcOH), yielding T3 HPLC fraction 18 (*R_t_*  39 mins). Colourless oil. IR ν (cm^-1^): 3394 (v br), 2958 (sh), 2923 (s), 2851 (m), 1715 (m), 1623 (s), 1597 (s), 1461 (m). LC-HRMS (*m/z*): 183.0650, Δ = -1.2 ppm for M+H^+^ (calc. 183.0652 for C_9_H_11_O_4_); R*_t_* 4.15 mins. HRAPCIMS (*m/z*): 183.0648, Δ = 2.2 ppm for [M+H]^+^ (calc. 183.0652 for C_9_H_11_O_4_).

1. G. D. Brown. "Two new compounds from *Artemisia annua*". *J. Nat. Prod.* **55** 1756-1770 (1992).

5-Nonadecyl resorcinol-3-*O*-methyl ether (**35**) (known^1^)

NMR data for 5-nonadecyl resorcinol-3-*O*-methyl ether (**35**) in CDCl_3_ solution

| **Position** | **δ_H_ (ppm)** | **δ_c_ (ppm)** | **HMBC** | **^1^H-^1^H COSY** |
| --- | --- | --- | --- | --- |
| 1 | - | 156.5 | 6.26 (H-6), 6.23 (H-2), 4.86 (5-OH) | - |
| 2 | 6.23 [1H], dd, *J* = 2, 2 Hz | 98.6 | 6.32 (H-4), 6.26 (H-6), 4.86 (5-OH) | 6.32 (H-4), 6.26 (H-6) |
| 3 | - | 160.8 | 6.32 (H-4), 6.23 (H-2), 3.77 (3-OMe) | - |
| 4 | 6.32 [1H], dd, *J* = 2, 2 Hz | 106.7 | 6.26 (H-6), 6.23 (H-2), 2.51 (H-7) | 6.26 (H-6), 6.23 (H-2) |
| 5 | - | 145.8 | 2.51 (H-7) | - |
| 6 | 6.26 [1H], dd, *J* = 2, 2 Hz | 107.9 | 6.32 (H-4), 4.85 (5-OH), 2.51 (H-7) | 6.32 (H-4), 6.23 (H-2) |
| 7 | 2.51 [2H], t, *J* = 7 Hz | 36.1 | 6.32 (H-4), 6.26 (H-6) | 1.57 (H-8) |
| 8 | 1.57 [2H], quin, *J* = 7 Hz | 31.2 | 2.51 (H-7) | 2.51 (H-7) |
| 9 | 1.30 [2H], br | 28.9 | 2.51 (H-7) | - |
| 10 | 1.30 [2H], br | 28.8 | 1.57 (H-8) | - |
| 11-22 | 1.26 [24H], br | 28.6 | - | - |
| 23 | 1.25 [2H], br | 31.9 | 1.29 (H-26), 0.88 (H-27) | - |
| 24 | 1.29 [2H], br | 22.7 | 0.88 (H-27) | 0.88 (H-25) |
| 25 | 0.88 [3H], t, *J* = 7 Hz | 14.1 | - | 1.29 (H-24) |
| 3-OMe | 3.77 [3H], s | 55.2 | - | - |

*5-Nonadecyl resorcinol-3-O-methyl ether* (**35**) was purified from fraction I by isocratic column chromatography (5% EtOAc/hexane), yielding fraction I6 (14 mg). (5-Nonadecyl resorcinol-3-*O*-methyl ether (**35**) was also found in I3 – 5,HPLC fraction 11 (5% EtOAc/hexane, *R_t_* 23 mins); and fraction J, HPLC 12). Colourless oil. IR ν (cm^-1^): 3422 (m), 2916 (s), 2849 (s), 1617 (m), 1595 (m), 1504 (w), 1464 (m).

1. G.D. Brown. "Two new compounds from *Artemisia annua*". *J. Nat. Prod.* **55** 1756-1770 (1992).
2. *Phenylpropanoids*

Eugenol (**36**) (known)

NMR data for eugenol (**36**) in CDCl_3_ solution

| **Position** | **δ_H_ (ppm)** | **δ_C_ (ppm)** | **HMBC** | **^1^H-^1^H COSY** |
| --- | --- | --- | --- | --- |
| 1 | - | 132.2 | 6.84 (H-5), 3.32 (H-7) | - |
| 2 | 6.68 [1H], m | 111.1 | 6.68 (H-6), 3.32 (H-7) | - |
| 3 | - | 146.3 | 6.68 (H-2), 6.84 (H-5), 3.88 (3-OMe) | - |
| 4 | - | 143.9 | 6.84 (H-5), 6.68 (H-2) | - |
| 5 | 6.84 [1H], d, *J* = 8 Hz | 114.3 | - | 6.68 (H-6) |
| 6 | 6.68 [1H], m | 121.2 | 6.84 (H-5), 3.32 (H-7) | 6.84 (H-5) |
| 7 | 3.32 [2H], d, *J*= 7 Hz | 39.9 | - | 5.95 (H-8) |
| 8 | 5.95 [1H], ddt, *J* = 17, 10, 7 Hz | 137.9 | 3.32 (H-7) | 5.08 (H-9), 5.05 (H-9), 3.32 (H-7) |
| 9 | 5.08 [1H] d, *J* = 17 Hz  5.05 [1H] d, *J* = 10 Hz | 115.6 | 3.32 (H-7) | 5.95 (H-8) |
| 3-OMe | 3.88 [3H] s | 55.9 | - | - |

*Eugenol* (**36**) was purified from fraction K by HPLC (5% EtOAc/hexane/1%AcOH), yielding HPLC fraction 15 (*R_t_* 19.5 mins). Eugenol (**36**) was also found in fraction J - HPLC 12. Colourless oil.

Coumarin (**37**) (known)

NMR data for coumarin (**37**) in CDCl_3_ solution

| **Position** | **δ_H_ (ppm)** | **δ_C_ (ppm)** | **HMBC** | **^1^H-^1^H COSY** |
| --- | --- | --- | --- | --- |
| 2 | - | 160.9 | 7.73 (H-4), 6.43 (H-3) | - |
| 3 | 6.43 [1H], d, *J* = 10 Hz | 116.7 | 7.73 (H-4) | 7.73 (H-4) |
| 4 | 7.73 [1H], d, *J* = 10 Hz | 143.5 | 7.50 (H-5) | 6.43 (H-3) |
| 5a | - | 118.9 | 7.73 (H-4), 7.33 (H-8), 7.29 (H-6), 6.43 (H-3) | - |
| 5 | 7.50 [1H], dd *J* = 8, 2 Hz | 127.9 | 7.73 (H-4), 7.54 (H-7) | 7.54 (H-7), 7.29 (H-6) |
| 6 | 7.29 [1H], ddd, *J* = 8, 8, 2 Hz | 124.5 | 7.33 (H-8) | 7.54 (H-7), 7.50 (H-5), 7.33 (H-8) |
| 7 | 7.54 [1H], ddd, *J* = 8, 8, 2 Hz | 131.8 | 7.50 (H-5) | 7.50 (H-5), 7.33 (H-8), 7.29 (H-6) |
| 8 | 7.33 [1H], dd, *J* = 8, 2 Hz | 116.9 | 7.29 (H-6) | 7.54 (H-7), 7.29 (H-6) |
| 8a | - | 154.1 | 7.73 (H-4), 7.54 (H-7), 7.50 (H-5), 7.33 (H-8) | - |

*Coumarin* (**37**) was purified from fraction O by isocratic column chromatography (15% EtOAc/hexane), yielding fraction O9 - 13 (244 mg; some artemisinin (**22**) also present). Colourless oil. IR ν (cm^-1^): 2956 (sh), 2928 (m), 2871 (w), 2854 (w), 1734 (s), 1607 (m), 1565 (w), 1454 (w). LC-HRMS (*m/z*): 147.0437, Δ = -2.1 ppm for MH^+^ (calc. 147.0441 for C_9_H_7_O_2_); R*_t_* 3.33 mins. {Artemisinin impurity: (*m/z*): 283.1541, Δ = 0.2 ppm for MH^+^ (calc. 283.1540 for C_15_H_23_O_5_)}. ESI-MS (*m/z*): 147.0041, ∆ = -1.2 ppm for [M + H^+^], C_9_H_7_O_2_; R_t_ 4.08 mins.

Scopoletin (**38**) (known^1^)

NMR data for scopoletin (**38**) in CDCl_3_ solution

| **Position** | **δ_H_ (ppm)** | **δ_C_ (ppm)** | **HMBC** | **^1^H-^1^H COSY** |
| --- | --- | --- | --- | --- |
| 2 | - | 161.8 | 7.61 (H-4), 6.27 (H-3) | - |
| 3 | 6.27 [1H], d, *J* = 9 Hz | 113.2 | - | 7.61 (H-4) |
| 4 | 7.61 [1H], d, *J* = 9 Hz | 143.6 | 6.84 (H-5) | 6.27 (H-3) |
| 5a | - | 111.5 | 6.91 (H-8), 6.27 (H-3) | - |
| 5 | 6.84 [1H], s | 107.6 | 7.61 (H-4) | - |
| 6 | - | 144.2 | 6.91 (H-8), 3.95 (6-OMe) | - |
| 7 | - | 149.9 | 6.91 (H-8), 6.84 (H-5) | - |
| 8 | 6.91 [1H], s | 103.2 | - | - |
| 8a | - | 150.1 | 7.61 (H-4), 6.84 (H-5) | - |
| 6-OMe | 3.95 [3H], s | 56.4 | - | - |

*Scopoletin (****38****).* HRAPCIMS (*m/z*): 193.0494, Δ = 0.9 ppm for [M+H]^+^ (calc. 193.0495 for C_10_H_9_O_4_).

1. D. V. Banthorpe; G. D. Brown. "Two unexpected coumarins from tissue cultures of Compositae species". *Phytochemistry* **28** 3003-3007 (1989).

Isofraxidin (**39**) (known^1^)

NMR data for isofraxidin (**39**) in CDCl_3_ solution

| **Position** | **δ_H_ (ppm)** | **δ_C_ (ppm)** | **HMBC** | **^1^H-^1^H COSY** |
| --- | --- | --- | --- | --- |
| 2 | - | 160.5 | 7.61 (H-4), 6.28 (H-3) | - |
| 3 | 6.28 [1H], d, *J* = 9 Hz | 113.6 | - | 7.61 (H-4) |
| 4 | 7.61 [1H], d, *J* = 9 Hz | 143.9 | - | 6.28 (H-3) |
| 5a | - | 110.4 | - | - |
| 5 | 6.66 [1H], s | 103.2 | - | - |
| 6 | - | 144.6 | 3.95 (6-OMe) | - |
| 7 | - | 142.4 | 6.66 (H-5) | - |
| 8 | - | 134.5 | 4.10 (8-OMe) | - |
| 8a | - | 143.2 | 7.61 (H-4), 6.84 (H-5) | - |
| 6-OMe | 3.95 [3H], s | 56.5 | - | - |
| 8-OMe | 4.10 [3H], s | 61.5 | - | - |

*Isofraxidin (****39****).* ESI-MS (*m/z*): 223.0601, ∆ = 0.1 ppm for [M + H^+^], C_11_H_11_O_5_; R_t_ 3.12 mins. HRAPCIMS (*m/z*): 223.0602, Δ = -0.3 ppm for [M+H]^+^ (calc. 223.0601 for C_11_H_11_O_5_).

1. D .V. Banthorpe; G. D. Brown. "Two unexpected coumarins from tissue cultures of Compositae species". *Phytochemistry* **28** 3003-3007 (1989).

*viii) Flavonoids*

Chrysosplenetin (**40**) (known^1^)

NMR data for chrysosplenetin (**40**) in CDCl_3_ solution

| **Position** | **δ_H_ (ppm)** | **δ_C_ (ppm)** | **HMBC** | **^1^H-^1^H COSY** |
| --- | --- | --- | --- | --- |
| 2 | - | 156.1 | 7.70 (H-2’), 7.66 (H-6’) | - |
| 3 | - | 138.6 | 3.85 (3-OMe) | - |
| 4 | - | 178.9 | - | - |
| 5a | - | 106.5 | 12.61 (5-OH), 6.51 (H-8) | - |
| 5 | - | 152.6 | 12.61 (5-OH) | - |
| 6 | - | 132.2 | 12.61 (5-OH), 6.51 (H-8), 3.93 (6-OMe) | - |
| 7 | - | 158.8 | 6.51 (H-8), 3.97 (7-OMe) | - |
| 8 | 6.51 [1H], s | 90.34 | - | - |
| 8a | - | 152.3 | 6.51 (H-8) | - |
| 1’ | - | 122.2 | 7.70 (H-2’), 7.66 (H-6’) | - |
| 2’ | 7.70 [1H], d, *J* = 2 Hz | 111.0 | 7.66 (H-6’) | 7.66 (H-6’) |
| 3’ | - | 146.5 | 7.70 (H-2’), 7.04 (H-5’), 3.98 (3’-OMe) | - |
| 4’ | - | 148.6 | 7.70 (H-2’), 7.67 (H-6’), 7.04 (H-5’) | - |
| 5’ | 7.04 [1H], d, *J* = 8 Hz | 114.8 | - | 7.66 (H-6’) |
| 6’ | 7.66 [1H], dd, *J* = 8, 2 Hz | 122.6 | 7.04 (H-5’) | 7.70 (H-2’), 7.04 (H-5’) |
| 3-OMe | 3.85 [3H], s | 60.2 | - | - |
| 6-OMe | 3.93 [3H], s | 60.9 | - | - |
| 7-OMe | 3.97 [3H], s | 56.4 | - | - |
| 3’-OMe | 3.98 [3H], s | 56.1 | - | - |

*Chrysosplenetin* (**40**) was purified from fraction T by isocratic column chromatography (70% EtOAc/hexane), yielding fractions T10 - 14 (25 mg), T15 - 19 (15 mg) and T20 - 24 (20 mg), which were combined together. Colourless oil. IR ν (cm^-1^): 3394 (br w), 2958 (sh), 2927 (s), 2855 (m), 1713 (s), 1658 (m), 1595 (s), 1461 (s). LC-HRMS (*m/z*): 375.1075, Δ = 0.0 ppm for M+H^+^ (calc. 375.1074 for C_19_H_19_O_8_); 771.1901, Δ = 0.7 ppm for M_2 +_ Na^+^ (calc. 771.1896 for C_38_H_36_O_16_Na); R*_t_* 7.64 mins.

1. L.-K. Sy and G. D. Brown “Three sesquiterpenes from *Artemisia annua*”. *Phytochemistry* **48**(7) 1207-1211 (1998).

Casticin (**41**) (known^1^)

NMR data for casticin (**41**) in CDCl_3_ solution

| **Position** | **δ_H_ (ppm)** | **δ_C_ (ppm)** | **HMBC** | **^1^H-^1^H COSY** |
| --- | --- | --- | --- | --- |
| 2 | - | 155.8 | 7.71 (H-6’), 7.57 (H-2’) | - |
| 3 | - | 138.9 | 3.86 (3-OMe) | - |
| 4 | - | 178.9 | - | - |
| 5a | - | 106.5 | 6.51 (H-8) | - |
| 5 | - | 152.6 | - | - |
| 6 | - | 132.1 | 6.51 (H-8), 3.92 (6-OMe) | - |
| 7 | - | 158.8 | 6.51 (H-8), 3.96 (7-OMe) | - |
| 8 | 6.51 [1H], s | 90.4 | - | - |
| 8a | - | 152.3 | 6.51 (H-8) | - |
| 1’ | - | 123.3 | 6.96 (H-5’) | - |
| 2’ | 7.67 [1H], d, *J* = 2 Hz | 114.5 | 7.71 (H-6’) | 7.71 (H-6’) |
| 3’ | - | 145.6 | 7.67 (H-2’), 6.96 (H-5’), 3.99 (4’-OMe) | - |
| 4’ | - | 149.0 | 6.96 (H-5’) | - |
| 5’ | 6.96 [1H], d, *J* = 2 Hz | 110.5 | - | 7.71 (H-6’) |
| 6’ | 7.71 [1H], dd, *J* = 8, 2 Hz | 121.5 | 6.96 (H-5’) | 7.67 (H-2’), 6.96 (H-5’) |
| 3-OMe | 3.86 [3H], s | 60.2 | - | - |
| 6-OMe | 3.92 [3H], s | 61.0 | - | - |
| 7-OMe | 3.96 [3H], s | 56.3 | - | - |
| 4’-OMe | 3.99 [3H], s | 56.0 | - | - |

*Casticin (****41****)* was purified as for chrysosplenetin (**40**) (the two have very similar chromatographic properties). Colourless oil. HRAPCIMS (*m/z*): 375.1092, Δ = -3.8 ppm for [M+H]^+^ (calc. 375.1077 for C_19_H_19_O_8_).

1. G. D. Brown; G.-Y. Liang; L.-K. Sy “Terpenoids from the seeds of *Artemisia annua*”. *Phytochemistry* **64** 303-323 (2003).

**Supplementary List 2.** Natural products isolated and characterized from the NCV variety of *A. annua* by the by the preparative HPLC/ 1D- and 2D-NMR approach.

1. *Monoterpenes*

Artemisia ketone (**42**) (known)

NMR data for artemisia ketone (**42**) in CDCl_3_ solution

| **Position** | **δ_H_ (ppm)** | **δ_C_ (ppm)** | **HMBC** | **^1^H-^1^H COSY** |
| --- | --- | --- | --- | --- |
| 1 | 5.14 [1H], d, *J* = 18 Hz | 113.8 | - | 5.93 (H-2) |
|  | 5.12 [1H], d, *J* = 10 Hz |  |  |  |
| 2 | 5.93 [1H], dd, *J* = 18, 10 Hz | 143.2 | 1.22 (H-9/10) | 5.14 (H-1), 5.12 (H-1) |
| 3 | - | 50.3 | 5.93 (H-2), 5.14 (H-1), 5.12 (H-1), 1.22 (H-9/10) | - |
| 4 | - | 203.1 | 1.22 (H-9/10) | - |
| 5 | 6.24 [1H], s | 120.5 | 2.12 (H-8), 1.88 (H-7) | - |
| 6 | - | 156.0 | 2.12 (H-8), 1.88 (H-7) | - |
| 7 | 1.88 [3H], s | 27.9 | 2.12 (H-8) | - |
| 8 | 2.12 [3H], s | 20.9 | 1.88 (H-7) | - |
| 9 | 1.22 [3H], s | 23.6 | 5.93 (H-2), 1.22 (H-10) | - |
| 10 | 1.22 [3H], s | 23.6 | 5.93 (H-2), 1.22 (H-9) | - |

*Artemisia ketone (****42****)* was purified from fraction B by HPLC (2% EtOAc/hexane) yielding HPLC fraction 10 (*R_t_* 12.1 mins). Colourless oil.

(*E*)-7-Hydroxy-2,7-dimethylocta-2,5-dien-4-one (**43**) (novel as a natural product^1^)

NMR data for (*E*)-7-Hydroxy-2,7-dimethylocta-2,5-dien-4-one (**43**) in CDCl_3_ solution

| **Position** | **δ_H_ (ppm)** | **δ_C_ (ppm)** | **HMBC** | **^1^H-^1^H COSY** |
| --- | --- | --- | --- | --- |
| 1 | 1.94 [3H], s | 27.9 | 2.18 (H-10) | - |
| 2 | - | 156.8 | 2.18 (H-10), 1.94 (H-1) | - |
| 3 | 6.25 [1H], s | 123.2 | 2.18 (H-10), 1.94 (H-1) | - |
| 4 | - | 190.6 | 6.86 (H-6), 6.32 (H-5) | - |
| 5 | 6.32 [1H], d, *J* = 16 Hz | 127.2 | - | 6.86 (H-6) |
| 6 | 6.86 [1H], d, *J* = 16 Hz | 151.1 | 1.39 (H-8/9) | 6.32 (H-5) |
| 7 | - | 70.8 | 6.86 (H-6), 6.32 (H-5), 1.39 (H-8/9) | - |
| 8 | 1.39 [3H], s | 29.4 | 6.86 (H-6), 1.39 (H-9) | - |
| 9 | 1.39 [3H], s | 29.4 | 6.86 (H-6), 1.39 (H-8) | - |
| 10 | 2.18 [3H], s | 21.1 | 1.94 (H-1) | - |

*(E)-7-Hydroxy-2,7-dimethylocta-2,5-dien-4-one (****43****)* was purified from fraction J by HPLC (25% EtOAc/hexane/0.5% AcOH) yielding HPLC fraction 20 (*R_t_* 23.9 mins). Colourless oil. HRAPCIMS (*m/z*): 169.1224, Δ = -0.7 ppm for [M+H]^+^ (calc. 169.1223 for C_10_H_17_O_2_).

1. Novel as a natural product, but previously reported as a product from synthetic organic chemistry: V. A. Smit; S. L. Kireev; O. M. Nefedov; V. A. Tarasov. “Methylenecyclopropane as an alkene component in the Khand-Pauson reaction”. *Tetrahedron Letters* **30**(30), 4021-4024 (1989).

(*E*)-7-Hydroperoxy-2,7-dimethylocta-2,5-dien-4-one (**44**) (novel)

NMR data for (*E*)-7-Hydroperoxy-2,7-dimethylocta-2,5-dien-4-one (**44**) in CDCl_3_ solution

| **Position** | **δ_H_ (ppm)** | **δ_C_ (ppm)** | **HMBC** | **^1^H-^1^H COSY** |
| --- | --- | --- | --- | --- |
| 1 | 1.94 [3H], d, *J* = 1 Hz | 28.0 | 6.26 (H-3), 2.18 (H-10) | 6.26 (H-3) |
| 2 | - | 157.3 | 2.18 (H-10), 1.94 (H-1) | - |
| 3 | 6.26 [1H], qq, *J* = 1, 1 Hz | 123.2 | 6.27 (H-5), 2.18 (H-10), 1.94 (H-1) | 2.18 (H-10), 1.94 (H-1) |
| 4 | - | 190.1 | 6.84 (H-6), 6.27 (H-5) | - |
| 5 | 6.27 [1H], d, *J* = 16 Hz | 130.8 | 6.84 (H-6) | 6.84 (H-6) |
| 6 | 6.84 [1H], d, *J* = 16 Hz | 147.9 | 6.27 (H-5), 1.40 (H-8/9) | 6.27 (H-5) |
| 7 | 7.65 v br s [1H], (-OOH) | 82.3 | 6.84 (H-6), 6.27 (H-5), 1.40 (H-8/9) | - |
| 8 | 1.40 [3H], s | 24.1 | 1.40 (H-9) | - |
| 9 | 1.40 [3H], s | 24.1 | 1.40 (H-8) | - |
| 10 | 2.18 [3H], d, *J* = 1 Hz | 21.1 | 6.26 (H-3), 1.94 (H-1) | 6.26 (H-3) |

*(E)-7-Hydroperoxy-2,7-dimethylocta-2,5-dien-4-one (****44****)* was purified from fraction H by HPLC (15% EtOAc/hexane/0.5% AcOH) yielding HPLC fraction 23 (*R_t_* 20.9 mins). Colourless oil. ESI-MS (*m/z*): 185.1172, ∆ = 0.1 ppm for [M + H^+^] (calc. for C_10_H_17_O_3_); R_t_ 4.85 mins.

1. *Sesquiterpenes*

*trans*-β-Farnesene (**2**) (known) – see Supplementary List 1 (Artemis) for NMR data

*trans-β-Farnesene* (**2**) was purified from fraction A by HPLC (100% hexane) yielding HPLC fraction 6 (*R_t_* 9.6 mins). Colourless oil.

6,7-Epoxy-6,7-dihydro-β-farnesene (**45**) (novel as a natural product^1^)

NMR data for 6,7-epoxy-6,7-dihydro-β-farnesene (**45**) in CDCl_3_ solution

| **Position** | **δ_H_ (ppm)** | **δ_C_ (ppm)** | **HMBC** | **^1^H-^1^H COSY** |
| --- | --- | --- | --- | --- |
| 1 | 5.24 [1H], d, *J* = 17 Hz | 113.6 | 6.39 (H-2), 2.42 (H-4), 2.31 (H-4) | 6.39 (H-2) |
|  | 5.09 [1H], d, *J* = 11 Hz |  |  |  |
| 2 | 6.39 [1H], dd, *J* = 17, 11 Hz | 138.6 | 5.06 (H-15), 5.04 (H-15), 2.42 (H-4), 2.31 (H-4) | 5.24 (H-1), 5.09 (H-1) |
| 3 | - | 145.5 | 6.39 (H-2), 5.24 (H-1), 5.06 (H-15), 2.42 (H-4), 2.31 (H-4) | - |
| 4 | 2.42 [1H], ddd, *J* = 15, 10, 6  Hz | 28.1 | 5.06 (H-15), 5.04 (H-15) | 1.76 (H-5), 1.72 (H-5) |
|  | 2.31 [1H], ddd, *J* = 15, 9, 6 Hz |  |  |  |
| 5 | 1.76 [1H], m | 27.4 | - | 2.77 (H-6), 2.42 (H-4), 2.31 (H-4) |
|  | 1.72 [1H], m |  |  |  |
| 6 | 2.77 [1H], t, *J* = 6 Hz | 63.4 | 1.25 (H-14) | 1.76 (H-5), 1.72 (H-5) |
| 7 | - | 61.0 | 1.25 (H-14) | - |
| 8 | 1.66 [1H], m | 38.8 | 1.25 (H-14) | - |
|  | 1.43 [1H], ddd, *J* = 16, 9, 7 Hz |  |  |  |
| 9 | 2.08 [2H], m | 23.9 | - | 5.09 (H-10) |
| 10 | 5.09 [1H], t, *J* = 6 Hz | 123.7 | 1.69 (H-12), 1.61 (H-13) | 2.08 (H-9) |
| 11 | - | 132.0 | 1.69 (H-12), 1.61 (H-13) | - |
| 12 | 1.69 [3H], s | 25.8 | 1.61 (H-13) | - |
| 13 | 1.61 [3H], s | 17.7 | 1.69 (H-12) | - |
| 14 | 1.25 [3H], s | 16.6 | - | - |
| 15 | 5.06 [1H], s | 116.2 | - | - |
|  | 5.04 [1H], s |  |  |  |

*6,7-Epoxy-6,7-dihydro-β-farnesene* *(****45****)* was purified from fraction B by HPLC (2% EtOAc/hexane) yielding HPLC fraction 16 (*R_t_* 16.8 mins). Colourless oil.

1. *6,7-Epoxy-6,7-dihydro-β-farnesene* (**45**) is novel as a natural product, but has previously been reported as a product from synthetic organic chemistry: W. K. Giersch; A. F. Boschung; R. L. Snowden; K. H. Schulte-Elte “Thermoconversion of caryophyllene to farnesene-type sesquiterpenes. Short access to the enantiomers of (6*RS*, 7*RS*)- and (6*RS*, 7*SR*)-6,7-epoxy-6,7-dihydro-β-farnesenes”. *Helvetica Chimica Acta* **77**(1), 36-40 (1994).

*Caryophyllene / Humulane sesquiterpenes*

α-Humulene (**46**) (known)

NMR data for α-humulene (**46**) in CDCl_3_ solution

| **Position** | **δ_H_ (ppm)** | **δ_C_ (ppm)** | **HMBC** | **^1^H-^1^H COSY** |
| --- | --- | --- | --- | --- |
| 1 | 1.91 [2H], m | 41.9 | 5.16 (H-10), 1.07 (H-12/13) | 4.87 (H-2) |
| 2 | 4.87 [1H], t, *J* = 7 Hz | 125.0 | 1.44 (H-15) | 1.91 (H-1) |
| 3 | - | 133.2 | 1.44 (H-15) | - |
| 4 | 2.08 [2H], m | 39.8 | 1.44 (H-15) | - |
| 5 | 2.10 [2H], m | 23.3 | - | 4.95 (H-6) |
| 6 | 4.95 [1H], t, *J* = 7 Hz | 125.8 | 2.52 (H-8), 1.64 (H-14) | 2.10 (H-5) |
| 7 | - | 139.3 | 1.64 (H-14) | - |
| 8 | 2.52 [2H], d, *J* = 7 Hz | 40.4 | 5.16 (H-10), 1.64 (H-14) | 5.60 (H-9) |
| 9 | 5.60 [1H], dt, *J* = 16, 7 Hz | 127.7 | 2.52 (H-8) | 5.16 (H-10), 2.52 (H-8) |
| 10 | 5.16 [1H], d, *J* = 16 Hz | 141.0 | 2.52 (H-8), 1.07 (H-12/13) | 5.60 (H-9) |
| 11 | - | 37.4 | 5.60 (H-9), 5.16 (H-10), 1.07 (H-12/13) | - |
| 12 | 1.07 [3H], s | 26.7 | 1.07 (H-13) | - |
| 13 | 1.07 [3H], s | 26.7 | 1.07 (H-12) | - |
| 14 | 1.64 [3H], s | 17.9 | - | - |
| 15 | 1.44 [3H], s | 15.1 | - | - |

*α-Humulene (****46****)* was purified from fraction A by HPLC (100% hexane) yielding HPLC fraction 7 (*R_t_* 10.2 mins). Colourless oil. GC-MS (*m/z*): 204.2 for [M^+^] (C_15_H_24_); R_t_ 10.23 mins.

γ-Humulene (**47**) (known)

NMR data for γ-humulene (**47**) in CDCl_3_ solution

| **Position** | **δ_H_ (ppm)** | **δ_C_ (ppm)** | **HMBC** | **^1^H-^1^H COSY** |
| --- | --- | --- | --- | --- |
| 1 | 5.57 [1H], d, *J* = 17 Hz | 143.5 | 0.96 (H-12/13) | 5.86 (H-2) |
| 2 | 5.86 [1H], d, *J* = 17 Hz | 125.1 | 4.80 (H-15), 2.33 (H-4) | 5.57 (H-1) |
| 3 | - | 148.9 | 5.86 (H-2), 5.57 (H-1), 2.33 (H-4) | - |
| 4 | 2.33 [2H], m | 31.5 | 5.86 (H-2), 4.87 (H-15), 4.80 (H-15), 2.17 (H-5) | 2.17 (H-5) |
| 5 | 2.17 [2H], m | 30.4 | - | 2.33 (H-4), 5.28 (H-6) |
| 6 | 5.28 [1H], t, *J* = 7 Hz | 126.2 | 2.33 (H-4), 1.92 (H-8) | 2.17 (H-5) |
| 7 | - | 136.2 | 1.92 (H-8), 1.43 (H-14) | - |
| 8 | 1.92 [2H], m | 42.2 | 1.43 (H-14) | - |
| 9 | 1.40 [2H], m | 21.2 | - | - |
| 10 | 1.46 [2H], m | 45.8 | 5.57 (H-1), 1.92 (H-8), 0.96 (H-12/13) | - |
| 11 | - | 35.9 | 5.86 (H-2), 5.57 (H-1), 0.96 (H-12/13) | - |
| 12 | 0.96 [3H], s | 26.6 | 5.57 (H-1), 0.96 (H-13) | - |
| 13 | 0.96 [3H], s | 26.6 | 5.57 (H-1), 0.96 (H-12) | - |
| 14 | 1.43 [3H], s | 16.8 | - | - |
| 15 | 4.87 [1H], m | 113.1 | 5.86 (H-2), 2.33 (H-4) | - |
|  | 4.80 [1H], m |  |  |  |

*γ-Humulene (****47****)* was purified from fraction A by HPLC (100% hexane) yielding HPLC fraction 5 (*R_t_* 9.2 mins). Colourless oil.

6-Hydroxy-γ-humulene (**48**) (6-hydroxy-1,2(*trans*),3(15),7(14)-humulatriene) (novel)

NMR data for 6-hydroxy-1,2(*trans*),3(15),7(14)-humulatriene (**48**) in CDCl_3_ solution

| **Position** | **δ_H_ (ppm)** | **δ_C_ (ppm)** | **HMBC** | **^1^H-^1^H COSY** |
| --- | --- | --- | --- | --- |
| 1 | 5.49 [1H], d, *J* = 17 Hz | 141.1 | 2.28 (H-4), 2.41 (H-4), 0.97 (H-13), 0.95 (H-12) | 5.92 (H-2) |
| 2 | 5.92 [1H], d, *J* = 17 Hz | 127.4 | 4.96 (H-15), 4.95 (H-15), 2.41 (H-4), 2.28 (H-4) | 5.49 (H-1) |
| 3 | - | 146.8 | 5.92 (H-2), 5.49 (H-1) | - |
| 4 | 2.41 [1H], m  2.28 [1H], m | 27.2 | 5.92 (H-2) | - |
| 5 | 2.28 [1H], m  1.94 [1H], m | 30.7 | - | 4.24 (H-6) |
| 6 | 4.24 [1H], d, *J* = 12, 3 Hz | 82.4 | 5.26 (H-14), 5.20 (H-14) | 5.26 (H-14), 2.28 (H-5), 1.94 (H-5) |
| 7 | - | 143.1 | 5.26 (H-14), 5.20 (H-14) | - |
| 8 | 2.43 [1H], m  1.85 [1H], ddd *J* = 13, 13, 4 Hz | 35.8 | 5.26 (H-14), 5.20 (H-14) | 5.20 (H-14), 1.55 (H-9) |
| 9 | 1.55 [1H], m  1.17 [1H], m | 19.7 | - | 1.85 (H-8) |
| 10 | 1.55 [1H], m | 40.7 | 5.49 (H-1), 0.97 (H-13), 0.95 (H-12) | - |
|  | 1.16 [1H], m |  |  |  |
| 11 | - | 36.9 | 5.92 (H-2), 5.49 (H-1), 0.97 (H-13), 0.95 (H-12) | - |
| 12 | 0.95 [3H], s | 22.7 | 5.49 (H-1), 0.97 (H-13) | - |
| 13 | 0.97 [3H], s | 29.8 | 5.49 (H-1), 0.95 (H-13) | - |
| 14 | 5.26 [1H], s | 116.3 | - | 4.24 (H-6), 2.43 (H-8) |
|  | 5.20 [1H], s |  |  |  |
| 15 | 4.95 [1H], s | 114.3 | 5.92 (H-2), 2.41 (H-4), 2.28 (H-4) | - |
|  | 4.96 [1H], s |  |  |  |

*6-Hydroxy-γ-humulene (****48****)* was purified from fraction E by HPLC (7% EtOAc/hexane) yielding HPLC fraction 21 (*R_t_* 21.0 mins). Colourless oil.

β-Caryophyllene **(49**) (known)

NMR data for β-caryophyllene (**49**) in CDCl_3_ solution

| **Position** | **δ_H_ (ppm)** | **δ_C_ (ppm)** | **HMBC** |
| --- | --- | --- | --- |
| 1 | 2.42 [1H], m | 40.4 | 0.99 (H-12), 0.98 (H-13) |
|  | 1.65 [1H], m |  |  |
| 2 | 2.33 [1H], m | 48.5 | 4.94 (H-15) |
| 3 | - | 154.7 | 4.94 (H-15), 4.82 (H-15), 2.33 (H-2), 2.20 (H-5) |
| 4 | 2.35 [1H], m | 28.4 | 4.94 (H-15) |
|  | 2.00 [1H], m |  |  |
| 5 | 2.20 [1H], m | 34.7 | - |
|  | 2.00 [1H], m |  |  |
| 6 | 5.31 [1H], d, *J* = 7 Hz | 124.3 | 2.35 (H-4), 2.20 (H-5), 1.92 (H-8), 1.61 (H-14) |
| 7 | - | 135.6 | 1.92 (H-8), 1.61 (H-14) |
| 8 | 2.08 [1H], m | 40.0 | 1.61 (H-14) |
|  | 1.92 [1H], m |  |  |
| 9 | 1.52 [1H], m | 29.3 | - |
|  | 1.45 [1H], m |  |  |
| 10 | 1.69 [1H], m | 53.6 | 0.99 (H-12), 0.98 (H-13) |
| 11 | - | 33.0 | 0.99 (H-12), 0.98 (H-13) |
| 12 | 0.99 [1H], m | 30.1 | 0.98 (H-13) |
| 13 | 0.98 [1H], m | 22.6 | 0.99 (H-12) |
| 14 | 1.61 [3H], s | 16.3 | - |
| 15 | 4.94 [3H], br s | 111.7 | - |
|  | 4.82 [3H], br s |  |  |

*β-Caryophyllene* ***(49****)* was purified from fraction A by HPLC (100% hexane) yielding HPLC fraction 4 (*R_t_* 9.0 mins). Colourless oil.

Caryophyllene oxide **(50**) (known)

NMR data for caryophyllene oxide (**50**) in CDCl_3_ solution

| **Position** | **δ_H_ (ppm)** | **δ_C_ (ppm)** | **HMBC** |
| --- | --- | --- | --- |
| 1 | 1.68 [1H], dd, *J* = 10, 9 Hz | 39.7 | 1.01 (H-12), 0.98 (H-13) |
|  | 1.63 [1H], m |  |  |
| 2 | 2.62 [1H], ddd, *J* = 9, 9, 9 Hz | 48.7 | 4.98 (H-15) |
| 3 | - | 151.8 | 4.86 (H-15) |
| 4 | 2.34 [1H], ddd, *J* = 13, 8, 4 Hz | 29.6 | - |
|  | 2.12 [1H], ddd, *J* = 13, 8, 4 Hz |  |  |
| 5 | 1.65 [1H] m | 27.2 | - |
|  | 1.43 [1H] m |  |  |
| 6 | 2.88 [1H], dd, *J* = 10, 4 Hz | 63.8 | 1.21 (H-14) |
| 7 | - | 59.9 | 1.21 (H-14) |
| 8 | 2.10 [1H], ddd, *J* = 12, 12, 3 Hz | 39.1 | 1.21 (H-14) |
|  | 0.97 [1H], m |  |  |
| 9 | 2.25 [1H], ddd, *J* = 12, 8, 4 Hz | 30.2 | - |
|  | 1.32 [1H], m |  |  |
| 10 | 1.76 [1H], dd, *J* = 10, 9 Hz | 50.7 | 1.01 (H-12), 0.98 (H-13) |
| 11 | - | 34.0 | 1.01 (H-12), 0.98 (H-13) |
| 12 | 1.01 [3H], s | 21.6 | - |
| 13 | 0.98 [3H], s | 29.9 | - |
| 14 | 1.21 [3H], s | 17.0 | - |
| 15 | 4.98 [1H], s | 112.8 | - |
|  | 4.86 [1H], s |  |  |

*Caryophyllene oxide* ***(50****)* was purified from fraction D by HPLC (5% EtOAc/hexane) yielding HPLC fraction 10 (*R_t_* 15.8 mins). Colourless oil. ESI-MS (*m/z*): 221.1900, ∆ = 0.3 ppm for [M + H^+^] (calc. for C_15_H_25_O); R_t_ 8.92 mins.

*Eudesmane sesquiterpenes*

β-Selinene (**51**) (4(15),11-eudesmadiene) (known^1,2^)

NMR data for β-selinene (**51**) in CDCl_3_ solution

| **Position** | **δ_H_ (ppm)** | **δ_C_ (ppm)** | **HMBC** | **^1^H-^1^H COSY** |
| --- | --- | --- | --- | --- |
| 1 | - | 36.0 | 0.73 (H-14) | - |
| 2 | 1.44 [1H], m | 41.9 | 0.73 (H-14) | - |
|  | 1.28 [1H], m |  |  |  |
| 3 | 1.61 [2H], m | 23.5 | 2.01 (H-4), 1.28 (H-2) | 2.01 (H-4) |
| 4 | 2.32 [1H], dddd, *J* = 12, 4, 4, 2 Hz | 36.9 | 4.71 (H-15), 4.44 (H-15) | 4.71 (H-15), 4.44 (H-15), 1.61 (H-3) |
|  | 2.01 [1H], m |  |  |  |
| 5 | - | 151.0 | 4.44 (H-15), 1.29 (H-7) | - |
| 6 | 1.82 [1H], d, *J* = 13 Hz | 49.9 | 4.71 (H-15), 4.44 (H-15), 1.97 (H-8), 1.29 (H-7), 0.73 (H-14) | 4.71 (H-15), 4.44 (H-15) |
| 7 | 1.58 [1H], m | 29.5 | 1.97 (H-8) | - |
|  | 1.29 [1H], m |  |  |  |
| 8 | 1.97 [1H], m | 45.8 | 4.72 (H-13), 4.69 (H-13), 1.75 (H-12), 1.29 (H-7) | - |
| 9 | 1.56 [1H], m | 26.8 | 1.97 (H-8), 1.29 (H-7), 1.26 (H-10) | - |
|  | 1.46 [1H], m |  |  |  |
| 10 | 1.51 [1H], m | 41.1 | 1.97 (H-8), 0.73 (H-14) | - |
|  | 1.26 [1H], m |  |  |  |
| 11 | - | 150.9 | 4.72 (H-13), 4.69 (H-13), 1.75 (H-12) | - |
| 12 | 1.75 [3H], s | 21.1 | 4.72 (H-13), 4.69 (H-13), 1.97 (H-8) | 4.72 (H-13), 4.69 (H-13) |
| 13 | 4.72 [1H], br s | 108.1 | 1.97 (H-8), 1.75 (H-12) | 1.75 (H-12) |
|  | 4.69 [1H], br s |  |  |  |
| 14 | 0.73 [3H], s | 16.3 | 1.28 (H-2) | - |
| 15 | 4.71 [1H], br s | 105.4 | - | 2.01 (H-4), 1.82 (H-6) |
|  | 4.44 [1H],br s |  |  |  |

*β-Selinene (****51****)* was purified from fraction A by HPLC (100% hexane) yielding HPLC fraction 3 (*R_t_* 8.5 mins). Colourless oil. GC-MS (*m/z*): 204.1 for [M + H^+^] (C_15_H_24_); R_t_ 10.37 mins.

1. L.-K. Sy; G. D. Brown “Three sesquiterpenes from *Artemisia annua*”. *Phytochemistry* **48**(7) 1207-1211 (1998).
2. G. D. Brown; G.-Y. Liang; L.-K. Sy “Terpenoids from the seeds of *Artemisia annua*”. *Phytochemistry* **64** 303-323 (2003).

*Cadinane / Amorphane sesquiterpenes*

Artemisinic acid (**9**) (known^1^) – see Supplementary List 1 (Artemis) for NMR data

*Artemisinic acid* (**9**) was purified from fraction G by HPLC (10% EtOAc/hexane/0.5% AcOH) yielding HPLC fraction 5 (*R_t_* 10.1 mins). Colourless oil.

1. G. D. Brown; L.-K. Sy "*In vivo* transformations of artemisinic acid in *Artemisia annua* plants" *Tetrahedron* **63** 9548-9566 (2007).

7α-Hydroxy-isoartemisinic acid (**52**) (novel)

NMR data for 7α-hydroxy-isoartemisinic acid (**52**) in CDCl_3_ solution

| **Position** | **δ_H_ (ppm)** | **δ_C_ (ppm)** | **HMBC** | **^1^H-^1^H COSY** |
| --- | --- | --- | --- | --- |
| 1 | 1.47 [1H], m | 42.2 | 0.98 (H-14) | - |
| 2 | 1.92 [1H], m | 21.1 | - | - |
|  | 1.65 [1H], m |  |  |  |
| 3 | 5.34 [1H], d *J* = 7 Hz | 118.8 | 1.73 (H-15) | 1.73 (H-15), 2.11 (H-5) |
| 4 | - | 146.2 | 1.73 (H-15) | - |
| 5 | 2.11 [1H], m | 31.1 | 1.73 (H-15) | 5.34 (H-5) |
|  | 2.05 [1H], m |  |  |  |
| 6 | 1.32 [1H], m | 44.4 | - | - |
| 7 | — | 76.6 | 6.21 (H-13), 5.72 (H-13) | - |
| 8 | 1.35 [1H], m | 22.7 | - | - |
|  | 1.31 [1H], m |  |  |  |
| 9 | 1.84 [1H], m | 35.2 | 0.98 (H-14) | - |
|  | 1.75 [1H], m |  |  |  |
| 10 | 1.59 [1H], m | 29.4 | 0.98 (H-14) | 0.98 (H-14) |
| 11 | - | 145.4 | 6.21 (H-13), 5.72 (H-13) | - |
| 12 | - | 168.7 | 6.21 (H-13), 5.72 (H-13) | - |
| 13 | 6.21 [1H], s | 119.6 | - | - |
|  | 5.72 [1H], s |  |  |  |
| 14 | 0.98 [3H], d, *J* = 7 Hz | 19.5 | - | 1.59 (H-10) |
| 15 | 1.73 [3H], s | 23.7 | - | 5.34 (H-3) |

*7α-Hydroxy-isoartemisinic acid (****52****)* was purified from fraction I by HPLC (25% EtOAc/ hexane/ 0.5% AcOH) yielding HPLC fraction 11 (*R_t_* 17.5 mins). Colourless oil.

Artemisinic acid methyl ester (**53**) (known^1^)

NMR data for artemisinic acid methyl ester (**53**) in CDCl_3_ solution

| **Position** | **δ_H_ (ppm)** | **δ_C_ (ppm)** | **HMBC** | **^1^H-^1^H COSY** |
| --- | --- | --- | --- | --- |
| 1 | 1.43 [1H], m | 41.3 | 0.90 (H-14) | - |
| 2 | 1.92 [1H], br d, *J* = 14 Hz | 25.6 | - | - |
|  | 1.54 [1H], m |  |  |  |
| 3 | 1.87 [1H], m | 26.4 | - | - |
|  | 1.76 [1H], m |  |  |  |
| 4 | - | 134.9 | 1.92 (H-2), 1.59 (H-15) | - |
| 5 | 4.99 [1H], br | 120.3 | 2.72 (H-7), 1.87 (H-3), 1.76 (H-3), 1.59 (H-15) | 2.53 (H-6), 1.87 (H-3), 1.76 (H-3), 1.59 (H-15) |
| 6 | 2.53 [1H], br | 37.9 | - | 4.99 (H-5), 2.72 (H-7), 1.87 (H-3), 1.76 (H-3), 1.59 (H-15) |
| 7 | 2.72 [1H], d, *J* = 12 Hz | 42.3 | - | 6.28 (H-13), 5.43 (H-13), 2.53 (H-6), 1.40 (H-8), 1.34 (H-8) |
| 8 | 1.40 [1H], m | 25.9 | - | - |
|  | 1.34 [1H], m |  |  |  |
| 9 | 1.71 [1H], m | 35.2 | 0.90 (H-14) | - |
|  | 1.07 [1H], m |  |  |  |
| 10 | 1.43 [1H], m | 27.6 | 0.90 (H-14) | 0.90 (H-14) |
| 11 | - | 143.3 | 6.28 (H-13), 2.72 (H-7) | - |
| 12 | - | 167.9 | 6.28 (H-13), 5.43 (H-13), 3.74 (CO_2_Me), 2.72 (H-7) | - |
| 13 | 6.28 [1H], s | 124.2 | 2.72 (H-7) | 2.72 (H-7) |
|  | 5.43 [1H], s |  |  |  |
| 14 | 0.90 [3H], d, *J* = 6 Hz | 19.8 | - | 1.43 (H-10) |
| 15 | 1.59 [3H], s | 23.7 | - | 4.99 (H-5), 2.53 (H-6) |
| CO_2_Me | 3.74 [3H], s | 51.8 | - | - |

*Artemisinic acid methyl ester (****53****)* was purified from fraction B by HPLC (2% EtOAc/hexane) yielding HPLC fraction 9 (*R_t_* 11.8 mins). Colurless oil. HRAPCIMS (*m/z*): 249.1855, Δ = -2.3 ppm for [M+H]^+^ (calc. 249.1849 for C_16_H_25_O_2_).

1. L.-K. Sy; G. D. Brown “Three sesquiterpenes from *Artemisia annua*”. *Phytochemistry* **48**(7) 1207-1211 (1998).

Arteannuin R (**54**) (novel)

NMR data for arteannuin R (**54**) in CDCl_3_ solution

| **Position** | **δ_H_ (ppm)** | **δ_C_ (ppm)** | **HMBC** | **^1^H-^1^H COSY** |
| --- | --- | --- | --- | --- |
| 1 | 1.37 [1H], m | 43.2 | 0.88 (H-14) | 2.14 (H-10) |
| 2 | 2.09 [1H], dddd, *J* = 13, 3, 3, 3 Hz | 29.3 | - | 2.42 (H-3), 1.94 (H-3) |
|  | 1.33 [1H], m |  |  |  |
| 3 | 2.42 [1H], br dd, *J* = 13, 13 Hz | 25.5 | 4.72 (H-15), 4.66 (H-15), 4.10 (H-5), 1.37 (H-1) | 4.73 (H-15), 4.66 (H-15), 2.09 (H-2), 1.33 (H-2) |
|  | 1.94 [1H], m |  |  |  |
| 4 | - | 151.5 | 4.72 (H-15), 4.66 (H-15), 4.10 (H-5), 2.42 (H-3), 1.94 (H-3) | - |
| 5 | 4.10 [1H], d, *J* = 3 Hz | 74.9 | 4.72 (H-15), 4.66 (H-15) | 1.95 (H-6) |
| 6 | 1.95 [1H], m | 43.0 | 4.10 (H-5) | 4.10 (H-5), 2.78 (H-7) |
| 7 | 2.78 [1H], ddd, *J* = 13, 9, 5 Hz | 42.1 | 6.50 (H-13), 5.90 (H-13), 2.34 (H-8) | 6.50 (H-13), 5.90 (H-13), 2.34 (H-8), 1.95 (H-6), 1.44 (H-8) |
| 8 | 2.34 [1H], dddd, *J* = 13, 13, 13, 5 Hz | 27.4 | - | 2.78 (H-7), 1.86 (H-9), 1.10 (H-9) |
|  | 1.44 [1H], dddd, *J* = 13, 3, 3, 3 Hz |  |  |  |
| 9 | 1.86 [1H], m | 35.8 | 0.88 (H-14) | 2.34 (H-8), 2.14 (H-10), 1.44 (H-8) |
|  | 1.10 [1H], dddd, *J* = 13, 13, 13, 4 Hz |  |  |  |
| 10 | 2.14 [1H], m | 29.5 | 0.88 (H-14) | 1.37 (H-1), 1.10 (H-9), 0.88 (H-14) |
| 11 | - | 142.0 | 6.50 (H-13), 2.78 (H-7) | - |
| 12 | - | 171.9 | 6.50 (H-13), 5.90 (H-13), 2.78 (H-7) | - |
| 13 | 6.50 [1H], s | 127.3 | 2.78 (H-7) | 2.78 (H-7) |
|  | 5.90 [1H], s |  |  |  |
| 14 | 0.88 [3H], d, *J* = 7 Hz | 20.7 | - | 2.14 (H-10) |
| 15 | 4.72 [1H], s | 109.0 | 4.10 (H-5), 2.42 (H-3), 1.94 (H-3) | 2.42 (H-3) |
|  | 4.66 [1H], s |  |  |  |

*Arteannuin R (****54****)* was purified from fraction I by HPLC (25% EtOAc/hexane/0.5% AcOH) yielding HPLC fraction 12 (*R_t_* 18.1 mins). Colourless oil. HRAPCIMS (*m/z*): 233.1539, Δ = -1.2 ppm for [M + H]^+^ (calc. 233.1536 for C_15_H_21_O_2_).

Arteannuin S (**55**) (novel)

NMR data for arteannuin S (**55**) in CDCl_3_ solution

| **Position** | **δ_H_ (ppm)** | | **δ_C_ (ppm)** | **HMBC** | **^1^H-^1^H COSY** |
| --- | --- | --- | --- | --- | --- |
| 1 | 1.49 [1H], m |  | 43.5 | 4.66 (H-5), 0.85 (H-14) | - |
| 2 | 1.89 [1H], m |  | 26.7 | - | 2.22 (H-3), 2.11 (H-3) |
|  | 1.47 [1H], m |  |  |  |  |
| 3 | 2.22 [1H], m |  | 27.1 | 4.93 (H-15), 4.84 (H-15) | 1.89 (H-2), 1.47 (H-2) |
|  | 2.11 [1H], m |  |  |  |  |
| 4 | - |  | 145.9 | 4.93 (H-15), 2.22 (H-3), 2.11 (H-3) | - |
| 5 | 4.66 [1H], d *J* = 9 | Hz | 84.8 | 4.93 (H-15), 4.84 (H-15), 2.22 (H-3), 2.17 (H-6), 2.11 (H-3) | 2.17 (H-6) |
| 6 | 2.17 [1H], m |  | 43.2 | 2.66 (H-7) | 4.66 (H-5), 2.66 (H-7) |
| 7 | 2.66 [1H], ddd, *J* = 12, 4, 4 Hz |  | 41.5 | 6.19 (H-13), 5.71 (H-13), 4.66  (H-5) | 2.17 (H-6) |
| 8 | 1.74 [1H], m |  | 25.2 | - | - |
|  | 1.70 [1H], m |  |  |  |  |
| 9 | 1.86 [1H], m |  | 35.3 | 0.85 (H-14) | - |
|  | 1.07 [1H], m |  |  |  |  |
| 10 | 1.56 [1H], m |  | 29.8 | 0.85 (H-14) | 0.85 (H-14) |
| 11 | - |  | 142.9 | 6.19 (H-13) | - |
| 12 | - |  | 169.0 | 6.19 (H-13), 5.71 (H-13) | - |
| 13 | 6.19 [1H], s |  | 123.6 | - | - |
|  | 5.71 [1H], s |  |  |  |  |
| 14 | 0.85 [3H], s |  | 20.0 | - | 1.56 (H-10) |
| 15 | 4.93 [1H], s |  | 111.2 | 4.66 (H-5), 2.22 (H-3), 2.11 (H-3) | - |
|  | 4.84 [1H], s |  |  |  |  |

*Arteannuin S (****55****)* was purified from fraction K by HPLC (40% EtOAc/hexane/1% AcOH) yielding HPLC fraction 9 (*R_t_* 10.5 mins). Colourless oil. HRAPCIMS (*m/z*): 249.1488, Δ = -1.1 ppm for [M + H]^+^ (calc. 249.1485 for C_15_H_21_O_3_).

Artannuin S (**55**) is the 11,13-dehydro analogue of Arteannuin H and may share a parallel biogenesis (*i.e.* *via* the secondary allylic hydroperoxide obtained from autoxidation of artemisinic acid (AA), which is an alternative to the tertiary allylic hydroperoxide (AA-OOH) that is proposed to be involved in the biogenesis of several other natural products that appear in Supplementary list 2).

α-Epoxy-artemisinic acid **(56**) (known^1^)

NMR data for α-epoxy-artemisinic acid (**56**) in CDCl_3_ solution

| **Position** | **δ_H_ (ppm)** | **δ_C_ (ppm)** | **HMBC** | **^1^H-^1^H COSY** |
| --- | --- | --- | --- | --- |
| 1 | 1.24 [1H], m | 39.9 | 0.89 (H-14) | - |
| 2 | 1.63 [1H], m | 21.9 | - | 1.80 (H-3) |
|  | 1.31 [1H], m |  |  |  |
| 3 | 1.80 [1H], m | 24.7 | 1.29 (H-15) | 1.63 (H-2), 1.31 (H-2) |
|  | 1.64 [1H], m |  |  |  |
| 4 | - | 57.8 | 1.80 (H-3), 1.29 (H-15) | - |
| 5 | 2.54 [1H], s | 59.2 | 1.80 (H-3), 1.29 (H-15) | - |
| 6 | 2.17 [1H], br | 38.7 | 2.54 (H-5) | 2.76 (H-7) |
| 7 | 2.76 [1H], d, *J* = 12 Hz | 41.2 | 6.47 (H-13), 5.64 (H-13), 2.54 (H-5) | 2.17 (H-6), 1.63 (H-8), 1.33 (H-8) |
| 8 | 1.63 [1H], m | 27.1 | - | 2.76 (H-7) |
|  | 1.33 [1H], dddd, *J* = 13, 13, 13, 4 Hz |  |  |  |
| 9 | 1.79 [1H], m | 34.8 | 0.89 (H-14) | - |
|  | 1.11 [1H], m |  |  |  |
| 10 | 1.24 [1H], m | 29.4 | 0.89 (H-14) | 0.89 (H-14) |
| 11 | - | 141.8 | 6.47 (H-13), 2.76 (H-7) | - |
| 12 | - | 170.2 | 6.47 (H-13), 5.64 (H-13) | - |
| 13 | 6.47 [1H], s | 126.7 | - |  |
|  | 5.64 [1H], s |  |  |  |
| 14 | 0.89 [3H], d, *J* = 6 Hz | 19.1 | - | 1.24 (H-10) |
| 15 | 1.29 [3H], s | 23.5 | - | - |

*α-Epoxy-artemisinic acid* ***(56****)* was purified from fraction G by HPLC (10% EtOAc/hexane/0.5% AcOH) yielding HPLC fraction 20 (*R_t_* 27.0 mins). Colourless oil. ESI-MS (*m/z*): 251.1642, ∆ = 0.6 ppm for [M + H^+^] (calc. for C_15_H_23_O_3_); R_t_ 6.50 mins. HRAPCIMS (*m/z*): 251.1646, Δ = -1.9 ppm for [M + H]^+^ (calc. 251.1642 for C_13_H_23_O_3_).

1.G. D. Brown; G.-Y. Liang; L.-K. Sy “Terpenoids from the seeds of *Artemisia annua*” *Phytochemistry* **64** 303-323 (2003).4α, 5α-Epoxy-6α-hydroxyartemisinic acid methyl ester (**57**) (novel as a natural product^1^)

NMR data for 4α, 5α-epoxy-6α-hydroxyartemisinic acid methyl ester (**57**) in CDCl_3_ solution

| **Position** | **δ_H_ (ppm)** | **δ_C_ (ppm)** | **HMBC** | **^1^H-^1^H COSY** |
| --- | --- | --- | --- | --- |
| 1 | 1.15 [1H], m | 48.2 | 2.91 (H-5), 1.83 (H-3), 0.91 (H-14) | - |
| 2 | 1.58 [1H], m | 15.0 | - | - |
|  | 1.40 [1H], m |  |  |  |
| 3 | 1.83 [1H], dd, *J* = 14, 6 Hz | 24.5 | 1.34 (H-15) | - |
|  | 1.66 [1H], m |  |  |  |
| 4 | - | 60.9 | 1.34 (H-15) | - |
| 5 | 2.91 [1H], s | 60.4 | 1.83 (H-3), 1.34 (H-15) | - |
| 6 | 3.00 [1H] br s (6-OH) | 69.3 | 2.91 (H-5) | - |
| 7 | 3.16 [1H], dd, *J* = 14, 3 Hz | 46.5 | 5.39 (H-13), 3.03 (6-OH), 2.91 (H-5) | 5.39 (H-13), 1.71 (H-8), 1.43 (H-8) |
| 8 | 1.71 [1H], m | 27.1 | - | 3.16 (H-7) |
|  | 1.43 [1H], m |  |  |  |
| 9 | 1.75 [1H], m | 34.3 | 0.91 (H-14) | - |
|  | 1.22 [1H], m |  |  |  |
| 10 | 1.25 [1H], m | 30.9 | 0.91 (H-14) | 0.91 (H-14) |
| 11 | - | 143.1 | 6.09 (H-13) | - |
| 12 | - | 169.9 | 6.09 (H-13), 5.39 (H-13), 3.76 (CO_2_Me) | - |
| 13 | 6.09 [1H], s | 122.6 | - | 3.16 (H-7) |
|  | 5.39 [1H], s |  |  |  |
| 14 | 0.91 [3H], d, *J* = 7 Hz | 19.0 | - | 1.25 (H-10) |
| 15 | 1.34 [3H], s | 23.4 | - | - |
| CO_2_Me | 3.76 [3H], s | 52.0 | - | - |

*4α, 5α-Epoxy-6α-hydroxyartemisinic acid methyl ester (****57****)* was purified from fraction H by HPLC (15% EtOAc/hexane/0.5% AcOH) yielding HPLC fraction 26 (*R_t_* 23.9 mins); and from fraction I by HPLC (25% EtOAc/hexane/0.5% AcOH) yielding HPLC fraction 8 (*R_t_* 14.0 mins). Colourless oil. HRAPCIMS (*m/z*): 281.1749, Δ = -0.4 ppm for [M + H]^+^ (calc. 281.1747 for C_16_H_25_O_4_).

1. *4α, 5α-Epoxy-6α-hydroxyartemisinic acid methyl ester (****57***) is novel as a natural product, but has been previously obtained as a product from the chemical reduction of arteannuin B (no NMR data was reported): A.K. Bhattacharya; D. C. Jain; R. P. Sharma. “Selective reduction of the exomethylene group of α-methylene γ- or δ-lactones with CdCl_2_-Mg-MeOH-H_2_O” *Journal of Chemical Research, Synopses* (12), 768-769. (1998).

Deoxyarteannuin B (**58**) (known^1^)

NMR data for deoxyarteannuin B (**58**) in CDCl_3_ solution

| **Position** | **δ_H_ (ppm)** | **δ_C_ (ppm)** | **HMBC** | **^1^H-^1^H COSY** |
| --- | --- | --- | --- | --- |
| 1 | 1.66 [1H], m | 44.7 | 1.00 (H-14) | - |
| 2 | 2.05 [1H], dddd, *J* = 13, 11, 8, 4 Hz | 19.8 | - | 1.94 (H-3) |
|  | 1.85 [1H], dddd, *J* = 13, 4, 2, 2 Hz |  |  |  |
| 3 | 1.94 [2H], m | 26.1 | 1.67 (H-15) | 5.13 (H-5), 2.05 (H-2) |
| 4 | - | 142.5 | 1.67 (H-15) | - |
| 5 | 5.13 [1H], m | 118.8 | 1.67 (H-15) | 1.94 (H-3), 1.67 (H-15) |
| 6 | - | 84.5 | - | - |
| 7 | 2.66 [1H], dd, *J* = 13, 4 Hz | 52.5 | 6.10 (H-13), 5.37 (H-13) | 5.37 (H-13), 1.36 (H-8) |
| 8 | 1.93 [1H], m | 22.1 | - | 2.66 (H-7), 1.81 (H-9), 1.18 (H-9) |
|  | 1.36 [1H], dddd, *J* = 13, 13, 13, 4 Hz |  |  |  |
| 9 | 1.81 [1H], br d, *J* = 12 Hz | 34.3 | 1.00 (H-14) | 1.93 (H-8), 1.55 (H-10), 1.36 (H-8) |
|  | 1.18 [1H], dddd, *J* = 13, 12, 12, 4 Hz |  |  |  |
| 10 | 1.55 [1H], m | 30.6 | 1.00 (H-14) | 1.18 (H-9), 1.00 (H-14) |
| 11 | - | 139.8 | 6.10 (H-13) | - |
| 12 | - | 171.0 | 6.10 (H-13) | - |
| 13 | 6.10 [1H], d, *J* = 3 Hz | 117.0 | - | 2.66 (H-7) |
|  | 5.37 [1H], d, *J* = 3 Hz |  |  |  |
| 14 | 1.00 [3H], d, *J* = 7 Hz | 19.9 | - | 1.55 (H-10) |
| 15 | 1.67 [3H], s | 23.6 | - | 5.13 (H-5) |

*Deoxyarteannuin B (****58****)* was purified from fraction G by HPLC (10% EtOAc/hexane/0.5% AcOH) yielding HPLC fraction 8 (*R_t_* 13.1 mins). Colourless oil. ESI-MS (*m/z*): 233.1536, ∆ = 0.2 ppm for [M + H^+^] (calc. for C_15_H_21_O_2_); R_t_ 7.58 mins. HRAPCIMS (*m/z*): 233.1537, Δ = -0.3 ppm for [M + H]^+^ (calc. 233.1536 for C_15_H_21_O_2_).

1. L.-K. Sy; G. D. Brown "Deoxyarteannuin B, dihydro-deoxyarteannuin B and *trans*-5-hydroxy-2-isopropenyl-5-methlhex-3-en-1-ol from *Artemisia annua*". *Phytochemistry* **58** 1159-1166 (2001).

Dihydro-*epi*-deoxyarteannuin B (**12**) (known^1-3^) – see Supplementary List 1 (Artemis) for NMR data

Dihydro-*epi*-deoxyarteannuin B (**12**) was purified from fraction E by HPLC (7% EtOAc/hexane) yielding HPLC fraction 20 (*R_t_* 19.0 mins). Colourless oil. ESI-MS (m/z): 235.1693, C_15_H_23_O_2_ [M + H^+^], ∆ = 0.3 ppm (R_t_ 7.68 mins).

1. G. D. Brown. "Two new compounds from *Artemisia annua*". *J. Nat. Prod.* **55** 1756-1770 (1992).
2. L.-K. Sy; G. D. Brown, “A novel endoperoxide and related sesquiterpenes from *Artemisia annua* which are possibly derived from allylic hydroperoxides”. *Tetrahedron* **54** 4345-4356 (1998).
3. L.-K. Sy; G. D. Brown "Deoxyarteannuin B, dihydro-deoxyarteannuin B and *trans*-5-hydroxy-2-isopropenyl-5-methlhex-3-en-1-ol from *Artemisia annua*". *Phytochemistry* **58** 1159-1166 (2001).

*epi*-Deoxyarteannuin B (**13**) (known^1,2^) – see Supplementary List 1 (Artemis) for NMR data

*epi*-Deoxyarteannuin B (**13**) was purified from fraction E by HPLC (7% EtOAc/hexane) yielding HPLC fraction 18 (*R_t_* 16.2 mins); and from fraction G by HPLC (10% EtOAc/hexane/0.5% AcOH) yielding HPLC fraction 7 (*R_t_* 12.6 mins). Colourless oil. ESI-MS (*m/z*): 233.1536, C_15_H_21_O_2_ [M + H^+^], ∆ = 0.3 ppm. (R_t_ 7.63 mins).

1.G. D. Brown. "Two new compounds from *Artemisia annua*". *J. Nat. Prod.* **55** 1756-1770 (1992).

2. L.-K. Sy; G. D. Brown "Deoxyarteannuin B, dihydro-deoxyarteannuin B and *trans*-5-hydroxy-2-isopropenyl-5-methylhex-3-en-1-ol from *Artemisia annua*" *Phytochemistry* **58** 1159-1166 (2001).

Dehydroarteannuin L (**59**) (novel)

NMR data for dehydroarteannuin L (**59**) in CDCl_3_ solution

| **Position** | **δ_H_ (ppm)** | **δ_C_ (ppm)** | **HMBC** | **^1^H-^1^H COSY** |
| --- | --- | --- | --- | --- |
| 1 | 1.72 [1H], m | 40.0 | 3.72 (H-5), 0.96 (H-14) | - |
| 2 | 1.86 [1H], m | 25.3 | - | 2.39 (H-3), 2.28 (H-3) |
|  | 1.47 [1H], m |  |  |  |
| 3 | 2.39 [1H], m | 29.3 | 4.95 (H-15), 4.91 (H-15), 3.72 (H-5) | 1.86 (H-2), 1.47 (H-2) |
|  | 2.28 [1H], m |  |  |  |
| 4 | - | 145.6 | 4.91 (H-15) | - |
| 5 | 3.72 [1H], s | 76.4 | 4.91 (H-15) | - |
| 6 | — | 85.7 | 3.72 (H-5) | - |
| 7 | 3.18 [1H], dd, *J* = 8, 8 Hz | 41.1 | 6.18 (H-13), 5.59 (H-13) | 6.18 (H-13), 5.59 (H-13), 1.92 (H-8), 1.38 (H-8) |
| 8 | 1.92 [1H], m | 29.0 | - | 3.18 (H-7) |
|  | 1.38 [1H], m |  |  |  |
| 9 | 1.62 [1H], m | 30.9 | 0.96 (H-14) | - |
|  | 1.14 [1H], m |  |  |  |
| 10 | 1.43 [1H], m | 29.6 | 0.96 (H-14) | 0.96 (H-14) |
| 11 | - | 141.7 | 6.18 (H-13) | - |
| 12 | - | 170.1 | 6.18 (H-13), 5.59 (H-13) | - |
| 13 | 6.18 [1H], s | 121.2 | - | 3.18 (H-7) |
|  | 5.59 [1H], s |  |  |  |
| 14 | 0.96 [3H], d, *J* = 7 Hz | 20.1 | - | 1.43 (H-10) |
| 15 | 4.95 [1H], s | 114.4 | 3.72 (H-5) | - |
|  | 4.91 [1H], s |  |  |  |

*Dehydroarteannuin L (****59****)* was purified from fraction I by HPLC (25% EtOAc/hexane/0.5% AcOH) yielding HPLC fraction 11 (*R_t_* 17.2 mins).

Arteannuin B (**60**) (known)

NMR data for arteannuin B (**60**) in CDCl_3_ solution

| **Position** | **δ_H_ (ppm)** | **δ_C_ (ppm)** | **HMBC** | **^1^H-^1^H COSY** |
| --- | --- | --- | --- | --- |
| 1 | 1.49 [1H], m | 43.5 | 0.99 (H-14) | - |
| 2 | 1.74 [1H], ddd, *J* = 12, 12, 4 Hz | 16.2 | - | - |
|  | 1.53 [1H], brd, *J* = 12 Hz |  |  |  |
| 3 | 1.92 [1H], dd, *J* = 13, 4 Hz | 24.3 | 1.34 (H-15) | - |
|  | 1.71 [1H], ddd, *J* = 13, 13, 6 Hz |  |  |  |
| 4 | - | 58.4 | 1.34 (H-15) | - |
| 5 | 2.69 [1H], s | 58.6 | 1.34 (H-15) | - |
| 6 | - | 81.1 | 2.74 (H-7), 2.69 (H-5),  2.07 (H-8), 1.41 (H-8) | - |
| 7 | 2.74 [1H], dddd, *J* = 12, 3, 3, 3 Hz | 52.6 | 6.15 (H-13) | 6.15 (H-13), 5.45 (H-13), 2.07 (H-8), 1.41 (H-8) |
| 8 | 2.07 [1H], dddd, *J* = 12, 3, 3, 3 Hz | 21.6 | - | 2.74 (H-7), 1.89 (H-9), 1.25 (H-9) |
|  | 1.41 [1H], dddd, *J* = 12, 12, 12, 4 Hz |  |  |  |
| 9 | 1.89 [1H], dddd, *J* = 13, 3, 3, 3 Hz | 33.8 | 0.99 (H-14) | 2.07 (H-8) |
|  | 1.25 [1H], m |  |  |  |
| 10 | 1.49 [1H], m | 30.6 | 0.99 (H-14) | 0.99 (H-14) |
| 11 | - | 138.5 | 6.15 (H-13), 2.74 (H-7) | - |
| 12 | - | 170.0 | 6.15 (H-13), 5.45 (H-13) | - |
| 13 | 6.15 [1H], d, *J* = 3 Hz | 117.7 | 2.74 (H-7) | 2.74 (H-7) |
|  | 5.45 [1H], d, *J* = 3 Hz |  |  |  |
| 14 | 0.99 [3H], d, *J* = 6 Hz | 18.5 | - | 1.49 (H-10) |
| 15 | 1.34 [3H], s | 22.7 | - | - |

*Arteannuin B (****60****)* was purified from fraction I by HPLC (25% EtOAc/hexane/0.5% AcOH) yielding HPLC fraction 10 (*R_t_* 15.5 mins). Colourless oil. ESI-MS (*m/z*): 249.1486, ∆ = 0.4 ppm for [M + H^+^] (calc. for C_15_H_21_O_3_); R_t_ 6.17 mins. HRAPCIMS (*m/z*): 249.1488, Δ = -0.9 ppm for [M + H]^+^ (calc. 249.1485 for C_15_H_21_O_3_).

Dehydroarteannuin M (**61**) (known^1^)

NMR data for dehydroarteannuin M (**61**) in CDCl_3_ solution

| **Position** | **δ_H_ (ppm)** | **δ_C_ (ppm)** | **HMBC** | **^1^H-^1^H COSY** |
| --- | --- | --- | --- | --- |
| 1 | 1.55 [1H], m | 39.7 | 3.11 (H-5), 0.95 (H-14) | - |
| 2 | 1.77 [1H], m | 22.4 | 1.74 (H-3) | - |
|  | 1.36 [1H], m |  |  |  |
| 3 | 1.74 [1H], m | 34.3 | 3.11 (H-5), 1.43 (H-15) | - |
|  | 1.64 [1H], m |  |  |  |
| 4 | - | 72.6 | 3.11 (H-5), 1.74 (H-3), 1.43 (H-15) | - |
| 5 | 3.11 [1H], s | 77.1 | 1.43 (H-15) | - |
| 6 | — | 86.0 | 3.11 (H-5) | - |
| 7 | 3.25 [1H], dd, *J* = 7, 7 Hz | 41.7 | 6.18 (H-13), 5.59 (H-13) | 1.90 (H-8), 1.36 (H-8) |
| 8 | 1.90 [1H], m | 28.7 | - | 3.25 (H-7) |
|  | 1.36 [1H], m |  |  |  |
| 9 | 1.60 [1H], m | 30.3 | 0.95 (H-14) | - |
|  | 1.12 [1H], m |  |  |  |
| 10 | 1.45 [1H], m | 28.6 | 0.95 (H-14) | 0.95 (H-14) |
| 11 | - | 141.7 | 6.18 (H-13), 3.25 (H-7), 1.90 (H-8),  1.36 (H-8) | - |
| 12 | - | 170.1 | 6.18 (H-13), 5.59 (H-13), 3.25 (H-7) | - |
| 13 | 6.18 [1H], s | 121.4 | - | - |
|  | 5.59 [1H], s |  |  |  |
| 14 | 0.95 [3H], d, *J* = 6 Hz | 20.3 | - | 1.45 (H-10) |
| 15 | 1.43 [3H], s | 26.2 | 1.74 (H-3) | - |

*Dehydroarteannuin M (****61****)* was purified from fraction L by HPLC (40% EtOAc/hexane/1% AcOH) yielding HPLC fraction 19 (*R_t_* 22.5 mins). Colourless oil. ESI-MS (*m/z*): 267.1591, ∆ = 0.5 ppm for [M + H^+^] (calc. for C_15_H_23_O_4_); R_t_ 5.08 mins. HRAPCIMS (*m/z*): 267.1591, Δ = 0.1 ppm for [M + H]^+^ (calc. 267.1591 for C_15_H_23_O_4_). The stereochemistry at the 5- and 6-positions was assigned from NOESYcorrelations; and the stereochemistry at the 4-position by analogy with arteannuin M.

1. This metabolite was previously reported as “dihydroxycadinanolide” with undefined stereochemistry in: G. D. Brown. "Cadinanes from *Artemisia annua* that may be intermediates in the biosynthesis of artemisinin". *Phytochemistry*  **36** 637-641 (1994). Note that the assignments at positions 1-, 2-,3-, 7- and 9- have also been modified from the original in the above Table.

Annulide (**62**) (known ^1,2^)

NMR data for annulide (**62**) in CDCl_3_ solution

| **Position** | **δ_H_ (ppm)** | **δ_C_ (ppm)** | **HMBC** | **^1^H-^1^H COSY** |
| --- | --- | --- | --- | --- |
| 1 | 1.55 [1H], m | 42.7 | 2.02 (H-6), 1.45 (H-2), 0.92 (H-14) | - |
| 2 | 2.03 [1H], m | 28.7 | - | 2.27 (H-3), 2.18 (H-3) |
|  | 1.45 [1H], ddd, *J* = 14, 4, 4 Hz |  |  |  |
| 3 | 2.27 [1H], m | 29.3 | 5.20 (H-15), 4.88 (H-15) | 2.03 (H-2), 1.45 (H-2) |
|  | 2.18 [1H], m |  |  |  |
| 4 | - | 145.4 | 5.20 (H-15), 5.03 (H-5) | - |
| 5 | 5.03 [1H], d, *J* = 12 Hz | 75.6 | 5.20 (H-15), 4.88 (H-15), 2.66 (H-7), 2.27 (H-3), 2.02 (H-6) | 2.02 (H-6) |
| 6 | 2.02 [1H], m | 43.2 | 2.66 (H-7) | 5.03 (H-5), 2.66 (H-7) |
| 7 | 2.66 [1H], ddd, *J* = 11, 5, 5 Hz | 40.3 | 6.52 (H-13), 5.60 (H-13), 2.02 (H-6) | 6.53 (H-13), 5.60 (H-13), 2.02 (H-6), 1.68 (H-8) |
| 8 | 1.68 [2H], m | 30.4 | - | 2.66 (H-7), 1.17 (H-9) |
| 9 | 1.85 [1H], dddd, *J* = 13, 4, 4, 4 Hz | 34.8 | 0.92 (H-14) | 1.68 (H-8) |
|  | 1.17 [1H], dddd, *J* = 12, 12, 12, 4 Hz |  |  |  |
| 10 | 1.69 [1H], m | 27.9 | 0.92 (H-14) | 0.92 (H-14) |
| 11 | - | 139.0 | 6.52 (H-13), 2.66 (H-7) | - |
| 12 | - | 165.1 | 6.52 (H-13), 5.60 (H-13), 2.66 (H-7) | - |
| 13 | 6.52 [1H], s | 129.2 | 2.66 (H-7) | 2.66 (H-7) |
|  | 5.60 [1H], s |  |  |  |
| 14 | 0.92 [3H], d, *J* = 7 Hz | 20.1 | - | 1.69 (H-10) |
| 15 | 5.20 [1H], s | 106.5 | - | - |
|  | 4.88 [1H],s |  |  |  |

*Annulide (****62****)* was purified from fraction F by HPLC (8% EtOAc/hexane/0.5% AcOH) yielding HPLC fraction 18 (*R_t_* 18.6 mins); difficult to separate from isoannulide (**63**). Colourless oil. ESI-MS (*m/z*): 233.1536, ∆ = 0.2 ppm for [M + H^+^] (calc. for C_15_H_21_O_2_); R_t_ 7.51 mins. HRAPCIMS (*m/z*): 233.1538, Δ = -0.6 ppm for [M + H]^+^ (calc. 233.1536 for C_15_H_21_O_2_).

1. G.D. Brown. "Annulide, a sesquiterpene lactone from *Artemisia annua*". *Phytochemistry* **32** 391-393 (1993).

2. L.-K. Sy; G.D. Brown "Deoxyarteannuin B, dihydro-deoxyarteannuin B and *trans*-5-hydroxy-2-isopropenyl-5-methylhex-3-en-1-ol from *Artemisia annua*" *Phytochemistry* **58** 1159-1166 (2001).

Isoannulide (**63**) (known^1,2^)

NMR data for isoannulide (**63**) in CDCl_3_ solution

| **Position** | **δ_H_ (ppm)** | **δ_C_ (ppm)** | **HMBC** |
| --- | --- | --- | --- |
| 1 | 1.52 [1H], m | 40.8 | 0.89 (H-14) |
| 2 | 2.22 [2H],m | 26.8 | - |
| 3 | 5.47 [1H], br s | 124.1 | 2.22 (H-2), 1.82 (H-15) |
| 4 | - | 130.4 | 1.82 (H-15) |
| 5 | 4.88 [1H], d, *J* = 12 Hz | 74.8 | 2.20 (H-6), 1.82 (H-15) |
| 6 | 2.20 [1H], m | 39.4 | - |
| 7 | 2.68 [1H], ddd, *J* = 12, 5, 5 Hz | 38.5 | 6.53 (H-13) |
| 8 | 1.79 [1H], m | 31.9 | - |
|  | 1.69 [1H], m |  |  |
| 9 | 1.78 [1H], m | 34.8 | 0.89 (H-14) |
|  | 1.16 [1H], m |  |  |
| 10 | 1.31 [1H], m | 28.9 | 0.89 (H-14) |
| 11 | - | 139.9 | 6.53 (H-13) |
| 12 | - | 166.8 | 6.53 (H-13), 5.63 (H-13) |
| 13 | 6.53 [1H], br s | 129.4 | - |
|  | 5.63 [1H], br s |  |  |
| 14 | 0.89 [3H], d, *J* = 6 Hz | 20.2 | - |
| 15 | 1.82 [3H], s | 18.5 | - |

*Isoannulide (****63****)* was purified from fraction F by HPLC (8% EtOAc/hexane/0.5% AcOH) yielding HPLC fraction 18 (*R_t_* 18.6 mins); difficult to separate from annulide (**62**). Colourless oil. HRAPCIMS (*m/z*) poor chromatographic separated from annulide (**62**) (*m/z*): 233.1538, Δ = -0.6 ppm for [M + H]^+^ (calc. 233.1536 for C_15_H_21_O_2_).

1. G.D. Brown. "Annulide, a sesquiterpene lactone from *Artemisia annua*". *Phytochemistry* **32** 391-393 (1993).
2. L.-K. Sy; G.D. Brown "Deoxyarteannuin B, dihydro-deoxyarteannuin B and *trans*-5-hydroxy-2-isopropenyl-5-methlhex-3-en-1-ol from *Artemisia annua*" *Phytochemistry* **58** 1159-1166 (2001).

*epi*-11-Hydroxy-arteannuin I (**64**) (novel)

NMR data for *epi*-11-hydroxy-arteannuin I (**64**) in CDCl_3_ solution

| **Position** | **δ_H_ (ppm)** | **δ_C_ (ppm)** | **HMBC** | **^1^H-^1^H COSY** |
| --- | --- | --- | --- | --- |
| 1 | 1.65 [1H], m | 41.2 | 0.92 (H-14) | - |
| 2 | 1.95 [1H], m | 28.5 | - | 2.24 (H-3), 2.02 (H-3) |
|  | 1.47 [1H], m |  |  |  |
| 3 | 2.24 [1H], m | 29.6 | 4.82 (H-15) | 1.95 (H-2), 1.47 (H-2) |
|  | 2.02 [1H], m |  |  |  |
| 4 | - | 148.2 | 4.51 (H-5) | - |
| 5 | 4.51 [1H], d, *J* = 12 Hz | 79.0 | 4.82 (H-15) | 1.81 (H-6) |
| 6 | 1.81 [1H], m | 49.6 | 4.51 (H-5), 1.47 (H-2) | 4.51 (H-5), 2.56 (H-7) |
| 7 | 2.56 [1H], ddd, *J* = 12, 7, 6 Hz | 44.5 | 1.47 (H-13) | 1.81 (H-6), 1.72 (H-8), 1.51 (H-8) |
| 8 | 1.72 [1H], m | 22.6 | - | 2.56 (H-7) |
|  | 1.51 [1H], m |  |  |  |
| 9 | 1.79 [1H], m | 34.1 | 0.92 (H-14) | - |
|  | 1.05 [1H], m |  |  |  |
| 10 | 1.55 [1H], m | 28.8 | 0.92 (H-14) | 0.92 (H-14) |
| 11 | - | 87.2 | 1.47 (H-13) | - |
| 12 | - | 176.5 | 1.47 (H-13) | - |
| 13 | 1.47 [3H], s | 19.4 | - | - |
| 14 | 0.92 [3H], d, *J* = 6 Hz | 20.0 | - | 1.55 (H-10) |
| 15 | 4.82 [1H], s | 101.4 | 4.51 (H-5) | - |
|  | 4.78 [1H], s |  |  |  |

*epi-11-Hydroxy-arteannuin I (****64****)* was purified from fraction K by HPLC (40% EtOAc/hexane/1% AcOH) yielding HPLC fraction 4 (*R_t_* 8.0 mins). Colourless oil. ESI-MS (*m/z*): 251.1642, ∆ = 0.4 ppm for [M + H^+^] (calc. for C_15_H_23_O_3_); R_t_ 6.79 mins. HRAPCIMS (*m/z*): 251.1645, Δ = -1.2 ppm for [M + H]^+^ (calc. 251.1642 for C_15_H_23_O_3_).

Arteannuin H (**21**) (known^1,2^) – see Supplementary List 1 (Artemis) for NMR data

Arteannuin H (**21**) was purified from fraction B by HPLC (2% EtOAc/hexane) yielding HPLC fraction 17 (*R_t_* 17.9 mins). Colourless oil.

1.L.-K. Sy and G. D. Brown, “A Novel Endoperoxide and Related Sesquiterpenes from *Artemisia annua* which are Possibly Derived from Allylic Hydroperoxides”, *Tetrahedron* **54** 4345-4356 (1998).

2.L.-K. Sy, K.-S. Ngo and G.D. Brown "Biomimetic synthesis of arteannuin H and the 3,2-rearrangement of allylic hydropeoxides" *Tetrahedron* **55** 15127-15140 (1999).

Artemisinic acid 6α-peroxy ester (**65**) (novel)

NMR data for artemisinic acid 6α-peroxy ester (**65**) in CDCl_3_ solution

| **Position** | **δ_H_ (ppm)** | **δ_C_ (ppm)** | **HMBC** | **^1^H-^1^H COSY** |
| --- | --- | --- | --- | --- |
| 1 | 1.87 [1H], m | 49.2 | 1.97 (H-2), 1.91 (H-9), 1.83  (H-3), 1.77 (H-2), 0.97 (H-14) | 2.07 (H-10) |
| 2 | 1.97 [1H], m | 21.2 | 1.83 (H-3) | 2.36 (H-3), 1.87 (H-1) |
|  | 1.77 [1H], m |  |  |  |
| 3 | 2.36 [1H], br dd, *J* = 14, 12 Hz | 26.7 | 5.72 (H-5), 1.59 (H-15) | 1.97 (H-2), 1.77 (H-2) |
|  | 1.83 [1H], m |  |  |  |
| 4 | - | 123.1 | 1.97 (H-2), 1.77 (H-2), 1.59 (H-15) | - |
| 5 | 5.72 [1H], br s | 135.4 | 1.83 (H-3), 1.59 (H-15) | 1.59 (H-15) |
| 6 | - | 106.5 | 2.63 (H-7), 1.97 (H-2), 1.92 (H-8), 1.87 (H-1), 1.76 (H-8) | - |
| 7 | 2.63 [1H], dddd, *J* = 12, 3, 3, 3 Hz | 52.6 | 6.13 (H-13), 5.41 (H-13), 1.91 (H-9), 1.76 (H-8), 1.23 (H-9) | 6.13 (H-13), 5.41 (H-13), 1.92 (H-8), 1.76 (H-8) |
| 8 | 1.92 [1H], m | 22.1 | - | 2.63 (H-7) |
|  | 1.76 [1H], m |  |  |  |
| 9 | 1.91 [1H], m | 34.0 | 2.63 (H-7), 2.07 (H-10), 1.76 (H-8), 0.97 (H-14) | 2.07 (H-10) |
|  | 1.23 [1H], m |  |  |  |
| 10 | 2.07 [1H], m | 31.0 | 1.97 (H-2), 1.91 (H-9), 1.23  (H-9), 0.97 (H-14) | 1.87 (H-1), 1.23 (H-9), 0.97 (H-14) |
| 11 | - | 138.1 | 6.13 (H-13), 2.63 (H-7), 1.76 (H-8) | — |
| 12 | - | 170.3 | 6.13 (H-13), 5.41 (H-13) | — |
| 13 | 6.13 [1H], d, *J* = 3 Hz | 118.2 | 2.63 (H-7) | 2.63 (H-7) |
|  | 5.41 [1H], d, *J* = 3 Hz |  |  |  |
| 14 | 0.97 [3H], d, *J* = 7 Hz | 18.4 | - | 2.07 (H-10) |
| 15 | 1.59 [3H], s | 21.1 | 5.72 (H-5) | 5.72 (H-5) |

*Artemisinic acid, 6α-peroxy ester (****65****)* was purified from fraction E by HPLC (7% EtOAc/hexane) yielding HPLC fraction 14 (*R_t_* 14.0 mins). ESI-MS (*m/z*): 249.1486, ∆ = 0.4 ppm for [M + H^+^] (calc. for C_15_H_21_O_3_); R_t_ 8.00 mins. Colourless oil. HRAPCIMS (*m/z*): 249.1488, Δ = -1.1 ppm for [M + H]^+^ (calc. 249.1485 for C_15_H_21_O_3_).

The stereochemistry of the peroxy substituent at the 6-position of (**65**) is consistent with a biogenesis by (3,2)-rearrangement of the tertiary allylic hydroperoxide from autoxidation of artemisinic acid (AA-OOH), which can then undergo esterification with the 12-carboxylic acid group.

*Seco-cadinane / amorphane sesquiterpenes*

Artemisinin (**22**) (known) – see Supplementary List 1 (Artemis) for NMR data

Artemisitene (**66**) (known^1^)

NMR data for artemisitene (**66**) in CDCl_3_ solution

| **Position** | **δ_H_ (ppm)** | **δ_C_ (ppm)** | **HMBC** | **^1^H-^1^H COSY** |
| --- | --- | --- | --- | --- |
| 1 | 1.45 [1H], m | 50.1 | 1.02 (H-14) | - |
| 2 | 1.97 [1H], dddd, *J* = 13, 5, 5, 4 Hz | 24.6 | - | 2.41 (H-3) |
|  | 1.50 [1H], m |  |  |  |
| 3 | 2.41 [1H], ddd, *J* = 14, 12, 4 Hz | 35.9 | 1.46 (H-15) | 1.97 (H-2), 1.50 (H-2) |
|  | 2.07 [1H], ddd, *J* = 14, 4, 3 Hz |  |  |  |
| 4 | - | 105.4 | 6.00 (H-5), 2.41 (H-3), 1.46 (H-15) | - |
| 5 | 6.00 [1H], m | 93.5 | 2.55 (H-7) | - |
| 6 | - | 79.4 | 6.00 (H-5), 2.55 (H-7) | - |
| 7 | 2.55 [1H], dd, *J* = 13, 5 Hz | 46.1 | 6.57 (H-13), 5.68 (H-13) | 1.77 (H-8), 1.57 (H-8) |
| 8 | 1.77 [1H], m | 31.6 | 2.55 (H-7) | 2.55 (H-7) |
|  | 1.57 [1H], dd, *J* = 14, 4 Hz |  |  |  |
| 9 | 1.76 [1H], m | 33.7 | 1.02 (H-14) | - |
|  | 1.21 [1H], m |  |  |  |
| 10 | 1.45 [1H], m | 37.8 | 1.02 (H-14) | 1.02 (H-14) |
| 11 | - | 134.9 | 6.57 (H-13), 2.55 (H-7) | - |
| 12 | - | 162.8 | 6.57 (H-13), 6.00 (H-5),  5.68 (H-13), 2.55 (H-7) | - |
| 13 | 6.57 [1H], s | 130.5 | 2.55 (H-7) | - |
|  | 5.68 [1H], s |  |  |  |
| 14 | 1.02 [3H], d, *J* = 6 Hz | 19.9 | - | 1.45 (H-10) |
| 15 | 1.46 [3H], s | 25.4 | - | - |

*Artemisitene (****66****)* was purifiied from fraction I by HPLC (25% EtOAc/hexane/0.5% AcOH) yielding HPLC fraction 9 (*R_t_* 15.2 mins) and from fraction J by HPLC (25% EtOAc/hexane/0.5% AcOH) yielding HPLC fraction 13 (*R_t_* 15.3 mins). Colourless oil. ESI-MS (*m/z*): 281.1384, ∆ = 0.7 ppm for [M + H^+^] (calc. for C_15_H_21_O_5_); R_t_ 6.37 mins. HRAPCIMS (*m/z*): 281.1391, Δ = -2.6 ppm for [M + H]^+^ (calc. 281.1384 for C_15_H_21_O_5_).

1. L.-K. Sy; G. D. Brown, "Synthesis of 6,7-dehydroartemisinic acid" *J. Chem. Soc.*, *Perkin Trans I* 2421-2429 (2001).

Deoxyartemistene (**67**) (novel as a natural product, but obtained previously by extensive chemical transformation of artemisinin^1^ – fully assigned ^1^H NMR reported below in Table for the first time)

NMR data for deoxyartemisitene (**67**) in CDCl_3_ solution

| **Position** | **δ_H_ (ppm)** | **δ_C_ (ppm)** | **HMBC** | **^1^H-^1^H COSY** |
| --- | --- | --- | --- | --- |
| 1 | 1.30 [1H], m | 44.4 | 0.96 (H-14) | - |
| 2 | 1.91 [1H], m | 22.0 | - | 2.36 (H-3) |
|  | 1.30 [1H], m |  |  |  |
| 3 | 2.36 [1H], m | 33.9 | 1.50 (H-15), 1.30 (H-2) | 1.91 (H-2), 1.30 (H-2) |
|  | 1.61 [1H], m |  |  |  |
| 4 | - | 109.9 | 1.50 (H-15) | - |
| 5 | 5.80 [1H], s | 99.6 | - | - |
| 6 | - | 82.4 | 5.80 (H-5) | - |
| 7 | 2.80 [1H], dd, *J* = 13, 4 Hz | 44.7 | 6.43 (H-13) | 1.77 (H-8), 1.51 (H-8) |
| 8 | 1.77 [1H], m | 30.9 | - | 2.80 (H-7) |
|  | 1.51 [1H], m |  |  |  |
| 9 | 1.79 [1H], m | 33.7 | 0.96 (H-14) | - |
|  | 1.22 [1H], m |  |  |  |
| 10 | 1.34 [1H], m | 35.6 | 0.96 (H-14) | 0.96 (H-14) |
| 11 | - | 135.3 | 6.43 (H-13) | - |
| 12 | - | 163.1 | 6.43 (H-13) | - |
| 13 | 6.43 [1H], s | 129.4 | - | - |
|  | 5.64 [1H], s |  |  |  |
| 14 | 0.96 [3H], d, *J* = 6 Hz | 18.6 | - | 1.34 (H-10) |
| 15 | 1.50 [3H], s | 24.1 | - | - |

*Deoxyartemistene (****67****)* was purified from fraction I by HPLC (25% EtOAc/hexane/0.5% AcOH) yielding HPLC fraction 5 (*R_t_* 12.8 mins) and from fraction J by HPLC (25% EtOAc/hexane/0.5% AcOH) yielding HPLC fraction 11 (*R_t_* 13.0 mins). Colourless oil. HRAPCIMS (*m/z*): 265.1438, Δ = -1.3 ppm for [M + H]^+^ (calc. 265.1434 for C_15_H_21_O_4_).

1. A. M. Galal; S. A. Ross; M. A. ElSohly; H. N. ElSohly; F. S. El-Feraly; M. S. Ahmed; A. T. McPhail. “Deoxyartemisinin derivatives from photooygenation of anhydrodeoxydihydroartemisinin and their cytotoxic evaluation”. *Journal of Natural Products* **65**(2), 184-188 (2002).

4,5-*seco*-4,5-Keto, aldehyde-amorphan-11,13-ene-12-oic acid (**68**) (known^1^)

NMR data for 4,5-*seco*-4,5-keto, aldehyde-amorphan-11,13-ene-12-oic acid (**68**) in CDCl_3_ solution

| **Posi-tion** | **δ_H_ (ppm)** | **δ_C_ (ppm)** | **HMBC** | **^1^H-^1^H COSY** |
| --- | --- | --- | --- | --- |
| 1 | 1.51 [1H], br | 41.8 | 1.88 (H-2), 1.80 (H-9), 1.21 (H-9), 0.96 (H-14) | - |
| 2 | 1.88 [1H], dd, *J* = 16, 12, 6 Hz | 23.2  br | - | 2.46 (H-3), 2,25 (H-3) |
|  | 1.51 [1H], br |  |  |  |
| 3 | 2.46 [1H], v br | 38.1 | 2.15 (H-15), 1.88 (H-2) | 1.88 (H-2) |
|  | 2.35 [1H], br |  |  |  |
| 4 | - | 208.8 br | 2.15 (H-15), 1.88 (H-2) | - |
| 5 | 9.30 [1H], v br | 204.2 v br | - | - |
| 6 | 2.25 [1H], v br | 57.5 br | - | - |
| 7 | 2.81 [1H], br | 39.6 br | 5.69 (H-13), 1.80 (H-9) | 5.69 (H-13) |
| 8 | 1.93 [1H], m | 31.8 br | - | - |
|  | 1.35 [1H], m |  |  |  |
| 9 | 1.80 [1H], d, *J* = 12 Hz | 34.8 | 0.96 (H-14) | 1.32 (H-10) |
|  | 1.21 [1H], ddd, *J* = 13, 12, 12 Hz |  |  |  |
| 10 | 1.32 [1H], br | 33.3 br | 1.80 (H-9), 0.96 (H-14) | 1.21 (H-9), 0.96 (H-14) |
| 11 | - | 141.4 br | 6.42 (H-13) | - |
| 12 | - | 170.9 br | 5.69 (H-13) | - |
| 13 | 6.42 [1H], br | 128.2 | - | 2.81 (H-7) |
|  | 5.69 [1H], s |  |  |  |
| 14 | 0.96 [3H], d, *J* = 6 Hz | 19.7 | 1.80 (H-9), 1.21 (H-9) | 1.32 (H-10) |
| 15 | 2.15 [3H], s | 30.0 | - | - |

*4,5-seco-4,5-keto, aldehyde-amorphan-11,13-ene-12-oic acid (****68****)* was purified from fraction L by HPLC (40% EtOAc/hexane/1% AcOH) yielding HPLC fraction 14 (*R_t_* 14.8 mins). Colourless oil. ESI-MS (*m/z*): 289.1410, ∆ = 0.5 ppm for [M + H^+^] (calc. for C_15_H_23_O_4_); R_t_ 5.24 mins.

1. G. D. Brown. "Cadinanes from *Artemisia annua* that may be intermediates in the biosynthesis of artemisinin". *Phytochemistry*  **36** 637-641 (1994).

Arteannuin T (**69**) (novel)

NMR data for arteannuin T (**69**) in CDCl_3_ solution

| **Position** | **δ_H_ (ppm)** | **δ_C_ (ppm)** | **HMBC** | **^1^H-^1^H COSY** |
| --- | --- | --- | --- | --- |
| 1 | 1.20 [1H], m | 42.8 | 0.95 (H-14) | - |
| 2 | 1.79 [2H], m | 22.6 | - | 2.47 (H-3), 2.37 (H-3) |
| 3 | 2.47 [1H], ddd *J* = 17, 8, 6 Hz | 39.2 | 2.24 (H-15) | 1.79 (H-2) |
|  | 2.37 [1H], ddd *J* = 17, 8, 6 Hz |  |  |  |
| 4 | - | 211.2 | 2.47 (H-3), 2.37 (H-3), 2.24 (H-15) | - |
| 5 | 5.90 [1H], d, *J* = 3 Hz | 102.9 | - | 1.48 (H-6) |
| 6 | 1.48 [1H], m | 43.8 | 5.90 (H-5) | 5.90 (H-5), 2.34 (H-7) |
| 7 | 2.34 [1H], m | 35.1 | 6.52 (H-13), 5.90 (H-5), 5.62 (H-13) | 1.48 (H-6) |
| 8 | 1.79 [2H], m | 22.6 | - | - |
| 9 | 1.77 [1H], m | 34.1 | 0.95 (H-14) | - |
|  | 1.13 [1H], m |  |  |  |
| 10 | 1.22 [1H], m | 34.5 | 0.95 (H-14) | 0.95 (H-14) |
| 11 | - | 137.9 | 6.52 (H-13) | - |
| 12 | - | 164.2 | 6.52 (H-13), 5.62 (H-13) | - |
| 13 | 6.52 [1H], s | 127.2 | - | - |
|  | 5.62 [1H], s |  |  |  |
| 14 | 0.95 [3H], d, *J*= 7 Hz | 19.5 | - | 1.22 (H-10) |
| 15 | 2.24 [3H], s | 30.2 | - | - |

*Arteannuin T (****69****)* was purified from fraction K by HPLC (40% EtOAc/hexane/1% AcOH) yielding HPLC fraction 10 (*R_t_* 11.2 mins). Colourless oil. ESI-MS (*m/z*): 267.1591, ∆ = 0.6 ppm for [M + H^+^] (calc. for C_15_H_23_O_4_); R_t_ 5.26 mins.

Arteannuin U (**70**) (novel)

NMR data for arteannuin U (**70**) in CDCl_3_ solution

| **Position** | **δ_H_ (ppm)** | **δ_C_ (ppm)** | **HMBC** | **^1^H-^1^H COSY** |
| --- | --- | --- | --- | --- |
| 1 | 0.94 [1H], m | 47.5 | 2.08 (H-2), 1.79 (H-9), 1.50 (H-2), 0.91 (H-14) | 2.08 (H-2), 1.50 (H-2), 1.38 (H-6) |
| 2 | 2.08 [1H], ddd, *J* = 12, 8, 6 Hz | 31.8 | 3.17 (H-3) | 3.17 (H-3), 0.94 (H-1) |
|  | 1.50 [1H], ddd, *J* = 12, 12, 9 Hz |  |  |  |
| 3 | 3.17 [1H], ddd, *J* = 9, 9, 6 Hz | 51.8 | 4.14 (H-5), 2.17 (H-15), 1.50 (H-2) | 4.14 (H-5), 2.08 (H-2), 1.50 (H-2) |
| 4 | - | 212.5 | 4.14 (H-5), 3.17 (H-3), 2.17 (H-15) | - |
| 5 | 4.14 [1H], dd, *J* = 9, 9 Hz | 77.5 | 3.17 (H-3), 2.08 (H-2) | 3.17 (H-3), 1.38 (H-6) |
| 6 | 1.38 [1H], m | 57.2 br | 4.14 (H-5), 3.17 (H-3), 2.08 (H-2), 0.94 (H-1) | 0.94 (H-1), 4.14 (H-5), 2.43 (H-7) |
| 7 | 2.43 [1H], ddd, *J* = 13, 13, 2 Hz | 42.7 v br | 6.23 (H-13), 5.25 (H-13), 4.14 (H-5) | 1.71 (H-8), 1.47 (H-8), 1.38 (H-6) |
| 8 | 1.71 [1H], dddd, *J* = 13, 3, 3, 3 Hz | 32.4 | - | 2.43 (H-7), 1.02 (H-9) |
|  | 1.47 [1H], dddd, *J* = 13, 13, 13, 3 Hz |  |  |  |
| 9 | 1.79 [1H], dddd, *J* = 13, 3, 3, 3 Hz | 35.1 | 0.91 (H-14) | 1.71 (H-8), 1.47 (H-8), 1.31 (H-10) |
|  | 1.02 [1H], dddd, *J* = 13, 13, 12, 3 Hz |  |  |  |
| 10 | 1.31 [1H], m | 37.5 | 1.79 (H-9), 1.50 (H-2), 0.91 (H-14) | 1.79 (H-9), 1.02 (H-9), 0.91 (H-14) |
| 11 | - | 143.5 | 6.23 (H-13), 5.65 (H-13) | - |
| 12 | - | 170.2 | 6.23 (H-13), 5.65 (H-13) | - |
| 13 | 6.23 [1H], s | 124.7 | - | - |
|  | 5.65 [1H], s |  |  |  |
| 14 | 0.91 [3H], d, *J* = 7 Hz | 19.8 | - | 1.31 (H-10) |
| 15 | 2.17 [3H], s | 31.5 | 3.17 (H-3) | - |

*Arteannuin U (****70****)* was purified from fraction M by HPLC (50% EtOAc/hexane/1% AcOH) yielding HPLC fraction 5 (*R_t_* 18.2 mins). Colourless oil. ESI-MS (*m/z*): 267.1591, ∆ = 0.6 ppm for [M + H^+^] (calc. for C_15_H_23_O_4_); R_t_ 5.20 mins. HRAPCIMS (*m/z*): 267.1596, Δ = -1.8 ppm for [M + H]^+^ (calc. 267.1591 for C_15_H_23_O_4_).

4,5-*seco*-4-Keto-5-carboxylic-amorphan-11,13-ene-12-oic acid (**71**) (known^1^)

NMR data for 4,5-*seco*-4-keto-5-carboxylic-amorphan-11,13-ene-12-oic acid (**71**) in CDCl_3_ solution

| **Position** | **δ_H_ (ppm)** | **δ_C_ (ppm)** | **HMBC** | **^1^H-^1^H COSY** |
| --- | --- | --- | --- | --- |
| 1 | 1.55 [1H], dddd, *J* = 11, 11, 3, 3 Hz | 43.8 | 2.50 (H-3), 2.37 (H-3), 2.26 (H-6), 0.94 (H-14) | 2.26 (H-6) |
| 2 | 1.85 [1H], dddd, *J* = 11, 13, 3, 3 Hz | 23.9 | 2.50 (H-3), 2.37 (H-3) | 2.50 (H-3), 2.37 (H-3) |
|  | 1.61 [1H], m |  |  |  |
| 3 | 2.50 [1H], ddd, *J* = 17, 11, 3 Hz | 38.4 | 2.14 (H-15) | 1.85 (H-2), 1.61 (H-2) |
|  | 2.37 [1H], ddd, *J* = 17, 8, 3 Hz |  |  |  |
| 4 | - | 209.1 | 2.50 (H-3), 2.37 (H-3), 2.14 (H-15), 1.85 (H-2) | - |
| 5 | - | 180.3 | 2.26 (H-6) | - |
| 6 | 2.26 [1H], dd, *J* = 11, 11 Hz | 54.0 | - | 1.55 (H-1), 2.88 (H-7) |
| 7 | 2.88 [1H], dd, *J* = 11, 11 Hz | 41.8 br | 6.38 (H-13), 5.75 (H-13), 2.26 (H-6) | 2.26 (H-6), 1.80 (H-8), 1.45 (H-8) |
| 8 | 1.80 [1H], m | 31.6 br | - | 2.88 (H-7) |
|  | 1.45 [1H], ddd, *J* = 12, 12, 11 Hz |  |  |  |
| 9 | 1.78 [1H], m | 34.8 | 0.94 (H-14) | - |
|  | 1.21 [1H], ddd, *J* = 12, 12, 11 Hz |  |  |  |
| 10 | 1.29 [1H], m | 33.5 | 0.94 (H-14) | 0.94 (H-14) |
| 11 | - | 142.6 | 6.38 (H-13) | - |
| 12 | - | 171.9 | 6.38 (H-13), 5.75 (H-13) | - |
| 13 | 6.38 [1H], s | 127.3 | - | - |
|  | 5.75 [1H], s |  |  |  |
| 14 | 0.94 [3H], d, *J* = 6 Hz | 19.6 | - | 1.29 (H-10) |
| 15 | 2.14 [3H], s | 30.0 | - | - |

*4,5-*seco*-4-Keto-5-carboxylic-amorphan-11,13-ene-12-oic acid (****71****)* was purified from fraction M by HPLC (50% EtOAc/hexane/1% AcOH) yielding HPLC fraction 12 (*R_t_* 14.0 mins). Colourless oil. ESI-MS (*m/z*): 283.1540, ∆ = 0.5 ppm for [M + H^+^] (calc. for C_15_H_23_O_5_); R_t_ 4.70 mins. HRAPCIMS (*m/z*): 283.1547, Δ = -2.5 ppm for [M + H]^+^ (calc. 283.1540 for C_15_H_23_O_5_).

1. H.-B. Li; Y. Yu; Z.-Z. Wang; J. Yang; W. Xiao; X.-S. Yao. “Two new sesquiterpenes from *Artemisia annua*”. *Magnetic Resonance in Chemistry* **53**(3), 244-247 (2015)

Arteannuin V (**72**) (novel)

NMR data for arteannuin V (**72**) in CDCl_3_ solution

| **Position** | **δ_H_ (ppm)** | **δ_C_ (ppm)** | **HMBC** | **^1^H-^1^H COSY** |
| --- | --- | --- | --- | --- |
| 1 | 1.18 [1H], m | 51.0 | 2.60 (H-3), 2.50 (H-3), 1.83 (H-10), 1.69 (H-2), 1.59 (H-2), 0.92 (H-14) | 1.69 (H-2), 1.59 (H-2) |
| 2 | 1.69 [1H], dddd, *J* = 16, 10, 6, 5 Hz | 21.4 | 2.60 (H-3), 2.50 (H-3) | 2.60 (H-3), 2.50 (H-3), 1.18 (H-1) |
|  | 1.59 [1H], m |  |  |  |
| 3 | 2.60 [1H], ddd, *J* = 17, 10, 5 Hz | 43.7 | 2.14 (H-15), 1.69 (H-2), 1.59 (H-2), 1.18 (H-1) | 1.69 (H-2), 1.59 (H-2) |
|  | 2.50 [1H], ddd, *J* = 17, 10, 6 Hz |  |  |  |
| 4 | - | 208.3 | 2.60 (H-3), 2.50 (H-3), 2.14 (H-15), 1.69 (H-2), 1.59 (H-2) | - |
| 5 | 9.98 [1H], dd, *J* = 2, 2 Hz | 204.8 | 1.98 (H-7), 1.18 (H-1) | - |
| 6 | _ | 89.8 | 1.98 (H-7), 1.78 (H-8), 1.69 (H-2), 1.59 (H-2), 1.18 (H-1) | - |
| 7 | 1.98 [1H], ddd, *J* = 14, 2, 2 Hz | 53.3 | 4.25 (H-13), 4.22 (H-13), 1.63 (H-8) | - |
| 8 | 1.78 [1H], br d, *J* = 14 Hz | 19.2 | 1.11 (H-9) | 1.87 (H-9), 1.11 (H-9) |
|  | 1.63 [1H], dd, *J* = 13, 4 Hz |  |  |  |
| 9 | 1.87 [1H], m | 34.8 | 1.63 (H-8), 0.92 (H-14) | 1.78 (H-8), 1.63 (H-8) |
|  | 1.11 [1H], dddd, *J* = 12, 12, 12, 4 Hz |  |  |  |
| 10 | 1.83 [1H], m | 33.3 | 1.69 (H-2), 0.92 (H-14) | 0.92 (H-14) |
| 11 | - | 70.0 | 4.25 (H-13), 4.22 (H-13), 1.98 (H-7), 1.63 (H-8) | - |
| 12 | - | 173.6 | 4.25 (H-13), 4.22 (H-13), 3.72 (CO_2_Me), 1.98 (H-7) | - |
| 13 | 4.25 [1H], d, *J* = 13 Hz | 76.4 | - | - |
|  | 4.22 [1H], d, *J* = 13 Hz |  |  |  |
| 14 | 0.92 [3H], d, *J* = 7 Hz | 20.2 | - | 1.83 (H-10) |
| 15 | 2.14 [3H], s | 30.0 | - | - |
| CO_2_Me | 3.72 [3H], s | 52.6 | - | - |

*Arteannuin V (****72****)* was purified from fraction J by HPLC (25% EtOAc/hexane/0.5% AcOH) yielding HPLC fraction 22 (*R_t_* 27.2 mins). Colourless oil. HRAPCIMS (*m/z*): 313.1665, Δ = -6.0 ppm for [M + H]^+^ (calc. 313.1646 for C_16_H_25_O_6_).

The stereochemistry of the 11-OH group in arteannuin V (**72**) could not be defined by NOESY correlations. However, on the assumption that the biogenesis of (**72**) involves opening of an 11,13-epoxide by the 6-OH group; and that this epoxide is formed by the same autoxidative processes (and therefore shares the same stereochemistry) as for Arteannuin X, the 11-OH may tentatively be assigned as β-, as drawn above.

Arteannuin W (**73**) (novel)

NMR data for arteannuin W (**73**) in CDCl_3_ solution

| **Position** | **δ_H_ (ppm)** | **δ_C_ (ppm)** | **HMBC** | **^1^H-^1^H COSY** |
| --- | --- | --- | --- | --- |
| 1 | 2.32 [1H], br d, *J* = 10 Hz | 37.4 | 2.46 (H-3), 1.61 (H-2), 1.51 (H-2), 0.84 (H-14) | - |
| 2 | 1.61 [1H], m | 28.8 | 2.46 (H-3) | 2.46 (H-3) |
|  | 1.51 [1H], m |  |  |  |
| 3 | 2.46 [2H], t, *J* = 7 Hz | 42.5 | 2.14 (H-15), 1.61 (H-2), 1.51 (H-2) | 1.61 (H-2), 1.51 (H-2) |
| 4 | - | 209.6 | 2.46 (H-3), 2.14 (H-15), 1.61 (H-2), 1.51 (H-2) | - |
| 5 | 10.23 [1H], s | 190.6 |  | - |
| 6 | - | 165.2 | 3.71 (H-11), 2.19 (H-8) | - |
| 7 | - | 134.5 | 3.71 (H-11), 2.19 (H-8), 1.12 (H-12), 1.11 (H-13) | - |
| 8 | 2.19 [2H], m | 21.1 | - | - |
| 9 | 1.76 [1H], m | 23.5 | 2.19 (H-8), 0.84 (H-14) | - |
|  | 1.38 [1H], m |  |  |  |
| 10 | 1.79 [1H], m | 28.4 | 2.19 (H-8), 0.84 (H-14) | 0.84 (H-14) |
| 11 | 3.71 [1H], qq, *J* = 7 , 7 Hz | 28.0 | 1.12 (H-12), 1.11 (H-13) | 1.12 (H-12), 1.11 (H-13) |
| 12 | 1.12 [3H], d, *J* = 7 Hz | 21.7 | 3.71 (H-11), 1.11 (H-13) | 3.71 (H-11) |
| 13 | 1.11 [3H], d, *J* = 7 Hz | 21.3 | 3.71 (H-11), 1.12 (H-12) | 3.71 (H-11) |
| 14 | 0.84 [3H], d, *J* = 7 Hz | 18.7 | - | 1.79 (H-10) |
| 15 | 2.14 [3H], s | 29.9 | - | — |

*Arteannuin W (****73****)* was purified from fraction H by HPLC (15% EtOAc/hexane/0.5% AcOH) yielding HPLC fraction 22 (*R_t_* 20.1 mins). Colourless oil. ESI-MS (*m/z*): 275.1618, ∆ = 0.5 ppm for [M + H^+^] (calc. for C_15_H_24_O_3_); R_t_ 6.37 mins.

*Nor-seco-cadinane / amorphane sesquiterpenes*

Arteannuin Y (**74**) (novel)

NMR data for arteannuin Y (**74**) in CDCl_3_ solution

| **Position** | **δ_H_ (ppm)** | **δ_C_ (ppm)** | **HMBC** | **^1^H-^1^H COSY** |
| --- | --- | --- | --- | --- |
| 1 | 2.13 [3H], s | 56.4 | 2.60 (H-3), 1.74 (H-2), 1.11 (H-14) | - |
| 2 | 1.87 [1H], dddd, *J* = 15, 8, 5, 3 Hz | 19.8 | 2.60 (H-3) | 2.60 (H-3), 2.41 (H-3) |
|  | 1.74 [1H], ddd, *J* = 15, 8, 3 Hz |  |  |  |
| 3 | 2.60 [1H], ddd, *J* = 17, 8, 5 Hz | 41.1 | 2.13 (H-15), 1.74 (H-2) | 1.87 (H-2), 1.74 (H-2) |
|  | 2.41 [1H], ddd, *J* = 17, 8, 3 Hz |  |  |  |
| 4 | - | 209.1 | 2.60 (H-3), 2.41 (H-3), 2.13 (H-15) | - |
| 6 | - | 210.2 | 2.94 (H-7) | - |
| 7 | 2.94 [1H], dd, *J* = 13, 5 Hz | 52.1 | 2.80 (H-13) | 2.02 (H-8), 1.61 (H-8) |
| 8 | 2.02 [1H], dddd, *J* = 12, 6, 3, 3 Hz | 28.0 | 2.94 (H-7) | 2.94 (H-7) |
|  | 1.61 [1H], m |  |  |  |
| 9 | 1.93 [1H], dddd, *J* = 13, 3, 3, 3 Hz | 34.0 | 1.11 (H-14) | - |
|  | 1.52 [1H], dddd, *J* = 13, 12, 12, 3 Hz |  |  |  |
| 10 | 1.61 [1H], m | 39.8 | 1.11 (H-14) | 1.11 (H-14) |
| 11 | - | 55.3 | 3.22 (H-13), 2.94 (H-7), 2.80 (H-13) | - |
| 12 | - | 170.4 | 3.75 (CO_2_Me), 3.22 (H-13), 2.94 (H-7), 2.80 (H-13) | - |
| 13 | 3.22 [1H], d, *J* = 5 Hz | 49.1 | 2.94 (H-7) | - |
|  | 2.80 [1H], d, *J* = 5 Hz |  |  |  |
| 14 | 1.11 [3H], d, *J* = 6 Hz | 20.5 | - | 1.61 (H-10) |
| 15 | 2.13 [3H], s | 30.0 | - | - |
| CO_2_Me | 3.75 [3H], s | 52.7 | - | - |

*Arteannuin Y (****74****)* was purified from fraction K by HPLC (40% EtOAc/hexane/1% AcOH) yielding HPLC fraction 18 (*R_t_* 16.0 mins). Colourless oil. HRAPCIMS (*m/z*): 283.1544, Δ = -1.3 ppm for [M + H]^+^ (calc. 283.1540 for C_15_H_23_O_5_).

The stereochemistry at the 11-position in arteannuin Y (**74**) could not be defined by NOESY correlations. However, on the assumption that the biogenesis of (**74**) involves formation of an 11,13-epoxide by autoxidative processes similar to those responsible for forming the epoxide group in arteannuin X, the O-substituent might tentatively be assigned as 11β-, as drawn above.

Norannuic acid (**75**) (known^1^)

NMR data for norannuic acid (**75**) in CDCl_3_ solution

| **Position** | **δ_H_ (ppm)** | **δ_C_ (ppm)** | **HMBC** | **^1^H-^1^H COSY** |
| --- | --- | --- | --- | --- |
| 1 | 1.51 [1H], m | 45.4 | 0.88 (H-14) | 2.31 (H-6) |
| 2 | 1.96 [1H], br d, *J* = 14 Hz | 29.1 | - | 2.24 (H-3), 2.16 (H-3) |
|  | 1.41 [1H], m |  |  |  |
| 3 | 2.24 [1H], br d, *J* = 14 Hz | 29.8 | 4.94 (H-13), 4.80 (H-13) | 1.96 (H-2), 1.41 (H-2) |
|  | 2.16 [1H], dd, *J* = 14 , 14 Hz |  |  |  |
| 4 | - | 151.3 | 4.94 (H-13), 4.80 (H-13), 4.43 (H-5) | - |
| 5 | 4.43 [1H], d, *J* = 12 Hz | 69.9 | 4.94 (H-13), 4.80 (H-13) | 4.94 (H-13), 4.80 (H-13),  2.31 (H-6) |
| 6 | 2.31 [1H], ddd, *J* = 12, 3, 3 Hz | 49.1 | 4.43 (H-5) | 2.44 (H-7), 1.51 (H-1), 4.43 (H-5) |
| 7 | 2.44 [1H], ddd, *J* = 12, 3, 3 Hz | 45.7 | - | 2.31 (H-6), 1.86 (H-8) |
| 8 | 1.86 [1H], m | 22.3 | - | 2.44 (H-7) |
|  | 1.83 [1H], m |  |  |  |
| 9 | 1.89 [1H], m | 35.1 | 0.88 (H-14) | - |
|  | 1.05 [1H], m |  |  |  |
| 10 | 1.74 [1H], m | 28.3 | 0.88 (H-14) | 0.88 (H-14) |
| 11 | - | 179.7 | 2.44 (H-7) | - |
| 14 | 0.88 [3H], d, *J* = 6 Hz | 20.0 | - | 1.74 (H-10) |
| 15 | 4.94 [1H], m | 105.4 | - | 4.43 (H-5) |
|  | 4.80 [1H], m |  |  |  |

*Norannuic acid (****75****)* was purified from fraction K by HPLC (40% EtOAc/hexane/1% AcOH) yielding HPLC fraction 14 (*R_t_* 13.0 mins). Colourless oil. HRAPCIMS (*m/z*): 207.1378, Δ = 0.9 ppm for [M -H_2_O + H]^+^ (calc. 207.1380 for C_13_H_19_O_2_; M^+^ C_13_H_20_O_3_).

1. L. N. Misra; A. Ahmad; R. S. Thakur; J. Jakupovic. “Bisnor-cadinanes from *Artemisia annua* and definitve carbon-13 NMR assignments of β-arteether”. *Phytochemistry* **33**(6), 1461-1464 (1993).

Norannuic acid formyl ester (**76**) (known^1^)

NMR data for norannuic acid formyl ester (**76**) in CDCl_3_ solution

| **Position** | **δ_H_ (ppm)** | **δ_C_ (ppm)** | **HMBC** | **^1^H-^1^H COSY** |
| --- | --- | --- | --- | --- |
| 1 | 1.60 [1H], br d, *J* = 13 Hz | 45.2 | 0.91 (H-14) | 2.54 (H-6) |
| 2 | 2.01 [1H], br d, *J* = 14 Hz | 28.8 |  | 2.27 (H-3), 2.24 (H-3) |
|  | 1.46 [1H], dddd, *J* = 14, 14, 4, 4 Hz |  |  |  |
| 3 | 2.27 [1H], br d, *J* = 14 Hz | 29.6 | 4.78 (H-15), 4.57 (H-15) | 2.01 (H-2), 1.46 (H-2) |
|  | 2.24 [1H], br dd, *J* = 14, 14 Hz |  |  |  |
| 4 | - | 145.6 | 5.76 (H-5), 4.78 (H-15), 4.57 (H-15), 2.54 (H-6), 2.27 (H-3), 2.01 (H-2) | — |
| 5 | 5.76 [1H], d, *J* = 12 Hz | 70.6 | 8.07 (H-13), 4.78 (H-14), 2.54 (H-6), 2.48 (H-7), 2.27 (H-3), 2.24 (H-3), 1.60 (H-1) | 2.54 (H-6) |
| 6 | 2.54 [1H], ddd, *J* = 12, 4, 4 Hz | 45.3 | 5.76 (H-5) | 5.76 (H-5), 2.48 (H-7), 1.60 (H-1) |
| 7 | 2.48 [1H], ddd, *J* = 13, 3, 3 Hz | 45.7 |  | 2.54 (H-6), 1.75 (H-8), 1.71 (H-8) |
| 8 | 1.75 [1H], m | 21.8 |  | 2.48 (H-7) |
|  | 1.71 [1H], m |  |  |  |
| 9 | 1.90 [1H], m | 34.5 | 0.91 (H-14) | - |
|  | 1.05 [1H], dddd, *J* = 13, 13, 13, 4 Hz |  |  |  |
| 10 | 1.85 [1H], m | 27.9 | 0.91 (H-14) | 0.91 (H-14) |
| 11 | - | 181.2 | 2.54 (H-6), 2.48 (H-7), 1.75 (H-8), 1.71 (H-8) | - |
| 13 | 8.07 [1H], s | 160.4 | 5.76 (H-5) | - |
| 14 | 0.91 [3H], d, *J* = 7 Hz | 19.9 |  | 1.85 (H-10) |
| 15 | 4.78 [1H], br s | 105.7 | 5.76 (H-5) | - |
|  | 4.57 [1H], br s |  |  |  |

*Norannuic acid formyl ester (****76****)* was purified from fraction K by HPLC (40% EtOAc/hexane/1% AcOH) yielding HPLC fraction 7 (*R_t_* 9.5 mins). Colourless oil. HRAPCIMS (*m/z*): 207.1380, Δ = -0.2 ppm for [M -HCOOH + H]^+^ (calc. 207.1380 for C_13_H_19_O_2_; M^+^ C_14_H_20_O_4_).

1. G. D. Brown; G.-Y. Liang; L.-K. Sy “Terpenoids from the seeds of *Artemisia annua*”. *Phytochemistry* **64** 303-323 (2003).

Isoarteannuin A (**77**) (novel^1,2^)

NMR data for isoarteannuin A (**77**) in CDCl_3_ solution

| **Position** | **δ_H_ (ppm)** | **δ_C_ (ppm)** | **HMBC** | **^1^H-^1^H COSY** |
| --- | --- | --- | --- | --- |
| 1 | 1.61 [1H], m | 39.7 | 0.96 (H-14) | - |
| 2 | 1.98 [1H], m | 27.9 | - | 2.29 (H-3), 2.14 (H-3) |
|  | 1.58 [1H], m |  |  |  |
| 3 | 2.29 [1H], br d, *J* = 13 Hz | 29.5 | 4.93 (H-15), 4.82 (H-5), 4.79 (H-15) | 1.98 (H-2), 1.58 (H-2) |
|  | 2.14 [1H], ddd, *J* = 13, 13, 5 Hz |  |  |  |
| 4 | - | 144.7 | 4.93 (H-15), 4.82 (H-5), 2.29 (H-3), 2.21  (H-6), 2.14 (H-3), 1.98 (H-2) | - |
| 5 | 4.82 [1H], d, *J* = 12 Hz | 78.8 | 4.93 (H-15), 4.79 (H-15), 2.60  (H-7), 2.29 (H-3), 2.21 (H-6) | 2.21 (H-6) |
| 6 | 2.21 [1H], ddd, *J* = 12, 4, 4 Hz | 48.7 | 4.82 (H-5) | 4.82 (H-5), 2.60 (H-7) |
| 7 | 2.60 [1H], ddd, *J* = 12, 4, 4 Hz | 41.0 | 4.82 (H-5) | 2.21 (H-6), 1.98 (H-8), 1.64 (H-8) |
| 8 | 1.98 [1H], m | 21.8 | - | 2.60 (H-7) |
|  | 1.64 [1H], m |  |  |  |
| 9 | 1.81 [1H], dddd, *J* = 13, 3, 3, 3 Hz | 33.4 | 0.96 (H-14) | - |
|  | 1.03 [1H], m |  |  |  |
| 10 | 1.66 [1H], m | 29.2 | 0.96 (H-14) | 0.96 (H-14) |
| 11 | - | 178.2 | 2.60 (H-7), 1.64 (H-8) | - |
| 14 | 0.96 [3H], d, *J* = 6 Hz | 19.9 | - | 1.66 (H-10) |
| 15 | 4.93 [1H], s | 103.0 | 4.82 (H-5), 2.29 (H-3), 2.14 (H-3) | - |
|  | 4.79 [1H], s |  |  |  |

*Isoarteannuin A (****77****)* was purified from fraction K by HPLC (40% EtOAc/hexane/1% AcOH) yielding HPLC fraction 5 (*R_t_* 8.5 mins). Colourless oil. ESI-MS (*m/z*): 207.1380, ∆ = 0.2 ppm for [M + H^+^] (calc. for C_13_H_19_O_2_); R_t_ 6.79 mins.

Arteannuin A from *A. annua*, is the Δ^3,4^-double bond isomer of novel natural product (**77**):

1.Y. Tian; Z.-X. Wei; Z.-H. Wu. “Studies on the chemical constituents of Qinghao (*Artemisia annua*), a traditional Chinese herb”. *Zhongcaoyao* **13**(6), 9-11(1982).

2.W.-S. Zhou; L. Zhang; X.-X. Xu. “Structure and synthesis of arteannuin and related compounds. XVIII. Synthesis of arteannuin A”. *Huaxue Xuebao* **44**(9), 968-970 (1986).

Arteannuin Z (**78**) (novel)

NMR data for arteannuin Z (**78**) in CDCl_3_ solution

| **Position** | **δ_H_ (ppm)** | **δ_C_ (ppm)** | **HMBC** | **^1^H-^1^H COSY** |
| --- | --- | --- | --- | --- |
| 1 | 1.52 [1H], m | 41.5 | 1.99 (H-2), 0.98 (H-14) | 1.99 (H-2), 1.30 (H-2) |
| 2 | 1.99 [1H], ddd, *J* = 13, 5, 5 Hz | 22.5 | 1.80 (H-3) | 1.69 (H-3), 1.52 (H-1) |
|  | 1.30 [1H], m |  |  |  |
| 3 | 1.80 [1H], dd, *J* = 12, 6 Hz | 34.4 | 1.99 (H-2), 1.56 (H-15) | 1.99 (H-2), 1.30 (H-2) |
|  | 1.69 [1H], ddd, *J* = 12, 12, 6 Hz |  |  |  |
| 4 | - | 113.2 | 5.90 (H-5), 1.99 (H-2), 1.80 (H-3), 1.69 (H-3), 1.56 (H-15) | - |
| 5 | 5.90 [1H], s | 101.7 | 2.67 (H-7) | - |
| 6 | - | 89.3 | 5.90 (H-5), 2.67 (H-7), 1.99 (H-2) | - |
| 7 | 2.67 [1H], dd, *J* = 14, 8 Hz | 45.4 | - | 2.25 (H-8), 1.36 (H-8) |
| 8 | 2.25 [1H], m | 26.7 | - | 2.67 (H-7), 1.75 (H-9) |
|  | 1.36 [1H], m |  |  |  |
| 9 | 1.75 [1H], dddd, *J* = 13, 3, 3, 3 Hz | 32.1 | 0.98 (H-14) | - |
|  | 1.13 [1H], dddd, *J* = 13, 13, 13, 3 Hz |  |  |  |
| 10 | 1.24 [1H], m | 35.1 | 0.98 (H-14) | 0.98 (H-14) |
| 11 | - | 175.8 | 5.90 (H-5), 2.67 (H-7) | - |
| 14 | 0.98 [3H], d, *J* = 6 Hz | 18.4 | - | 1.24 (H-10) |
| 15 | 1.56 [3H], s | 23.8 | - | - |

*Arteannuin Z (****78****)* was purified from fraction J by HPLC (25% EtOAc/hexane/0.5% AcOH) yielding HPLC fraction 10 (*R_t_* 12.6 mins). Colourless oil. ESI-MS (*m/z*): 239.1278, ∆ = 0.3 ppm for [M + H^+^] (calc. for C_13_H_19_O_4_); R_t_ 5.86 mins. HRAPCIMS (*m/z*): 239.1282, Δ = -1.9 ppm for [M + H]^+^ (calc. 239.1278 for C_13_H_19_O_4_).

*Miscellaneous sesquiterpenes*

3-(2-(2,5-Dihydrofuran-3-yl)ethyl)-2,2-dimethyl-4-methylenecyclohexan-1-one (**79**) (novel)

NMR data for 3-(2-(2,5-dihydrofuran-3-yl)ethyl)-2,2-dimethyl-4-methylenecyclohexan-1-one (**79**) in CDCl_3_ solution

| **Position** | **δ_H_ (ppm)** | **δ_C_ (ppm)** | **HMBC** | **^1^H-^1^H COSY** |
| --- | --- | --- | --- | --- |
| 1 | 2.19 [1H], dd, *J* = 12, 3 Hz | 55.9 | 5.05 (H-15), 4.86 (H-15), 1.21 (H-13), 1.06 (H-14) | 1.23 (H-7) |
| 2 | - | 144.5 | 2.48 (H-3), 2.19 (H-1) | - |
| 3 | 2.48 [2H], m | 30.5 | 5.05 (H-15), 4.86 (H-15), 2.19 (H-1) | 2.65 (H-4), 2.32 (H-4) |
| 4 | 2.65 [1H], ddd, *J* = 14, 12, 7 Hz | 37.6 | - | 2.48 (H-3) |
|  | 2.32 [1H], ddd, *J* = 14, 6, 2 Hz |  |  |  |
| 5 | - | 215.1 | 2.65 (H-4), 2.48 (H-3), 2.32 (H-4),  2.19 (H-1), 1.21 (H-13), 1.06 (H-14) | - |
| 6 | - | 49.1 | 2.19 (H-1), 1.21 (H-13), 1.06 (H-14) | - |
| 7 | 1.72 [1H], m | 24.8 | 2.19 (H-1) | 2.19 (H-1) |
|  | 1.23 [1H], m |  |  |  |
| 8 | 1.94 [1H], m | 30.0 | - | - |
|  | 1.79 [1H], m |  |  |  |
| 9 | - | 135.1 | 4.42 (H-12) | - |
| 10 | 5.62 [1H], br s | 117.3 | - | 4.56 (H-11), 4.42 (H-12) |
| 11 | 4.56 [2H], br | 70.0 | - | 5.62 (H-10) |
| 12 | 4.42 [2H], br | 72.5 | - | 5.62 (H-10) |
| 13 | 1.21 [3H], s | 27.2 | 1.06 (H-14) | - |
| 14 | 1.06 [3H], s | 21.3 | 1.21 (H-13) | - |
| 15 | 5.05 [1H], s | 113.7 | 2.48 (H-3), 2.19 (H-1) | - |
|  | 4.86 [1H], s |  |  |  |

*3-(2-(2,5-Dihydrofuran-3-yl)ethyl)-2,2-dimethyl-4-methylenecyclohexan-1-one (****79****)* was purified from fraction F by HPLC (8% EtOAc/hexane/0.5% AcOH) yielding HPLC fraction 19 (*R_t_* 19.9 mins). Colourless oil.

*iv) Triterpenes*

α-Amyrin (**80**) (known)

NMR data for α-amyrin (**80**) in CDCl_3_ solution

| **Position** | **δ_H_ (ppm)** | **δ_C_ (ppm)** | **HMBC** | **^1^H-^1^H COSY** |
| --- | --- | --- | --- | --- |
| 1 | 1.66 [1H], m | 38.7 | 0.96 (H-25) | - |
|  | 1.03 [1H], m |  |  |  |
| 2 | 1.62 [1H], m | 27.3 | - | - |
|  | 1.59 [1H], m |  |  |  |
| 3 | 3.23 [1H], dd, *J* = 12, 4 Hz | 79.1 | 1.00 (H-24), 0.79 (H-23) | - |
| 4 | - | 38.8 | 1.00 (H-24), 0.79 (H-23) | - |
| 5 | 0.74 [1H], m | 55.2 | 1.00 (H-24), 0.96 (H-25), 0.79 (H-23) | - |
| 6 | 1.55 [1H], m | 18.2 | - | - |
|  | 1.41 [1H], m |  |  |  |
| 7 | 1.55 [1H], m | 32.9 | 1.01 (H-26) | - |
|  | 1.36 [1H], m |  |  |  |
| 8 | - | 40.0 | 1.07 (H-27), 1.01 (H-26) | - |
| 9 | 1.52 [1H], m | 47.7 | 1.01 (H-26), 0.96 (H-25) | - |
| 10 | - | 15.7 | 0.96 (H-25) | - |
| 11 | 1.95 [2H], m | 23.4 | - | - |
| 12 | 5.12 [1H], br | 124.4 | - | - |
| 13 | - | 139.6 | 1.07 (H-27) | - |
| 14 |  | 42.1 | 1.07 (H-27), 1.01 (H-26) | - |
| 15 | 1.83 [1H], m | 26.6 | 1.07 (H-27) | - |
|  | 0.98 [1H], m |  |  |  |
| 16 | 2.00 [1H], m | 28.1 | 0.79 (H-28) | - |
|  | 0.87 [1H], m |  |  |  |
| 17 | - | 33.8 | 0.79 (H-28) | - |
| 18 | 1.31 [1H], m | 59.0 | 0.80 (H-29), 0.79 (H-28) | - |
| 19 | 1.31 [1H], m | 39.7 | 0.92 (H-30), 0.80 (H-29) | - |
| 20 | 0.87 [1H], m | 39.6 | 0.92 (H-30), 0.80 (H-29) | - |
| 21 | 1.38 [1H], m | 31.2 | 0.92 (H-30) | - |
|  | 1.26 [1H], m |  |  |  |
| 22 | 1.43 [1H], m | 41.5 | 0.79 (H-28) | - |
|  | 1.28 [1H], m |  |  |  |
| 23 | 0.79 [3H], s | 15.6 | 1.00 (H-24) | - |
| 24 | 1.00 [3H], s | 28.2 | 0.79 (H-23) | - |
| 25 | 0.96 [3H], s | 15.7 | - | - |
| 26 | 1.01 [3H], s | 16.9 | - | - |
| 27 | 1.07 [3H], s | 23.3 | - | - |
| 28 | 0.79 [3H], s | 17.5 | - | - |
| 29 | 0.80 [3H], d, *J* = 7 Hz | 28.8 | - | 1.31 (H-19) |
| 30 | 0.92 [3H], d, *J* = 7 Hz | 21.4 | - | 0.87 (H-20) |

*a-Amyrin (****80****)* was purified from fraction F by HPLC (8% EtOAc/hexane/0.5% AcOH) yielding HPLC fraction 20 (*R_t_* 22.1 mins). Colourless oil.

β-Amyrin (**81**) (known)

NMR data for β-Amyrin (**81**) in CDCl_3_ solution

| **Position** | **δ_H_ (ppm)** | **δ_C_ (ppm)** | **HMBC** | **^1^H-^1^H COSY** |
| --- | --- | --- | --- | --- |
| 1 | 1.63 [1H], m | 38.6 | 0.94 (H-25) | - |
|  | 0.97 [1H], m |  |  |  |
| 2 | 1.62 [1H], m | 27.2 | - | 3.23 (H-3) |
|  | 1.59 [1H], m |  |  |  |
| 3 | 3.23 [1H], dd, *J* = 12, 4 Hz | 79.0 | 1.62 (H-2), 1.00 (H-23), 0.79 (H-24) | 1.62 (H-2), 1.59 (H-2) |
| 4 | - | 38.8 | 1.00 (H-23), 0.79 (H-24) | - |
| 5 | 0.74 [1H], d, *J* = 12 Hz | 55.1 | 1.00 (H-23), 0.94 (H-25), 0.79 (H-24) | 1.41 (H-6) |
| 6 | 1.55 [1H], m | 18.4 | - | 0.74 (H-5) |
|  | 1.41 [1H], m |  |  |  |
| 7 | 1.53 [1H], m | 32.6 | 0.97 (H-26) | - |
|  | 1.33 [1H], m |  |  |  |
| 8 | - | 39.8 | 1.77 (H-15), 1.55 (H8), 1.13 (H-27), 0.97 (H-26) | - |
| 9 | 1.55 [1H], m | 47.6 | 5.18 (H-12), 0.97 (H-26), 0.94 (H-25) | 1.85 (H-11) |
| 10 | - | 36.9 | 1.55 (H-19), 0.94 (H-25) | - |
| 11 | 1.90 [1H], ddd, *J* = 18, 12, 3 Hz | 23.5 | 1.55 (H-19) | 5.18 (H-12) |
|  | 1.85 [1H], m |  |  |  |
| 12 | 5.18 [1H], t, *J* = 4 Hz | 121.7 | 1.95 (H-18), 1.85 (H-11) | 1.90 (H-11), 1.85 (H-11) |
| 13 | - | 145.2 | 1.95 (H-18), 1.90 (H-11), 1.85 (H-11), 1.66 (H-19), 1.13 (H-27) |  |
| 14 | - | 41.7 | 5.18 (H-12), 1.95 (H18), 1.77 (H-15), 1.55 (H-6), 1.13 (H-27), 0.97 (H-26) | - |
| 15 | 1.77 [1H], ddd, *J* = 14, 14, 6 Hz | 26.1 | 1.13 (H-27) | - |
|  | 0.79 [1H], m |  |  |  |
| 16 | 2.00 [1H], ddd, *J* = 14, 12, 5 Hz | 26.9 | 0.83 (H-28) | - |
|  | 0.81 [1H], m |  |  |  |
| 17 | - | 32.5 | 1.43 (H-22), 0.83 (H-28) | - |
| 18 | 1.95 [1H], dd, *J* = 14, 5 Hz | 47.2 | 1.66 (H-19), 0.83 (H-28) | - |
| 19 | 1.66 [1H], m | 46.8 | 0.87 (H-29), 0.87 (H-30) | - |
|  | 1.01 [1H], m |  |  |  |
| 20 | - | 31.1 | 1.66 (H-19), 0.87 (H-29), 0.87 (H-30) | - |
| 21 | 1.34 [1H], m | 34.7 | 0.87 (H-29), 0.87 (H-30) | - |
|  | 1.10 [1H], m |  |  |  |
| 22 | 1.43 [1H], m | 37.1 | 1.34 (H-21), 0.83 (H-28) | - |
|  | 1.22 [1H], ddd, *J* = 13, 3, 3 Hz |  |  |  |
| 23 | 1.00 [3H], s | 28.1 | 3.23 (H-3), 0.79 (H-24) | - |
| 24 | 0.79 [3H], s | 15.6 | 3.23 (H-3), 1.00 (H-23) | - |
| 25 | 0.94 [3H], s | 15.5 | 1.55 (H-9) | - |
| 26 | 0.97 [3H], s | 16.8 | 1.55 (H-9) | - |
| 27 | 1.13 [3H], s | 26.0 | - | - |
| 28 | 0.83 [3H], s | 28.4 | - | - |
| 29 | 0.87 [3H], s | 33.3 | 1.66 (H-19), 0.87 (H-30) | - |
| 30 | 0.87 [3H], s | 23.7 | 1.66 (H-19), 0.87 (H-29) | - |

*β-Amyrin (****81****)* was purified from fraction F by HPLC (8% EtOAc/hexane/0.5% AcOH) yielding HPLC fraction 21 (*R_t_* 23.0 mins). Colourless oil.

25,26-Dinorolean-5-en-3-ol, 9,13-dimethyl (**82**) (known)

NMR data for 25,26-dinorolean-5-en-3-ol, 9,13-dimethyl (**82**) in CDCl_3_ solution

| **Position** | **δ_H_ (ppm)** | **δ_C_ (ppm)** | **HMBC** | **^1^H-^1^H COSY** |
| --- | --- | --- | --- | --- |
| 1 | 1.54 [1H], m | 18.2 | - | - |
|  | 1.47 [1H], m |  |  |  |
| 2 | 1.86 [1H], m | 27.8 | - | 3.47 (H-3) |
|  | 1.70 [1H], m |  |  |  |
| 3 | 3.47 [1H], dd, *J* = 3, 3 Hz | 76.4 | 1.14 (H-24), 1.04 (H-23) | 1.86 (H-2), 1.70 (H-2) |
| 4 | - | 40.8 | 1.14 (H-24), 1.04 (H-23) | - |
| 5 | - | 141.6 | 1.14 (H-24), 1.04 (H-23) | - |
| 6 | 5.63 [1H], d, *J* = 7 Hz | 122.1 | - | 1.98 (H-7), 1.84 (H-7) |
| 7 | 1.98 [1H], m | 23.6 | - | 5.63 (H-6) |
|  | 1.84 [1H], m |  |  |  |
| 8 | 1.51 [1H], m | 47.4 | 1.09 (H-26), 0.85 (H-25) | - |
| 9 | - | 34.8 | 0.85 (H-25) | - |
| 10 | 2.01 [1H], m | 49.6 | 0.85 (H-25) | - |
| 11 | 1.54 [1H], m | 34.6 | 0.85 (H-25) | - |
|  | 1.40 [1H], m |  |  |  |
| 12 | 1.35 [1H], m | 30.3 | 1.00 (H-27) | - |
|  | 1.35 [1H], m | - | - | - |
| 13 | - | 39.3 | 1.09 (H-26), 1.00 (H-27) | - |
| 14 | - | 37.8 | 1.09 (H-26), 1.00 (H-27) | - |
| 15 | 1.47 [1H], m | 32.0 | 1.09 (H-26) | - |
|  | 1.30 [1H], m |  |  |  |
| 16+ | 1.54 [1H], m | 36.0 | 1.16 (H-28) | - |
|  | 1.38 [1H], m |  |  |  |
| 17 | - | 30.1 | 1.16 (H-28) | - |
| 18 | 1.57 [1H], m | 43.0 | 1.16 (H-28), 1.00 (H-27) | - |
| 19* | 1.47 [1H], m | 33.1 | 0.99 (H-29), 0.95 (H-30) | - |
|  | 1.25 [1H], m |  |  |  |
| 20 | - | 28.2 | 0.99 (H-29), 0.95 (H-30) | - |
| 21* | 1.38 [1H], m | 35.0 | 0.99 (H-29), 0.95 (H-30) | - |
|  | 1.25 [1H], m |  |  |  |
| 22+ | 1.54 [1H], m | 38.9 | 1.16 (H-28) | - |
|  | 0.92 [1H], m |  |  |  |
| 23 | 1.04 [3H], s | 28.9 | 1.14 (H-24) | - |
| 24 | 1.14 [3H], s | 25.5 | 1.04 (H-23) | - |
| 25 | 0.85 [3H], s | 16.2 | - | - |
| 26 | 1.09 [3H], s | 19.6 | - | - |
| 27 | 1.00 [3H], s | 18.4 | - | - |
| 28 | 1.16 [3H], s | 32.0 | - | - |
| 29 | 0.99 [3H], s | 32.4 | 0.95 (H-30) | - |
| 30 | 0.95 [3H], s | 34.5 | - 1. H-29) | - |

*Interchangeable assignments

+ Interchangeable assignments

*25,26-Dinorolean-5-en-3-ol, 9,13-dimethyl (****82****)* was purified from fraction E by HPLC (7% EtOAc/hexane) yielding HPLC fraction 22 (*R_t_* 21.9 mins). Colourless oil.

*v) Aliphatic hydrocarbons, alcohols, aldehydes and acids*

Trilinolein (**83**) (known)

NMR data for trilinolein (**83**) in CDCl_3_ solution

| **Position** | **δ_H_ (ppm)** | **δ_C_ (ppm)** | **HMBC** | **^1^H-^1^H COSY** |
| --- | --- | --- | --- | --- |
| 1 | - | 173.3* | 4.29 (H-1’), 4.15 (H-1’), 2.31 (H-2), 1.61 (H-3) | - |
| 2^+^ | 2.31 [2H], t, *J* = 7 Hz | 34.0 | - | 1.61 (H-3) |
| 3 | 1.61 [2H], quin, *J* = 7 Hz | 24.8^++^ | 2.31 (H-2) | 2.31 (H-2) |
| 4 | 1.30 [2H], m | 29.7 | 2.31 (H-2) | - |
| 5 | 1.25 [2H], m | 29.6 | - | - |
| 6 | 1.30 [2H], m | 29.1 | - | - |
| 7 | 1.30 [2H], m | 29.2 | - | - |
| 8 | 2.05 [2H], m | 29.3 | - | 5.36 (H-9) |
| 9 | 5.36 [1H], m | 127.9 | - | 2.05 (H-8) |
| 10 | 5.33 [1H], m | 128.1 | - | 2.77 (H-11) |
| 11 | 2.77 [2H], t, *J* = 7 Hz | 25.6 | 5.35 (H-12), 5.33 (H-10) | 5.35 (H-12), 5.33 (H-10) |
| 12 | 5.35 [1H], m | 130.7 | - | 2.77 (H-11) |
| 13 | 5.38 [1H], m | 130.0 | - | 2.05 (H-14) |
| 14 | 2.05 [2H], m | 27.2 | 5.38 (H-13), 5.35 (H-12) | 5.38 (H-13) |
| 15 | 1.30 [2H], m | 29.1 | - | - |
| 16 | 1.29 [2H], m | 22.7 | 0.88 (H-18) | - |
| 17 | 1.33 [2H],t, *J* = 7 Hz | 31.5 | 0.88 (H-18) | 0.88 (H-18) |
| 18 | 0.88 [3H], t, *J* = 7 Hz | 14.1 | - | 1.33 (H-17) |
| 1’’ | 4.29 [1H], dd, *J* = 12, 6 Hz | 62.1 | 5.27 (H-2’’), 4.29 (H-1’), 4.15 (H-1’) | 5.27 (H-2’’) |
|  | 4.15 [1H], dd,, *J* = 12, 6 Hz |  |  |  |
| 2’’ | 5.27 [1H], tt, *J* = 12, 6 Hz | 68.8 | 4.29 (H-1’), 4.15 (H-1’) | 4.29 (H-1’), 4.15 (H-1’) |

* δ_C_ 172.9 ppm for C-1’ is resolved from C-1.

^+^ δ_C_ 34.2 ppm for C-2’ is resolved from C-2; δ_H_ 2.32 [2H], t, *J* = 7 Hz for H-2’ is resolved from H-2.

^++^ δ_C_ 24.9 ppm for C-3’ is resolved from C-3.

N. B. Resonances at the 2’’-position as well as the central linolenic acid ester are roughly half the intensity of resonances at the 1’’-position and for the outer linoleic acid ester resonances because there are two of the latter, which are identical by symmetry.

*Trilinolein (****83****)* was purified from fraction E by HPLC (7% EtOAc/hexane) yielding HPLC fraction 10 (*R_t_* 12.5 mins). Colourless oil.

Trilinolenin (**84**) (known)

NMR data for trilinolenin (**84**) in CDCl_3_ solution

| **Position** | **δ_H_ (ppm)** | **δ_C_ (ppm)** | **HMBC** | **^1^H-^1^H COSY** |
| --- | --- | --- | --- | --- |
| 1 | - | 173.3* | 4.29 (H-1’), 4.15 (H-1’), 2.31 (H-2), 1.61 (H-3) | - |
| 2^+^ | 2.31 [2H], t, *J* = 7 Hz | 34.0 | - | 1.61 (H-3) |
| 3 | 1.61 [2H], quin, *J* = 7 Hz | 24.8^++^ | 2.31 (H-2) | 2.31 (H-2) |
| 4 | 1.30 [2H], m | 29.7 | 2.31 (H-2) | - |
| 5 | 1.25 [2H], m | 29.6 | - | - |
| 6 | 1.30 [2H], m | 29.1 | - | - |
| 7 | 1.30 [2H], m | 29.2 | - | - |
| 8 | 2.05 [2H], m | 27.2 | - | 5.33 (H-9) |
| 9 | 5.33 [1H], m | 127.1 | 2.81 (H-11) | 2.05 (H-8) |
| 10 | 5.33 [1H], m | 127.8 | 2.05 (H-8) | 2.81 (H-11) |
| 11 | 2.81 [2H], br t, *J* = 7 Hz | 25.6 | 5.35 (H-12), 5.33 (H-10) | 5.35 (H-12), 5.33 (H-10) |
| 12 | 5.35 [1H], m | 128.2 | - | 2.81 (H-11) |
| 13 | 5.36 [1H], m | 128.3 | - | 2.81 (H-14) |
| 14 | 2.81 [2H], br t, *J* = 7 Hz | 25.5 | 5.38 (H-15), 5.35 (H-12) | 5.38 (H-15), 5.36 (H-13) |
| 15 | 5.38 [1H], m | 130.2 | - | 2.81 (H-14) |
| 16 | 5.40 [1H], m | 132.0 | 0.98 (H-18) | 2.09 (H-17) |
| 17 | 2.09 [2H], dq, *J* = 7, 7 Hz | 20.5 | 0.98 (H-18) | 5.40 (H-16), 0.98 (H-18) |
| 18 | 0.98 [3H], t, *J* = 7 Hz | 14.1 | - | 2.09 (H-17) |
| 1’’ | 4.29 [1H], dd, *J* = 12, 6 Hz | 62.1 | 5.27 (H-2’’), 4.29 (H-1’), 4.15 (H-1’) | 5.27 (H-2’’) |
|  | 4.15 [1H], dd,, *J* = 12, 6 Hz |  |  |  |
| 2’’ | 5.27 [1H], tt, *J* = 12, 6 Hz | 68.8 | 4.29 (H-1’), 4.15 (H-1’) | 4.29 (H-1’), 4.15 (H-1’) |

* δ_C_ 172.9 ppm for C-1’ is resolved from C-1.

^+^ δ_C_ 34.2 ppm for C-2’ is resolved from C-2); δ_H_ 2.32 [2H], t, *J* = 7 Hz for H-2’ is resolved from H-2).

^++^ δ_C_ 24.9 ppm for C-3’ is resolved from C-3.

N. B. Resonances at the 2’’-position as well as the central linolenic acid ester are roughly half the intensity of resonances at the 1’’-position and for the outer linoleic acid ester resonances because there are two of the latter, which are identical by symmetry.

*Trilinolenin (****84****)* was purified from fraction E by HPLC (7% EtOAc/hexane) yielding HPLC fraction 11 (*R_t_* 13.5 mins). Colourless oil.

Pontica epoxide (**32**) (known) – see Supplementary List 1 (Artemis) for NMR data

*Pontica epoxide* (**32**) was purified from fraction B by HPLC (2% EtOAc/hexane) yielding HPLC fraction 15 (*R_t_* 16.2 mins). Colourless oil.

*vi) Aromatic alcohols, ketones and acids*

2,4-Dihydroxy-6-methoxy-acetophenone (**34**) (known^1^) – see Supplementary List 1 (Artemis) for NMR data

*2,4-Dihydroxy-6-methoxy acetophenone* (**34**) was purified from fraction J by HPLC (25% EtOAc/hexane/0.5% AcOH) yielding HPLC fraction 15 (*R_t_* 17.0 mins). Colourless oil.

1. G. D. Brown. "Two new compounds from *Artemisia annua*". *J. Nat. Prod.* **55** 1756-1770 (1992).

5-Nonadecyl resorcinol-3-*O*-methyl ether (**35**) (known^1^) – see Supplementary List 1 (Artemis) for NMR data

1.G. D. Brown. "Two new compounds from *Artemisia annua*". *J. Nat. Prod.* **55** 1756-1770 (1992).

*vii) Phenylpropanoids*

Coumarin (**37**) (known) – see Supplementary List for 1 (Artemis) for NMR data

*Coumarin* (**37**) was purified from fraction H by HPLC (15% EtOAc/hexane/0.5% AcOH) yielding HPLC fraction 24 (*R_t_* 21.9 mins). Colourless oil. ESI-MS (*m/z*): 147.0441, C_9_H_7_O_2_ [M + H^+^], ∆ = -1.2 ppm (R_t_ 4.08 mins).

Scopoletin (**38**) (known^1^) – see Supplementary List 1 (Artemis) for NMR data

*Scopoletin (****38****)* was purified from fraction M by HPLC (50% EtOAc/hexane/1% AcOH) yielding HPLC fraction 16 (*R_t_* 20.0 mins). Colourless oil. ESI-MS (*m/z*): 223.0601, C_11_H_11_O_5_ [M + H^+^], ∆ = 0.1 ppm (R_t_ 3.12 mins).

1. D. V. Banthorpe; G. D. Brown. "Two unexpected coumarins from tissue cultures of Compositae species". *Phytochemistry* **28** 3003-3007 (1989).

Isofraxidin (**39**) (known^1^) – see Supplementary List 1 (Artemis) for NMR data

1. D .V. Banthorpe; G. D. Brown. "Two unexpected coumarins from tissue cultures of Compositae species". *Phytochemistry* **28** 3003-3007 (1989).

*viii) Flavonoids*

5-Hydroxy-3,6,7-trimethoxy-2-(4’-methoxyphenyl)-4*H-*chromen-4-one (**85**) (known^1^)

NMR data for 5-hydroxy-3,6,7-trimethoxy-2-(4’-methoxyphenyl)-*4H*-chromen-4-one (**85**) in CDCl_3_ solution

| **Position** | **δ_H_ (ppm)** | **δ_C_ (ppm)** | **HMBC** | **^1^H-^1^H COSY** |
| --- | --- | --- | --- | --- |
| 2 | - | 156.3 | - | - |
| 3 | - | 138.9 | 3.86 (3-OMe) | - |
| 4 | - | 179.1 | - | - |
| 4a | - | 106.8 | - | - |
| 5 | - | 153.0 | - | - |
| 6 | - | 132.4 | 3.93 (6-OMe) | - |
| 7 | - | 158.9 | 3.96 (7-OMe) | - |
| 8 | 6.52 [1H], s | 90.5 | - | - |
| 8a | - | 152.6 | - | - |
| 1’ | - | 122.9 | - | - |
| 2’ | 8.08 [1H], d, *J* = 8 Hz | 130.4 | - | 7.04 (H-3’) |
| 3’ | 7.04 [1H], d, *J* = 8 Hz | 114.3 | - | 8.08 (H-2’) |
| 4’ | — | 161.9 | 3.91 (4’-OMe) | - |
| 5’ | 7.04 [1H], d, *J* = 8 Hz | 114.3 | - | 8.08 (H-6’) |
| 6’ | 8.08 [1H], d, *J* = 8 Hz | 130.4 | - | 7.04 (H-5’) |
| 3-OMe | 3.86 [3H], s | 60.3 | - | - |
| 6-OMe | 3.93 [3H], s | 61.1 | - | - |
| 7-OMe | 3.96 [3H], s | 56.5 | - | - |
| 4’-OMe | 3.91 [3H], s | 55.6 | - | - |

*5-Hydroxy-3,6,7-trimethoxy-2-(4’-methoxyphenyl)-4H-chromen-4-one (****85****)* was purified from fraction J by HPLC (25% EtOAc/hexane/0.5% AcOH) yielding HPLC fraction 18 (*R_t_* 21.9 mins); and from fraction K by HPLC (40% EtOAc/hexane/1% AcOH) yielding HPLC fraction 13 (*R_t_* 12.8 mins). Colourless oil. ESI-MS (*m/z*): 359.1125, ∆ = 0.4 ppm for [M + H^+^] (calc. for C_19_H_19_O_7_); R_t_ 7.08 mins. HRAPCIMS (*m/z*): 359.1143, Δ = -5.0 ppm for [M + H]^+^ (calc. 359.1125 for C_19_H_19_O_7_).

1. L.-K. Sy; G. D. Brown “Three sesquiterpenes from *Artemisia annua*”. *Phytochemistry* **48**(7) 1207-1211 (1998).

Chrysosplenetin (**40**) (known^1^) – see Supplementary List 1 (Artemis) for NMR data

*Chrysosplenetin* (**40**) was purified from fraction M by HPLC (50% EtOAc/hexane/1% AcOH) yielding HPLC fraction 13 (*R_t_* 16.2 mins). Colourless oil.

1. L.-K. Sy; G. D. Brown “Three sesquiterpenes from *Artemisia annua*”. *Phytochemistry* **48**(7) 1207-1211 (1998).

Casticin (**41**) (known^1^) – see Supplementary List 1 (Artemis) for NMR data

*Casticin (****41****)* was purified from fraction M by HPLC (50% EtOAc/hexane/1% AcOH) yielding HPLC fraction 14 (*R_t_* 16.6 mins). Colourless oil.

1. G. D. Brown; G.-Y. Liang; L.-K. Sy. “Terpenoids from the seeds of *Artemisia annua*”. *Phytochemistry* **64** 303-323 (2003).
